# *LINC01021* Attenuates Expression and Affects Alternative Splicing of a Subset of p53-Regulated Genes

**DOI:** 10.3390/cancers16091639

**Published:** 2024-04-24

**Authors:** Markus Kaller, Ignasi Forné, Axel Imhof, Heiko Hermeking

**Affiliations:** 1 Experimental and Molecular Pathology, Institute of Pathology, Faculty of Medicine, Ludwig-Maximilians-Universität München, Thalkirchner Strasse 36, D-80337 Munich, Germany; 2 BioMedical Center, Faculty of Medicine, Ludwig-Maximilians-Universität München, Grosshaderner Strasse 9, D-82152 Planegg-Martinsried, Germany; 3 German Cancer Consortium (DKTK), Partner Site Munich, D-69120 Heidelberg, Germany; 4 German Cancer Research Center (DKFZ), D-69120 Heidelberg, Germany

**Keywords:** p53, *LINC01021*, *PURPL*, lncRNA, tumor suppression

## Abstract

**Simple Summary:**

Growing evidence indicates that p53-induced long noncoding (lnc) RNAs constitute an elaborate regulatory network that mediates and/or modulates p53 function and, thus, tumor suppression. p53-induced *LINC01021* has been suggested to represent a negative feedback regulator of p53 protein stability under non-stress conditions. Furthermore, the loss of *LINC01021* in p53-proficient colorectal cancer (CRC) cell lines results in increased sensitivity to DNA-damaging chemotherapeutics. In order to analyze whether *LINC01021* affects the transcriptional program of p53 independently from the direct feedback regulation of p53, we studied the effect of CRISPR/Cas9-mediated abrogation of p53-induced *LINC01021* transcription on genome-wide RNA expression changes after the activation of ectopic p53 in CRC cells by RNA-Seq analyses. Our results demonstrate diverse regulatory effects of *LINC01021* on a subset of p53-regulated genes either via attenuated expression or altered transcript isoform usage. Taken together, our study provides a comprehensive framework and resource for further analyses of *LINC01021* function downstream of p53.

**Abstract:**

Background: Loss of the p53-inducible *LINC01021* in p53-proficient CRC cell lines results in increased sensitivity to DNA-damaging chemotherapeutics. Here, we comprehensively analyze how *LINC01021* affects the p53-induced transcriptional program. Methods: Using a CRISPR/Cas9-approach, we deleted the p53 binding site in the *LINC01021* promoter of SW480 colorectal cancer cells and subjected them to RNA-Seq analysis after the activation of ectopic p53. RNA affinity purification followed by mass spectrometry was used to identify proteins associated with *LINC01021*. Results: Loss of the p53-inducibility of *LINC01021* resulted in an ~1.8-fold increase in the number of significantly regulated mRNAs compared to *LINC01021* wild-type cells after ectopic activation of p53. A subset of direct p53 target genes, such as *NOXA* and *FAS,* displayed significantly stronger induction when the p53-inducibility of *LINC01021* was abrogated. Loss of the p53-inducibility of *LINC01021* resulted in alternative splicing of a small number of mRNAs, such as *ARHGAP12*, *HSF2*, and *LYN*. Several RNA binding proteins involved in pre-mRNA splicing were identified as interaction partners of *LINC01021* by mass spectrometry. Conclusions: Our results suggest that *LINC01021* may restrict the extent and strength of p53-mediated transcriptional changes via context-dependent regulation of the expression and splicing of a subset of p53-regulated genes.

## 1. Background

The *TP53* tumor-suppressor gene represents the most commonly mutated gene in human cancer [1]. It encodes the p53 transcription factor, which is activated by various forms of cellular stress, such as DNA damage induced by irradiation or aberrant oncogene activation. P53 regulates a large set of genes, which regulate numerous cellular functions that mediate tumor suppression by p53, such as cell cycle arrest, apoptosis, senescence, and DNA repair [2,3,4]. Apart from the many protein coding genes that have been functionally characterized as direct p53 targets, genes encoding noncoding RNAs are also directly regulated by p53. These include microRNAs (miRNAs) as important mediators of the repression of mRNA and protein expression caused by p53, thereby inhibiting pro-tumorigenic processes, such as proliferation, stemness, and epithelial–mesenchymal transition (EMT) [5]. Moreover, a growing number of long noncoding RNAs (lncRNAs) are induced by p53 (reviewed in [6,7,8]). LncRNAs are defined by a length of >200 nucleotides, which distinguishes them from small noncoding RNAs, such as miRNAs. Recent estimations indicate that, depending on the annotation method, the human genome harbors 15,000–100,000 lncRNA genes, which therefore represent a large class of transcripts most probably out-numbering protein-coding mRNAs [9,10]. However, the function and biological relevance of the vast majority of these transcripts is not well understood [11,12]. LncRNA’s function in many instances may be sequence-independent and solely linked to the act of its transcription, thereby, e.g., causing local alterations in chromatin structure due to nucleosome re-positioning associated with transcriptional elongation or RNA polymerase II-mediated replacement of DNA-bound transcription factors and, thus, the regulation of nearby genes in *cis* [13]. The sequence-dependent functions of lncRNAs that act in *trans* are diverse, ranging from the regulation of chromatin structure and the sequestration of microRNAs as so-called competitive endogenous RNAs (ceRNAs) to the regulation of mRNA stability, processing, or translation and the modulation of protein–protein interactions [14,15,16]. Numerous lncRNA genes have been identified as direct p53 targets by genome-wide studies in both murine and human cells [17,18,19,20,21,22], and a growing number of these lncRNAs have important roles in the p53 transcriptional network as both positive and negative regulators of p53 function (reviewed in [6,7,8]). 

We and others previously reported that *LINC01021* (also known as *PURPL* for *p53* up-regulated regulator of *p53* levels) is a p53-inducible lncRNA that affects the cellular response to genotoxic drugs, since the loss of *LINC01021* in *p53*-proficient colorectal cancer (CRC) cell lines resulted in increased sensitivity to DNA-damaging chemotherapeutics, suggesting a pro-survival function of *LINC01021* [23,24,25]. More recently, up-regulation and a potential pro-tumorigenic function of *LINC01021* have also been described in liver and gastric cancer [26,27]. However, the findings regarding the molecular mechanism(s) underlying *LINC01021* function have been conflicting. In CRC cell lines, the direct, negative feedback of *LINC01021* to p53 via HuR/MYBBP1A-mediated regulation of basal p53 protein levels/stability in the absence of stress has been proposed previously [25], which, however, was not observed in cell lines derived from liver tumors [28]. We could not detect an influence of *LINC01021* on basal and induced p53 levels and activity in CRC cells [24]. Nevertheless, we observed that experimental inactivation of *LINC01021* leads to increased chemotherapy-induced apoptosis in the *p53*-proficient HCT116 CRC cell line, suggesting that *LINC01021* may regulate the p53-induced transcriptional program without affecting p53 levels and activity directly [24].

Here, we study the role of *LINC01021* induction after the activation of p53 on a transcriptome-wide scale. We found that *LINC01021* restricts the extent and strength of p53-mediated transcriptional changes in a subset of p53-regulated genes. RNA expression signatures obtained after the abrogation of p53-induced *LINC01021* transcription were highly enriched for potential HDAC3 targets. Furthermore, loss of the p53-inducibility of *LINC01021* could at least in part be mimicked by pharmacological inhibition of HDAC3, suggesting HDAC3 as a potential mediator of *LINC01021* function. In addition, the loss of *LINC01021* affected the p53-regulated alternative splicing of a select number of mRNAs, thereby presumably inducing switches in the ratio of protein isoforms.

## 2. Materials and Methods

### 2.1. Cell Culture

Polyclonal cell pools of the colorectal cancer cell line SW480 harboring the episomal pRTR-*p53* vector [23], its single-cell derived subclones, and SW480 harboring pRTR-*LINC01021* were cultured in DMEM medium with 10% FCS (Invitrogen, Waltham, MA, USA), penicillin/streptomycin (10 units/mL), and 5% CO_2_. For the conditional expression of p53 or *LINC01021* from the pRTR vector, doxycycline (Sigma-Aldrich, St. Louis, MO, USA) was used at a final concentration of 100 ng/mL. The EZH2 inhibitor GSK343 (S7164, Selleckchem, Houston, TX, USA) was used at a concentration of 6 µM. The HDAC3 inhibitor RGFP966 (S7229, Selleckchem, Houston, TX, USA) was used at a concentration of 80 nM.

### 2.2. CRISPR/Cas9-Mediated Inactivation of LINC01021

Inactivation of *LINC01021* in SW480-pRTR-*p53* cells by *CRISPR*/Cas9 was carried out as described previously [24]. Briefly, we designed two guide RNAs targeting genomic regions flanking the p53 binding site in the *LINC01021* promoter. The oligonucleotides used for single guide (sg)RNA design are listed in Supplementary Table S1. SW480-pRTR-*p53* cells were transfected with 2.5 μg of each pSp-Cas9-sgRNA-GFP plasmid, or mock transfected with “empty” pSp-Cas9-GFP [29]. A total of 48 h post transfection, GFP-positive cells were sorted into 96 wells using a FACSARIA cell sorter (BD Biosystems) and expanded as single-cell clones for two weeks. Importantly, after clonal expansion, transient GFP expression from the pSp-Cas9-sgRNA-GFP plasmid was not detectable. Mock transfected cells were treated in a similar manner to obtain *LINC01021* wild-type single-cell clones. Genomic DNA of individual clones was screened by PCR for appropriate deletions using primers as described previously [24]. Oligonucleotides used for genotyping are listed in Supplementary Table S2. PCR products were Sanger-sequenced to verify the deletion of the p53 binding site. Clones with appropriate deletions within the *LINC01021* promoter (designated as “knock-out”, KO) were subsequently analyzed by qRT-PCR to verify the loss of *LINC01021* expression.

### 2.3. Ectopic Expression of LINC01021

The *LINC01021* transcript (NCBI transcript NR_038848) was amplified by PCR from cDNA obtained from SW480/pRTR-*p53* cells treated with DOX for 30 h, cloned into pGEM-T-Easy, and verified by Sanger sequencing. Subsequently, the *LINC01021* cDNA was transferred into the pRTR vector as described previously [30,31]. Oligonucleotides used for cloning are provided in Supplementary Table S2.

### 2.4. RNA Affinity Purification (RAP)

RNA affinity purification was performed according to the protocol by Nötzold et al. [32]. Briefly, SW480/pRTR-*LINC01021* or SW480/pRTR-*p53* cells were treated with DOX for 48 h before harvesting. Per sample, 2 × 10^7^ cells were fixed with 3% formaldehyde for 10 min at room temperature. RaPOOLs consisting of 30 biotinylated DNA oligonucleotides targeting all RNA splice variants of *LINC01021* were synthesized by siTOOLs (Martinsried, Germany). For affinity purification, 100 pmol raPOOL was used per sample. Hybridization was carried out at 37 °C for 4 h with agitation. Probe capture was performed with magnetic Dynabeads MyOne Streptavidin C1 beads (Thermo Fisher Scientific, Waltham, MA, USA) for 30 min at 37 °C. After washing five times for 5 min at 37 °C with 2xSSC, beads were washed an additional three times with 100 µL 50 mM Tris-HCl pH8 to remove all detergents and stored at −20 °C. As a control, a 10% fraction of the beads with bound RNA–protein complexes were subjected to RNA isolation followed by cDNA synthesis and qRT-PCR analysis to verify the enrichment of *LINC01021* RNA. 

### 2.5. Liquid Chromatography–Mass Spectrometry (LC-MS) Analysis

Mass spectrometry-based proteomic experiments were performed as described previously [33] with minor modifications. Briefly, beads were washed three times with 50 mM ammonium bicarbonate and incubated with 10 ng/µL trypsin in 1M urea 50 mM ammonium bicarbonate for 30 min, then washed with 50 mM ammonium bicarbonate and the supernatant was digested overnight in the presence of 1 mM DTT. Digested peptides were alkylated and desalted prior to LC-MS analysis. The peptide mixtures were subjected to nanoRP-LC-MS/MS analysis on an Ultimate 3000 nano chromatography system (Thermo Fisher Scientific) equipped with a 25 cm Aurora column (Ionopticks, Melbourne, Australia) and coupled to an Orbitrap Exploris-480 mass spectrometer (Thermo Fisher Scientific) operated in data-dependent mode to automatically switch between full scan MS and MS/MS acquisition. Survey full scan MS spectra (from *m*/*z* 350 to 1200) were acquired with resolution R = 60,000 at *m*/*z* 400 (AGC target of 3 × 10^6^). The 20 most intense peptide ions with charge states between 2 and 6 were sequentially isolated to a target value of 1 × 10^5^ and fragmented at 30% normalized collision energy. Typical mass spectrometric conditions were as follows: spray voltage, 1.5 kV; no sheath and auxiliary gas flow; heated capillary temperature, 275 °C; intensity selection threshold, 3 × 10^5^. MaxQuant [34] 2.1.0.0 was used to identify proteins and quantify them using iBAQ with the following parameters: Database Uniprot_UP000005640_Hsapiens_20210521.fasta; MS tol, 10 ppm; MS/MS tol, 20 ppm Da; Peptide FDR, 0.1; Protein FDR, 0.01 min; peptide length, 7; variable modifications, Oxidation (M); fixed modifications, Carbamidomethyl (C); peptides for protein quantitation, razor and unique; min. peptides, 1; min. ratio count, 2. For display and analysis, the Perseus v2.0.9.0 software [35] was used. The mass spectrometry proteomics data have been deposited to the ProteomeXchange Consortium via the PRIDE [36] partner repository with the dataset identifier PXD050892.

### 2.6. RNA isolation and qRT-PCR Analysis

For RNA-seq analysis, 2 × 10^5^ cells from three SW480-pRTR-*p53 LINC01021* wild-type and KO clones were seeded in 6-well format. After 24 h, medium was replaced with fresh medium without DOX or with DOX at a final concentration of 100 ng/mL. Cells were harvested after 30 h with an RNAeasy kit (QIAGEN, Hilden, Germany) according to manufacturer’s instructions. cDNA was generated from 1 μg of total RNA per sample using anchored oligo(dT) primers (Verso cDNA Kit, Thermo Scientific). qRT-PCR was performed by using a LightCycler 480 (Roche, Basel, Switzerland) and Fast SYBR Green Master Mix (Applied Biosystems, Foster City, CA, USA). Oligonucleotides used for qRT-PCR are listed in Supplementary Table S3.

### 2.7. Western Blot Analysis

Cells were lysed in RIPA lysis buffer (50 mM Tris/HCl, pH 8.0, 250 mM NaCl, 1% NP40, 0.5% sodium deoxycholate, 0.1% sodium dodecylsulfate, complete mini protease inhibitor tablets (Roche)). Lysates were sonicated and centrifuged at 16,060× *g* for 15 min at 4 °C. A total of 20 μg of whole cell lysate per lane were separated using 10% SDS-acrylamide gels and transferred on Immobilon PVDF membranes (Millipore, Burlington, MA, USA). Antibodies used were specific for p53 (DO-1), VSV (V4888, Sigma-Aldrich, St. Louis, MO, USA), and β-actin (A2066, Sigma-Aldrich).

### 2.8. Cell cycle Analysis by Propidium Iodide (PI) Staining

The 2 × 10^5^ cells from SW480-pRTR-*p53 LINC01021* wild-type and KO clones were seeded in 6-well format. DOX was added after 24 h for 24 h. Both the supernatant and adherent cell fractions were collected and combined after trypsinization. Cells were washed once in HBSS and fixed in ice-cold ethanol (70%) overnight at −20 °C. Fixed cells were washed with PBS and resuspended in PI staining solution. Cell cycle distribution of the cells was measured using an Accuri™ C6 flow cytometry instrument (BD Biosciences, Franklin Lakes, NJ, USA) and analyzed with the BD Accuri C6 v1.0.264.21 software.

### 2.9. Bioinformatic Analysis of RNA-Seq Data

Total RNA from three SW480-pRTR-53 *LINC01021* wild-type and KO clones was used for RNA-Seq. Random primed cDNA libraries were constructed and sequenced using the HiSeq4000 (Illumina, San Diego, CA, USA) platform by GATC (Konstanz, Germany). Each sample was covered by at least 30 million single reads of 50 bp length. Reads were processed using the RNA-Seq module implemented in the CLC Genomics Workbench v20.0.2 software (QIAGEN Bioinformatics, Dusseldorf, Germany) and mapped to the HG38 human reference genome and its associated gene and transcript annotation (ENSEMBL) with the following settings: mismatch cost = 2, insertion cost = 2, deletion cost = 3, length fraction = 0.8, similarity fraction = 0.8. RNA-Seq data were filtered to exclude weakly expressed transcripts with less than two mapped exon reads in all samples from the analysis and subjected to upper quartile normalization using the R/Bioconductor RUVSeq (*r*emove *u*nwanted *v*ariation from RNA-Seq data) package as described in [37]. For statistical analyses, control and DOX-treated samples from three *LINC01021* wild-type and KO clones, respectively, were treated as replicates. Differential gene and transcript isoform expression analysis was performed with edgeR [38,39] and DESeq2 [40] after further normalization using the RUVg approach described in [37] to remove variation between RNA samples resulting from differences in library preparation. Genes and transcript isoforms with significant differences in regulation between *LINC01021* wild-type and KO cells were determined with the LIMMA R package using a 2 × 2 factorial interaction model [41]. Gene set enrichment of Hallmark and other gene sets was analyzed using the Molecular Signatures database (MSigDB) [42]. Direct p53 target genes were defined by the “Fischer Direct p53 Targets Meta Analysis” gene set from MSigDB based on [43].

### 2.10. Statistical Analysis

A Student´s *t*-test (unpaired, two-tailed) was used to determine significant differences between two groups of samples. *p*-values < 0.05 were considered significant (*: *p* < 0.05; **: *p* < 0.01; ***: *p* < 0.001).

## 3. Results

**Figure 1:**
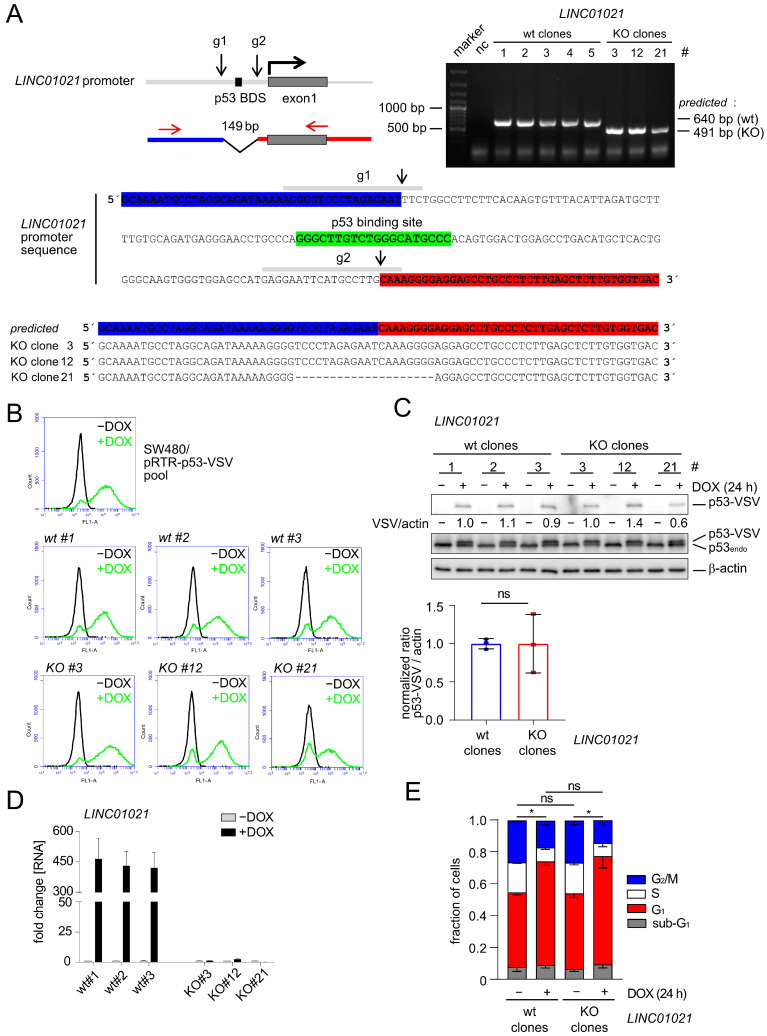
Effects of deleting the p53 binding site within the *LINC01021* promoter in the CRC cell line SW480. (**A**) Upper left: schematic depiction of the *LINC01021* promoter, the p53 binding site, and the regions targeted by CRISPR/Cas9. Upper right: gelelectrophoretic separation of the PCR products obtained using primers, shown by red arrows. Lower part: Sequence of the *LINC01021* promoter region. The binding sites of the guide RNAs (g1 and g2) are depicted as grey horizontal bars. The Cas9 cleavage sites are indicated with black arrows. The p53 binding site is shown in green. Deletions of the p53 BDS in three KO clones were further characterized by Sanger sequencing of the PCR products. (**B**) FACS analysis of GFP expression after treatment of the indicated cells with DOX for 48 h. (**C**) Western Blot analysis with antibodies directed against ectopic (p53-VSV) and both ectopic and endogenous mutant (p53_endo_) p53 proteins after treatment with DOX for 24 h. Quantification of ectopic p53-VSV in *LINC01021* wild-type and KO clones represents mean +/− s.d. p53-VSV signal normalized to β-actin. ns: not significant. (**D**) qRT-PCR analysis of *LINC01021* after treatment with DOX for 24 h. Results are represented as mean +/− s.d. (*n* = 3). (**E**) Cell cycle distribution after treatment of the indicated cells with DOX for 24 h was determined by flow cytometry after the staining of DNA with propidium iodide. Results represent mean +/− s.d. obtained from three *LINC01021* wild-type (# 1, 2, and 3) and KO clones (# 3, 12, and 21). Statistically significant (*p* < 0.05) differences in the fraction of G_1_, S, and G_2_/M cells are indicated with asterisks. *: *p* < 0.05; ns: not significant.

In order to determine the role of *LINC01021* in the transcriptional regulations that occur after the activation of p53, we decided to delete the p53 binding site within the *LINC01021* promoter. Therefore, we generated a small (~149 base pair) deletion of the genomic region encompassing the p53 binding site (p53 BDS) in the *LINC01021* promoter by using a CRISPR/Cas9 approach in SW480-pRTR-*p53* cells (Figure 1A). These cells harbor an episomal pRTR vector system for the conditional expression of both GFP and a VSV-tagged *p53* allele from a bidirectional promoter with doxycycline (DOX) and have been employed previously by us for the comprehensive analysis of the p53-regulated transcriptome and the identification of p53-induced lncRNAs [23,24] (Figure 1B). After single cell sorting and clonal expansion, we obtained three clones harboring appropriate deletions of the p53 binding site within the *LINC01021* promoter, as well as three wild-type clones (Figure 1A). The inducibility of GFP encoded by the pRTR vector by DOX was highly similar in all *LINC01021* wild-type and KO subclones and was comparable to the parental SW480-pRTR-*p53*-VSV cell pool (Figure 1B). Moreover, all *LINC01021* wild-type and KO subclones showed comparable induction of ectopic VSV-tagged p53 protein expression upon the addition of DOX (Figure 1C). Therefore, we concluded that potential heterogeneity between individual clones due to differences in the induction of ectopic p53 was minimal. The CRC cell line SW480 harbors three copies of endogenous *p53*, each carrying R273H and P309S mutations (Figure 1C). The R273H mutation represents a DNA contact mutation, which renders the p53 protein largely inert with regard to the activation of canonical p53 target genes [44]. Therefore, the basal expression of *LINC01021* is very low in these cells but is induced dramatically upon activation of the ectopic *p53* allele with DOX [23,24]. While wild-type clones showed similar, strong induction of *LINC01021* after the addition of DOX for 24 h, deletion of the p53 binding site completely abrogated *LINC01021* induction (Figure 1D). Therefore, this system allows one to analyze the effect of loss of *LINC01021* induction in a highly controlled manner, i.e., by minimizing the potential effects of *LINC01021* loss on basal p53 transcriptional activity under non-stress conditions, as observed by others [25].

The p53-mediated cell cycle arrest in the G_1_ phase observed 24 h after the addition of DOX to SW480-pRTR-*p53*-VSV cells was largely identical in the three *LINC01021* wild-type clones when compared to three KO clones (Figure 1E), indicating that the canonical effect of p53 activation in these cells was not altered by the loss of *LINC01021* induction.

**Figure 2:**
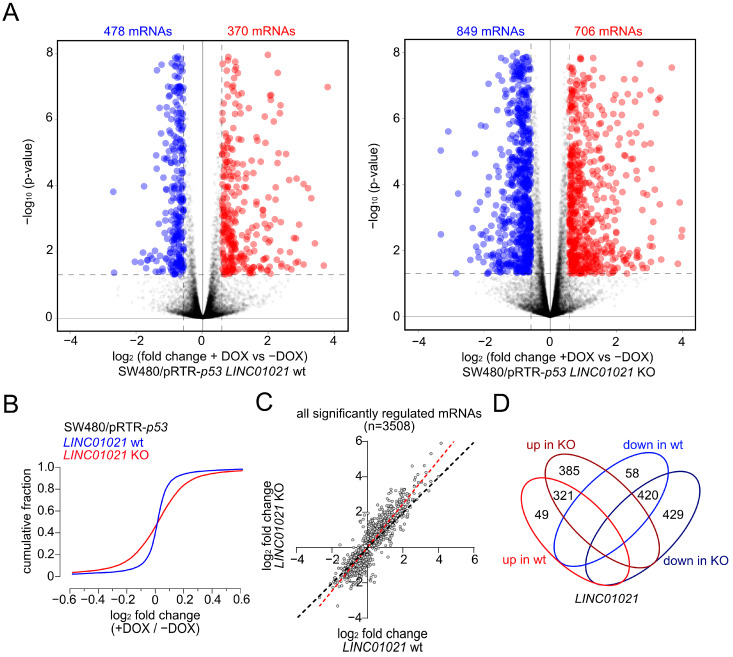
Loss of p53inducibility of *LINC01021* enhances differential gene regulation by p53. (**A**) Volcano plots displaying significantly up- and down-regulated mRNAs upon activation of p53 with DOX in *LINC01021* wild-type clones (# 1, 2, 3; left) and *LINC01021* KO clones (#3, 12, 21; right). (**B**) Global comparison of p53-induced mRNA expression changes in *LINC01021* wild-type and KO clones by cumulative distribution analysis. (**C**) Scatter plot displaying log_2_-transformed fold changes in significantly regulated mRNAs after activation of p53 in *LINC01021* wild-type and/or KO clones. The linear regression line is shown in red. The black dotted line indicates identical fold changes between *LINC01021* wild-type and KO clones. (**D**) Venn diagram displays overlaps between significantly regulated mRNAs in *LINC01021* wild-type and/or KO clones.

For RNA-Seq analyses, total RNA was harvested from untreated controls and after the induction of p53 with DOX for 30 h from the three *LINC01021* wild-type and three KO clones. The poly-A-RNA-enriched fraction of one untreated control sample (-DOX) and one DOX-treated sample (+DOX) from each clone was used for library generation and sequencing. In the wild-type clones, 370 and 478 mRNAs were significantly up- and down-regulated, respectively, with a fold change (+DOX vs. −DOX) ≥ 1.5, while in the KO clones, 706 mRNAs were significantly up-regulated and 849 were down-regulated (Figure 2A, Supplementary Table S4). Thus, *LINC01021* KO clones displayed an ~1.8-fold increase in the number of significantly regulated mRNAs when compared to wild-type clones upon activation of p53. Moreover, a comparison of the cumulative distributions of gene expression changes in *LINC01021* wild-type and KO clones showed a general broadening of expression changes in *LINC01021* KO clones, suggesting a global amplification of the response to the ectopic activation of p53 in these cells (Figure 2B). Therefore, we asked whether the expression changes in each mRNA are more pronounced in KO cells when compared to wild-type cells upon activation of p53, thus leading to the observed increase in mRNAs that passed our cut-off criterion (fold change +DOX vs. −DOX ≥ 1.5) for differential expression. We compared the expression changes in all mRNAs that were significantly regulated in either *LINC01021* wild-type and/or KO cells (Figure 2C). Indeed, we found that p53-induced expression changes per *mRNA* were generally enhanced in *LINC01021* KO cells compared to wild-type cells for the vast majority of significantly regulated mRNAs (Figure 2C). Moreover, the vast majority of genes regulated in *LINC01021* wt clones were also significantly regulated in *LINC01021* KO clones (87% (321/370) of up- and 88% (420/478) of down-regulated genes). Notably, none of the genes significantly either up- or down-regulated in *LINC01021* wt clones showed the opposite type of regulation in *LINC01021* KO clones, indicating that the loss of *LINC01021* augments the response to ectopic activation of p53 in these cells, but not the direction of regulation (Figure 2D). Therefore, these results imply that *LINC01021* dampens the extent to which genes may be regulated by p53.

**Figure 3:**
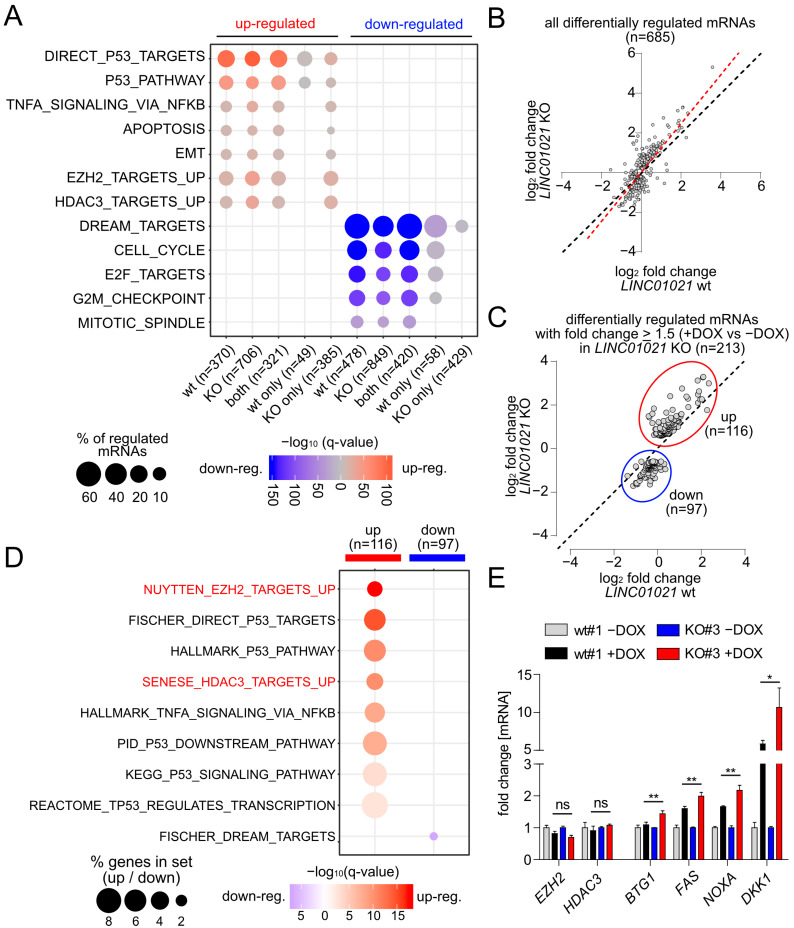
Specific genes and pathways are affected by loss of p53-inducibility of *LINC01021*. (**A**) Identification of over-represented p53-related and additional functional gene categories (MSigDB) among mRNAs up- and down-regulated (shown in red and blue, respectively) after p53 activation in *LINC01021* wild-type and/or KO clones. Dot size indicates percentage of respective gene sets among differentially expressed mRNAs and dot color statistical significance of overlap. (**B**) Scatter plot displaying log_2_-transformed fold changes in all mRNAs showing *LINC01021*-dependent differential regulation between *LINC01021* wild-type (#1, 2, and 3) and KO (#3, 12, and 21) clones after activation of p53 by DOX. Linear regression line is shown in red. Black dotted line indicates identical fold changes in *LINC01021* wild-type and KO cells. (**C**) Scatter plot displaying log_2_-transformed fold changes in all mRNAs showing *LINC01021*-dependent differential regulation between *LINC01021* wild-type (#1, 2, and 3) and KO (#3, 12, and 21) clones and significant regulation (fold change ≥ 1.5) in *LINC01021* KO clones after activation of p53 by DOX. (**D**) Identification of over-represented functional categories among mRNAs showing *LINC01021*-dependent differential regulation between *LINC01021* wild-type (#1, 2, and 3) and KO (#3, 12, and 21) clones and significant regulation (fold change ≥ 1.5) in *LINC01021* KO clones after p53 activation. (**E**) qRT-PCR analysis of selected genes in indicated cells after activation of p53 by DOX for 30 h. Results from one representative wild-type and KO clone are shown and represent mean +/− s.d. (*n* = 3). *: *p* < 0.05; **: *p* < 0.01. ns: not significant.

A gene set enrichment analysis (GSEA) of the most significantly over-represented gene sets among all significantly regulated mRNAs in *LINC01021* wild-type and KO clones showed that direct p53 targets and other signatures related to p53 function, such as apoptosis and EMT, were highly enriched among the up-regulated mRNAs in both *LINC01021* wild-type and KO clones (Figure 3A). Likewise, the majority of genes down-regulated in both *LINC01021* wild-type and KO clones were targets of the p53-p21-DREAM complex involved in the repression of G_2_/M cell cycle genes [45], reflecting the prominent cell cycle arrest observed in these cells upon activation of p53 (Figure 1E). Interestingly, we observed a decrease in the fraction of direct p53 targets and p53 pathway-related genes that are exclusively up-regulated in *LINC01021* KO clones, as well as a decrease in the fraction of cell-cycle-related genes exclusively down-regulated in *LINC01021* KO clones (Figure 3A). In contrast, mRNAs represented by the “TNFα Signaling Via NFKB” gene set were also enriched among the mRNAs exclusively regulated after loss of the p53-inducibility of *LINC01021*, indicating *LINC01021* may disproportionally affect the regulation of a subset of specific pathways.

Notably, mRNAs up-regulated in *LINC01021* KO but not in wild-type cells were highly enriched for genes up-regulated upon the siRNA-mediated depletion of EZH2 in the PC3 prostate cancer cell line (NYUTTEN_EZH2_UP, ref. [46]) or the depletion of HDAC3 in the U2OS osteosarcoma cell line (SENESE_HDAC3_UP, ref. [47]) (Figure 3A). Interestingly, EZH2 and HDAC3 represent two chromatin modifiers involved in the transcriptional repression of numerous target genes [48,49], suggesting that at least a subset of genes affected by loss of the p53-inducibility of *LINC01021* may also be regulated by EZH2 and/or HDAC3 and that *LINC01021* potentially cooperates with EZH2 and/or HDAC3 in the regulation of these genes.

**Figure 4:**
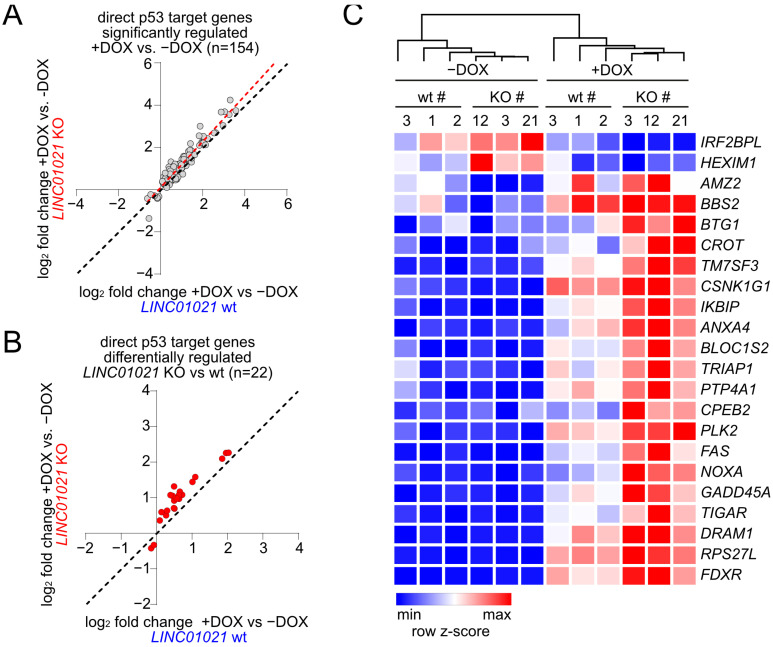
Identification of direct p53 target genes affected by loss of p53-inducibility of *LINC01021*. (**A**) Scatter plot displaying log_2_-transformed fold changes in all mRNAs encoded by direct p53 target genes significantly regulated in *LINC01021* wild-type (#1, 2, and 3) and/or KO (#3, 12, and 21) clones after activation of p53 by DOX. Linear regression line is shown in red. Black dotted line indicates identical fold changes in *LINC01021* wild-type and KO cells. (**B**) Scatter plot displaying log_2_-transformed fold changes in direct p53 target genes showing *LINC01021*-dependent differential regulation between *LINC01021* wild-type (#1, 2, and 3) and KO (#3, 12, and 21) clones after activation of p53 by DOX. Black dotted line indicates identical fold changes in *LINC01021* wild-type and KO cells. (**C**) Heatmap displaying hierarchical clustering of mRNAs encoded by direct p53 target genes displaying *LINC01021*-dependent differential regulation in *LINC01021* wild-type and KO clones after activation of p53.

Next, we determined those mRNAs that showed a significantly altered induction by p53 in *LINC01021* KO compared to wild-type cells. We obtained a set of 685 differentially regulated mRNAs, the large majority of which showed stronger expression changes (+DOX vs. −DOX) in *LINC01021* KO cells compared to wild-type cells (Figure 3B). Out of these, we determined all mRNAs that, apart from differential regulation between *LINC01021* wild-type and KO cells, also showed ≥1.5-fold regulation in *LINC01021* KO cells after the induction of p53 (Figure 3C). Thereby, we identified 116 up- and 97 down-regulated mRNAs with significant differences in p53-induced regulation (Supplementary Table S5). Next, we analyzed whether this set of differentially regulated mRNAs showed similarities to previously published gene expression signatures (Figure 3D). Remarkably, apart from p53-regulated genes, two of the most strongly enriched gene expression signatures among the differentially induced mRNAs were, again, those of RNAs up-regulated after the depletion of EZH2 and/or HDAC3. Taken together, these results indicate that after the activation of p53, *LINC01021* KO cells display stronger induction of genes that are presumably subject to epigenetic regulation by EZH2 and/or HDAC3. We further validated the differential regulation of several of these mRNAs in *LINC01021* KO vs. wild-type cells by qRT-PCR analysis (Figure 4B). Importantly, *EZH2* and *HDAC3* RNA levels were not significantly changed in *LINC01021* KO vs. wild-type cells, suggesting that the observed enrichment of EZH2- and HDAC3-associated gene signatures in *LINC01021* KO cells is likely not due to differences in *EZH2* and *HDAC3* expression levels.

mRNAs encoded by direct p53 target genes were highly over-represented among the differentially regulated mRNAs (Figure 3D). Out of 154 direct p53 target genes showing significant mRNA expression changes in either *LINC01021* wild-type and/or KO cells (Figure 4A), we identified 22 direct p53 target genes that showed significant differences in regulation between *LINC01021* wild-type and KO cells (Figure 4B). Notably, while several well-characterized p53 targets were among these genes, such as *PMAIP1*/*NOXA*, *FAS*, and others (Figure 4C), the majority of known p53 targets, including “classical” p53 target genes, such as *CDKN1A*/p21 and *MDM2*, did not show significantly stronger induction in *LINC01021* KO cells. This indicated that *LINC01021* does not modulate global p53 transcriptional activity, but affects the regulation of a specific subset of p53 target genes. 

**Figure 5:**
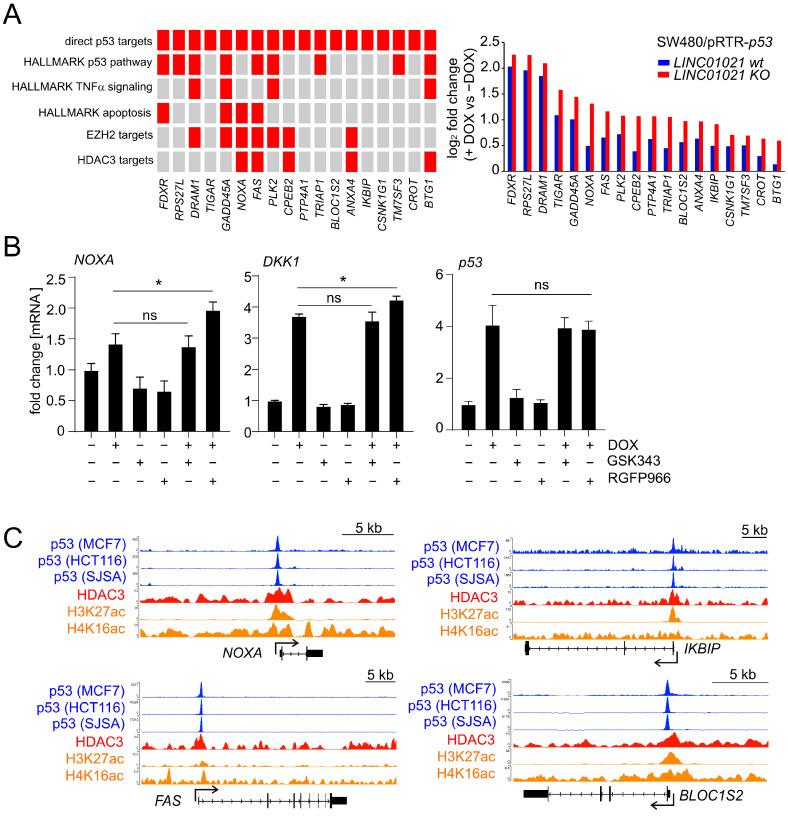
Loss of p53-inducibility of *LINC01021* affects genes coordinately regulated by p53 and HDAC3. (**A**) (**left**): grid representation of direct p53 targets with differential regulation by p53 between *LINC01021* KO and wild-type clones and their association with selected functional categories and up-stream regulators (indicated in red). (**right**): log_2_-fold changes in gene expression as determined by DESeq2. (**B**) qRT-PCR analysis of indicated genes after activation of p53 by DOX and/or inhibition of EZH2 (by GSK343) or HDAC3 (by RGFP966) in SW480/pRTR-*p53* for 30 h. Results are represented as mean +/− s.d. (*n* = 3). *: *p* < 0.05; ns: not significant. (**C**) Genomic profiles showing potential promoter co-occupancy of selected direct p53 target genes by p53 and HDAC3. ChIP-Seq data were obtained from previously published genome-wide studies [53,54] and visualized using the UCSC genome browser.

In order to gain further insight into the molecular mechanism underlying the differential regulation of a subset of direct p53 targets caused by loss of the p53-inducibility of *LINC01021*, we determined whether the direct p53 targets found to be differentially regulated here display an overlap with other gene sets significantly enriched among the differentially induced RNAs (Figure 5A). Notably, several of the differentially induced p53 target genes have also been reported to be regulated by the loss of EZH2 and/or HDAC3, suggesting a coordinated regulation of these genes by both p53 and chromatin modifiers (Figure 5A). Among these, the p53 targets *NOXA* and *FAS* have previously been shown to be directly regulated by HDACs [50,51]. Interestingly, interfering with HDAC3 activity using a highly selective small molecule inhibitor of HDAC3, RGFP966, also led to increased induction of *NOXA* after activation of p53. The inhibition of EZH2 using the small molecule inhibitor GSK343 had no effect on *NOXA* induction (Figure 5B). The p53-mediated induction of the WNT signaling inhibitor *DKK1*, which has previously been shown to be regulated by HDAC inhibition in several CRC cell lines, among them SW480 cells [52], was also increased upon the inhibition of HDAC3, but not by an EZH2 inhibitor. An analysis of *p53* expression by qRT-PCR with primers recognizing both endogenous and ectopic *p53* mRNA showed that treatment with an HDAC3 inhibitor alone did not affect the expression of endogenous *p53* in the absence of DOX. In addition, total RNA levels of both endogenous and ectopic *p53* in the presence of DOX were not affected by concomitant treatment with an HDAC3 inhibitor, ruling out differences in the transcription of ectopic *p53* from the pRTR vector due to the inhibition of HDAC3 as the cause of this effect (Figure 5B). Moreover, an analysis of previously published ChIP-Seq datasets of p53 and HDAC3 chromatin occupancy indicated that p53 and HDAC3 may co-occupy the promoter regions of several differentially induced direct p53 targets [53,54], supporting their coordinated regulation by p53 and HDAC3 (Figure 5C). 

**Figure 6:**
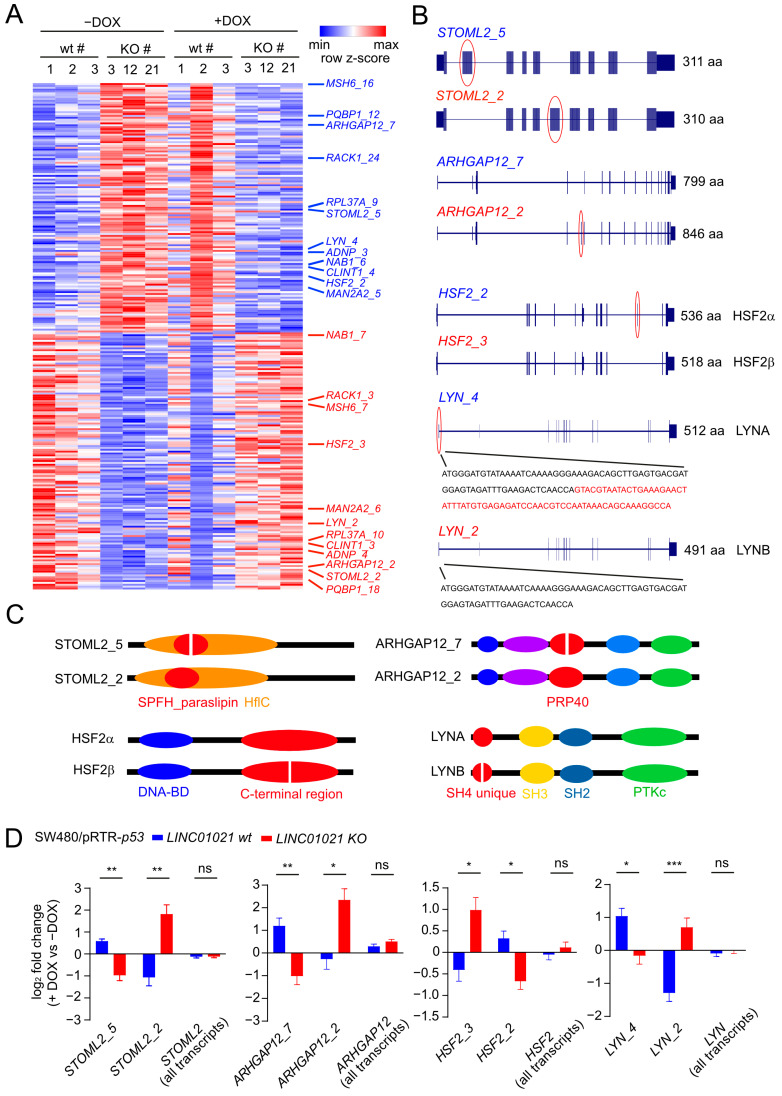
p53-mediated alternative splicing is affected by loss of p53-inducibility of *LINC01021*. (**A**) Heatmap visualization of transcript isoforms showing *LINC01021*-dependent differences in regulation in *LINC01021* wild-type and KO clones after activation of p53. Twenty-four transcript isoforms representing twelve pairs of transcript isoforms encoded by the same gene with different regulatory patterns are indicated on the right. (**B**) Gene structure ideograms of protein-coding transcript isoforms of ARGAP12, HSF2, and LYN with significant differences in regulation in *LINC01021* KO and wild-type clones after activation of p53. Exons differing between indicated transcript variants are encircled in red. Number of amino acids encoded by respective open reading frames are indicated. aa: amino acids. (**C**) Graphical overview of protein domains affected by alternative splicing events depicted in B. Protein domains altered by these splicing events are shown in red. (**D**) Log_2_-fold changes in transcript isoform expression as determined by DESeq2. The “all isoforms” bars indicate expression changes in the respective gene model comprising all annotated transcript variants. Statistical significance as determined by DESeq2 is indicated with *: *p* < 0.05; **: *p* < 0.01; ***: *p* < 0.001. ns: not significant.

p53 regulates the alternative splicing (AS) of numerous mRNAs via induction of the gene encoding the RNA-binding protein and splicing regulator ZMAT3 [55,56]. Interestingly, lncRNAs have also been reported to regulate AS by various direct and indirect mechanisms [57]. To identify mRNA isoform switches caused by AS which are potentially regulated by *LINC01021*, we analyzed expression changes in individual transcript isoforms after the activation of p53 in cells with and without p53-inducibility of *LINC01021*. Notably, the p53-mediated induction of *ZMAT3* mRNA was not significantly different between wild-type cells and cells with a loss of *LINC01021*-inducibility by p53 (Supplementary Table S4). In total, we identified 286 transcript isoforms that displayed significant differences in regulation between *LINC01021* wt and KO cells after the activation of p53 (Figure 6A, Supplementary Table S6). For 262 of these transcripts, additional transcripts encoded by the respective gene that showed significant differences in regulation between *LINC01021* wt and KO cells were not detected. Hence, the large majority of differentially regulated transcript isoforms were unlikely to result in isoform switches due to alternative splicing. However, 24 of the differentially regulated transcript isoforms represented 12 pairs of different mRNAs encoded by the same gene, which displayed opposite patterns of regulation in *LINC01021* wt and KO cells after the activation of p53 (Figure 6A). Interestingly, four of the identified pairs of differentially regulated mRNAs (*STOML2*, *ARHGAP12*, *HSF2*, *LYN*) represent alternative splicing events that give rise to different protein isoforms (Figure 6B), some of which have been reported previously [58,59,60]. Interestingly, each of the identified alternative splicing events leads to alterations of a specific protein domain encoded by the respective mRNA sequence (Figure 6C), suggesting that these isoform switches may be functionally relevant. Interestingly, no significant difference in the regulation of the total mRNA levels comprising all RNA isoforms of each of these genes could be detected, whereas the identified RNA isoforms displayed a significant opposite regulation in *LINC01021* wt and KO cells after the activation of p53 (Figure 6D). The role of these isoform switches in the context of p53 function, and their regulation by *LINC01021*, remains to be elucidated.

**Figure 7:**
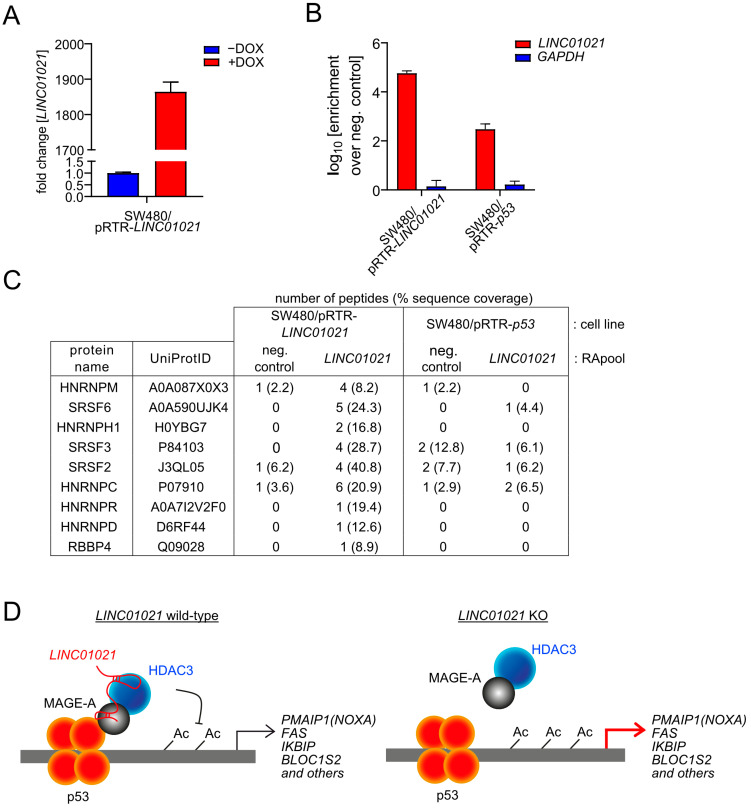
Potential protein interaction partners of *LINC01021* identified by RNA affinity purification followed by mass spectrometry (RAP-MS). (**A**) qRT-PCR analysis of ectopic *LINC01021* expression after activation by DOX for 48 h. (**B**) qRT-PCR analysis of *LINC01021* enrichment after RAP with *LINC01021* raPOOLs compared to non-specific raPOOLs (neg. control). *GAPDH* served as a control to verify *LINC01021*-specific enrichment. (**C**) Proteins co-purified with *LINC01021* as identified by LC-MS. The number of detected peptides per protein and their respective sequence coverage are indicated. (**D**) Hypothetical model of *LINC01021* as a molecular scaffold bridging p53 and HDAC3-containing protein complexes for recruitment to specific promoter regions.

In order to gain further insight into the molecular mechanism involved in the elevated induction of *NOXA* and other genes in *LINC01021*-deficient cells, we aimed to identify protein interaction partners of *LINC01021* by RNA affinity purification (RAP) followed by mass spectrometry (RAP-MS). While the fold change in *LINC01021* levels induced by p53 in SW480 cells is substantial, the absolute *LINC01021* levels remain rather low even after the activation of p53. Therefore, RNA pulldown of endogenous *LINC01021* may not be sufficient for the reliable detection of protein interaction partners by mass spectrometry. Therefore, we generated additional SW480 cell pools with inducible ectopic expression of *LINC01021*. For RNA pulldown, the ectopic expression of either *p53* (to pull down endogenous *LINC01021*) or *LINC01021* was induced by the addition of DOX for 48 h before harvesting cells (Figure 7A). The specific enrichment of *LINC01021* after affinity purification was verified by qRT-PCR analysis of the purified RNA eluate (Figure 7B). The RAP of endogenous *LINC01021* in SW480/pRTR-*p53* cells yielded a low number of *LINC01021*-associated proteins. However, in cells ectopically expressing *LINC01021*, we detected additional *LINC01021*-associated proteins (Figure 7C). Notably, we identified RBBP4 as an interaction partner of *LINC01021*, albeit by only one peptide. RBBP4 is a protein subunit of HDAC1/2-containing chromatin remodeling complexes, such as the Sin3 and NuRD complexes [61]. This may indicate that *LINC01021* indeed associates with HDAC-containing complexes, albeit not with HDAC3, which is part of the NCoR corepressor complex that does not contain RBBP4 [61]. Interestingly, several RNA binding proteins involved in pre-mRNA splicing were found to be associated with *LINC01021*, such as several heterogeneous nuclear ribonucleoproteins (HNRNPs) and the SRSF2/3/6 splicing regulators. Taken together, our results suggest that *LINC01021* may function as a molecular scaffold, which recruits HDAC-containing protein complexes to p53 at specific promoter regions, either directly or indirectly via MAGE-A proteins as reported for HDAC3 [62,63] (hypothetical model shown in Figure 7D). Alternatively, *LINC01021* may affect the splicing of a select number of mRNAs, for example via physical association with SRSF proteins, which has previously been shown for the DGCR5 lncRNA [64].

## 4. Discussion 

The transcriptomic profiles obtained after the ectopic activation of p53 in CRC cells in which *LINC01021* was rendered unresponsive to p53 demonstrate that *LINC01021* may function to limit the expression of a subset of p53 targets. Pathway analysis showed that the defect in the p53-induciblility of *LINC01021* had moderate effects on the expression of transcripts associated with the cellular processes most prominently regulated by the activation of p53, such as cell cycle arrest in the G_1_ phase, and resulted in more pronounced transcriptional changes in mRNAs associated with additional downstream pathways, such as TNFα signaling. Moreover, since the lack of p53-induced *LINC01021* was not accompanied by detectable differences in p53 levels, and only a subset of direct p53 targets showed significantly stronger induction in cells lacking p53-induced *LINC01021*, our results suggest that *LINC01021* does not directly regulate general p53 activity, which has been implied by others [25]. Indeed, more recent studies have shown that *LINC01021* regulates the fine-tuning of gene expression during p53 activation without the direct regulation of p53 [28], which is largely mirrored by our own results shown here. 

Interestingly, mRNAs preferentially induced by p53 in the absence of *LINC01021* activation showed a significant enrichment for mRNAs known to be activated after the depletion of the chromatin modifiers EZH2 and HDAC3. Moreover, pharmacological inhibition of HDAC3 resulted in enhanced activation of these mRNAs by p53. Interestingly, HDAC3 has been reported to interact with p53 and regulate its activity by deacetylation, either by direct binding to p53 or indirectly via MAGE-A proteins [62,63]. Therefore, a function of *LINC01021* may be to modulate transcriptional regulation by p53 via the recruitment of HDAC3-containing protein complexes to a subset of direct p53 target genes, and additional indirect effectors of the p53 response (see also Figure 7D). For example, *LINC01021* may function as a molecular scaffold, which recruits HDAC3 to p53 at specific promoter regions, either directly or indirectly via MAGE-A proteins [62,63]. Notably, the Xist lncRNA has been shown to interact with HDAC3 through the SHARP transcriptional repressor to silence transcription [65]. Taken together, these results suggest that a protein complex containing p53, *LINC01021*, and HDAC3 might be involved in the regulation of a subset of p53 targets. Interestingly, the inhibition of HDAC3 by either small molecule inhibitors or siRNAs has been reported to result in increased chemo-sensitivity [66,67,68], suggesting that the increased chemo-sensitivity after the loss of *LINC01021* observed by others and us [24,25] may be due to the abrogation of complexes involving *LINC01021* and HDAC3. Interestingly, we identified RBBP4 as a potential interaction partner of *LINC01021* by RAP-MS. RBBP4 is a subunit of several HDAC1/2-containing protein complexes as well as the DREAM complex, which has been implicated in the indirect repression of gene expression by p53 via binding to E2F or cell cycle genes homology region (CHR) DNA binding sites [69]. However, the repression of DREAM target genes was not significantly different in cells with a loss of *LINC01021*-inducibility by p53 compared to wild-type cells, arguing against a potential role of *LINC01021* for DREAM complex function.

The gene encoding the RNA-binding protein and splicing regulator ZMAT3 is a direct p53 target and mediates the regulation of pre-mRNA splicing by p53 [55,56]. Moreover, several lncRNAs have been reported to be involved in the regulation of pre-mRNA splicing by various direct and indirect mechanisms [57]. Interestingly, we identified a number of p53-regulated transcript isoform switches that were affected by the loss of concomitant *LINC01021* induction and had been previously reported to generate multiple protein isoforms, e.g., of STOML2, ARHGAP12, HSF2, and the LYN kinase. While the role of the different STOML2, ARHGAP2, and HSF2 protein isoforms is not fully understood, the ratio of LYNA and LYNB protein isoforms has been reported to have an effect on breast cancer patient survival [58], at least in part because the LYNA and LYNB isoforms confer different migratory and invasive properties to breast cancer cells. In the future, further analyses are therefore warranted to determine the functional relevance of these protein isoforms in the context of p53 function and tumor suppression. Using RAP-MS, we identified several RNA binding proteins associated with *LINC01021* that are known to be involved in pre-mRNA splicing, such as several heterogeneous nuclear ribonucleoproteins (HNRNPs) as well as the SRSF2/3/6 splicing regulators. Notably, both HNRNPs and SRSF proteins have previously been reported to associate with lncRNAs. For example, the lncRNA *SNHG1* associates with HNRNPC to regulate p53 activity [70]. Moreover, the lncRNA *CYTOR* interacts with HNRNPC to regulate *ZEB1* mRNA stability and thus promotes the epithelial–mesenchymal transition (EMT) [71]. Interestingly, the lncRNA CRNDE associates with SRSF6, thereby reducing its stability, which results in altered alternative splicing of the *PICALM* mRNA and attenuated chemoresistance [72]. The association of *LINC01021* with several proteins involved in pre-mRNA splicing could potentially contribute to the effect of *LINC01021* on p53-regulated RNA isoform switches. However, the exact mechanism of *LINC01021*-mediated alternative splicing has to be determined in the future.

Taken together, our study provides a comprehensive framework and resource for the further analyses of *LINC01021* function downstream of p53. In addition, the observations and conclusions described above may provide valuable leads for further in-detail studies of the p53/*LINC01021* axis in tumor suppression.

## 5. Conclusions

Taken together, our results demonstrate a negative regulatory effect of *LINC01021* on a subset of p53-induced genes. Moreover, the loss of *LINC01021* induction by p53 results in altered p53-regulated transcript isoform expression for a select number of mRNAs, presumably by affecting alternative splicing events. *LINC01021* thus serves as a factor that modulates the transcriptional program regulated by p53.

## Data Availability

The data that support the findings of this study are available from the corresponding author upon reasonable request. The mass spectrometry proteomics data are available via ProteomeXchange with identifier PXD050892. The RNA expression profiling data obtained in this study were deposited on the Gene Expression Omnibus website (accession no. GSE162710).

## Supplementary Materials

The following supporting information can be downloaded at: https://www.mdpi.com/article/10.3390/cancers16091639/s1, Figure S1: Original, uncropped Western Blot images. Table S1: Guide RNA sequences used for CRISPR/Cas9-mediated deletions within the *LINC01021* promoter. Table S2: Oligonucleotides used for cloning and genotyping. Table S3: Oligonucleotides used for qRT-PCR. Table S4: List of significantly regulated mRNAs. Table S5: List of differentially regulated mRNAs. Table S6: List of differentially regulated transcript isoforms.

## Abbreviations

BDS	binding site
cDNA	complementary DNA
CRC	colorectal cancer
CRISPR	Clustered Regularly Interspaced Short Palindromic Repeats
DOX	doxycycline
EMT	epithelial–mesenchymal transition
FACS	fluorescence-activated cell sorting
GSEA	gene set enrichment analysis
KO	knockout
Lnc	long noncoding
qRT-PCR	quantitative reverse transcription polymerase chain reaction
siRNA	Small interfering RNA
sgRNA	single guide RNA
