# Supplementary material for: LINC01021 Attenuates Expression and Affects Alternative Splicing of a Subset of p53-Regulated Genes
Source: Cancers (Basel). 2024 Apr 24;16(9):1639. doi: 10.3390/cancers16091639 (PMC11083319; doi:10.3390/cancers16091639)
Supplement: Supplementary file 1 [file cancers-16-01639-s001.zip › cancers-2957022-supplementary.pdf]

LINC01021 attenuates expression and affects alternative splicing of a subset of p53-regulated genes

Markus Kaller[1], Ignasi Forné[2], Axel Imhof[2] and Heiko Hermeking[1,3,4*]

Supplementary Information

Supplementary Figures

Figure S1: Original, uncropped Western Blot images.

Supplementary Tables

Table S1: Guide RNA sequences used for CRISPR/Cas9-mediated deletions within the *LINC01021* promoter.

Table S2: Oligonucleotides used for cloning and genotyping.

Table S3: Oligonucleotides used for qRT-PCR.

Table S4: List of significantly regulated mRNAs.

This table includes the list of mRNAs significantly regulated (fold change >1.5) regulated in *LINC01021* wild-type cells and/or KO cells. Log_2 fold changes and FDR-adjusted p-values (padj) in *LINC01021* wt and KO cells as determined by DESEQ2 are provided.

Table S5: List of differentially regulated mRNAs.

This table includes the list of mRNAs with significant differences in regulation between *LINC01021* wild-type cells and KO cells. Log_2 fold changes and FDR-adjusted p-values (padj) in *LINC01021* wild-type cells and KO cells as determined by DESEQ2 are provided.

Table S6: List of differentially regulated transcript isoforms.

This table includes the list of transcript isoforms differentially regulated between *LINC01021* wild-type cells and KO cells. Log_2 fold changes and FDR-adjusted p-values (padj) in *LINC01021* wild-type cells and KO cells as determined by DESEQ2 are provided.

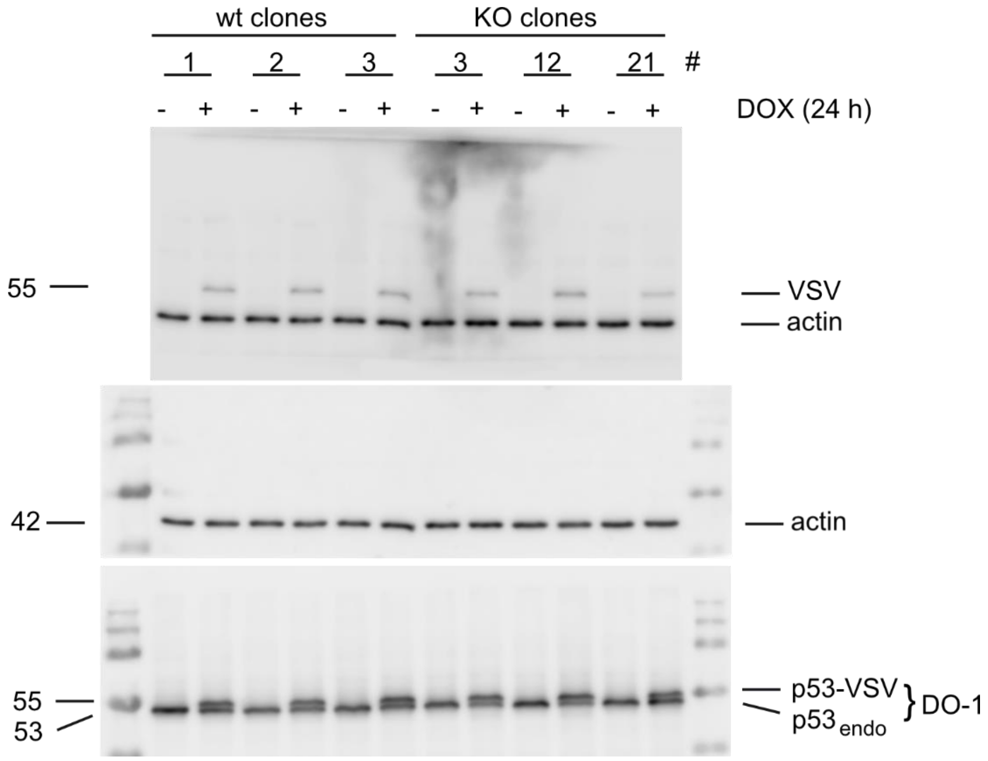

Figure S1: Original, uncropped Western blot images.

Table S1: **Guide RNA sequences used for CRISPR/Cas9-mediated deletions within the *LINC01021* promoter**

guide RNA name	sequence 5´- 3´	Genomic coordinates (hg38)
LINC01021 guide 1	AGGGGTCCCTAGAGAATTTC	chr5:27472086-27472105
LINC01021 guide 2	GAGGAATTCATGCCTTGCAA	chr5:27472235-27472254

Table S2: **Oligonucleotides used for cloning and genotyping**

Primer name	sequence 5´- 3´
LINC01021 screen *Fwd*	AAGGAAACCTCAAAACGTGAAGGAC
LINC01021 screen *Rev*	CATTTCTCAAGCCCCGTGTTTGGG
LINC01021 cloning *Fwd*	TTTACTAGTCTGGGAATCAATGTGTGAGGT
LINC01021 cloning *Rev*	TTTGAGCTCTTAAGCCCCTCATTCGTTATATTTT

Table S3: **Oligonucleotides used for qPCR**

Primer name	sequence 5´- 3´
LINC01021 Fwd	TGTTTTCCTTTTTGCCCAAT
LINC01021 Rev	CGCCTGGTAAAACAACCAGT
EZH2 Fwd	CGCTTTTCTGTAGGCGATGT
EZH2 Rev	TGGGTGTTGCATGAAAAGAA
HDAC3 Fwd	GGCCTATTTCTACGACCCCG
HDAC3 Rev	TGGTATGGCTTGAAGACGATCA
BTG1 Fwd	CAAGGGATCGGGTTACCGTTGT
BTG1 Rev	AGCCATCCTCTCCAATTCTGTAGG
FAS Fwd	ATGGTGTCAATGAAGCCAAA
FAS Rev	TCCATGAAGTTGATGCCAAT
PMAIP1/NOXA Fwd	GGAGATGCCTGGGAAGAAG
PMAIP1/NOXA Rev	CCTGAGTTGAGTAGCACACTCG
DKK1 Fwd	CAGGCGTGCAAATCTGTCT
DKK1 Rev	AATGATTTTGATCAGAAGACACACATA
TP53 Fwd	CCTCAGCATCTTATCCGAGTGG
TP53 Rev	TGGATGGTGGTACAGTCAGAGC

Table S4: **List of significantly regulated mRNAs**

**gene symbol**	**log2FoldChange (+DOX vs -DOX) (*LINC01021* wt)**	**padj**	**gene symbol**	**log2FoldChange (+DOX vs -DOX) (*LINC01021* KO)**	**padj**
GDF15	5,870	2,54E-53	*GDF15*	6,253	1,66E-54
INPP5D	5,336	8,19E-60	*COL17A1*	6,224	6,89E-40
RP3-510D11.2	4,509	3,36E-23	*INPP5D*	6,021	3,28E-57
COL17A1	4,232	6,14E-27	*TENM4*	5,897	4,86E-17
TENM4	4,161	1,95E-09	*KRTAP2-3*	5,306	2,52E-08
LAMC3	3,995	3,77E-18	*SORCS2*	4,628	8,21E-12
KRTAP2-3	3,584	0,000853193	*GJB4*	4,607	4,47E-10
CEL	3,578	8,22E-21	*RP3-510D11.2*	4,467	4,87E-26
NGFR	3,461	1,50E-45	*NUPR1*	4,243	1,20E-07
NUPR1	3,304	0,000477478	*ROBO4*	4,132	1,77E-08
MDM2	3,256	6,32E-53	*LAMC3*	4,017	2,71E-21
SORCS2	3,209	9,31E-06	*PVRL4*	3,973	2,30E-20
ACTA2	3,045	7,91E-17	*TREM2*	3,814	2,74E-11
CDKN1A	2,968	1,69E-63	*CEL*	3,738	6,11E-24
TP53I3	2,878	9,21E-09	*ACTA2*	3,687	1,72E-24
ROBO4	2,848	0,000456403	*TP53I3*	3,674	1,17E-14
TNFSF9	2,787	1,69E-21	*MDM2*	3,551	2,49E-63
PVRL4	2,708	1,61E-10	*LYNX1*	3,470	7,10E-16
TREM2	2,657	1,81E-06	*ZNF114*	3,432	1,43E-08
ZNF114	2,641	2,10E-05	*PRODH*	3,298	5,26E-08
LYNX1	2,638	7,77E-10	*PERM1*	3,295	0,000237434
MYH16	2,629	2,48E-05	*KRTAP3-1*	3,286	7,40E-22
ZMAT3	2,615	3,46E-47	*SAA2*	3,285	1,59E-06
RRM2B	2,546	1,05E-39	*DQX1*	3,267	6,29E-11
CD70	2,535	1,02E-10	*UNC13A*	3,213	8,82E-11
GJB4	2,527	1,84E-06	*NGFR*	3,200	2,84E-39
RP5-1063M23.1	2,413	0,000134606	*SCN3B*	3,188	7,04E-07
FLG-AS1	2,342	6,23E-26	*PXDN*	3,130	0,011718444
HSD3BP5	2,335	0,001419719	*DKK1*	3,120	1,42E-27
LAPTM5	2,330	8,58E-07	*CFAP74*	3,109	1,18E-10
CAPN8	2,318	1,81E-19	*CYP3A7-CYP3A51P*	3,051	2,95E-05
MVP	2,296	3,39E-14	*MYH16*	3,049	1,39E-08
EPHB1	2,279	0,015771702	*HECW2*	3,043	2,92E-05
SLC6A12	2,266	6,36E-05	*CDKN1A*	3,035	4,13E-67
EHF	2,258	1,01E-06	*COL5A1*	3,006	1,76E-11
LINC00475	2,250	0,000217047	*SERPINB5*	3,002	2,45E-17

CYP3A7-CYP3A51P	2,238	0,007431204	*FLG-AS1*	2,989	4,14E-43
VWCE	2,234	1,03E-08	*CRYAB*	2,966	0,003214146
PIK3IP1	2,233	0,000234631	*EHF*	2,930	1,58E-10
COL5A1	2,149	3,71E-06	*RP11-83N9.5*	2,869	1,56E-06
ATG9B	2,139	0,043400808	*HSD3BP5*	2,826	2,95E-05
APOBEC3H	2,138	2,47E-08	*IL1R2*	2,770	8,73E-05
G0S2	2,135	0,036467698	*AK5*	2,756	8,64E-10
KRTAP3-1	2,090	2,25E-08	*PSG1*	2,731	9,80E-05
CD2	2,086	0,000686289	*PSG9*	2,727	2,69E-06
LCP1	2,083	0,005880955	*CD70*	2,724	1,07E-12
KITLG	2,080	3,03E-10	*CSF2*	2,723	2,31E-08
DQX1	2,077	0,00039358	*TNFSF9*	2,715	1,18E-20
TMEM229B	2,060	3,87E-09	*ZMAT3*	2,692	1,89E-50
SAA2	2,057	0,010276926	*RRM2B*	2,679	3,79E-44
BTBD19	2,052	4,35E-07	*ATG9B*	2,667	0,000689064
HIST1H2BD	2,046	2,23E-10	*CAPN8*	2,663	3,40E-27
KLHDC7A	2,035	5,43E-05	*TG*	2,653	1,69E-07
CYFIP2	2,033	2,41E-13	*FAM49A*	2,644	4,85E-06
FDXR	2,032	4,11E-47	*CST5*	2,633	1,02E-05
TG	2,031	0,000153805	*CTGF*	2,624	9,13E-19
PLXNA4	2,015	0,023215398	*DUSP13*	2,612	0,000240468
CFAP74	2,011	0,000189949	*LINC01411*	2,607	1,96E-11
IL1R2	2,006	0,010907805	*LAPTM5*	2,599	1,41E-08
KIAA1211L	2,003	0,020247049	*BTBD19*	2,576	1,97E-10
EBI3	1,983	0,003809907	*SAA2-SAA4*	2,551	3,52E-09
BTG2	1,975	1,59E-21	*MVP*	2,548	4,12E-18
RPS27L	1,958	1,35E-54	*IGFBP7*	2,546	4,46E-07
RP11-83N9.5	1,956	0,001199818	*KLHDC7A*	2,523	9,29E-08
CARNS1	1,953	9,62E-16	*SAA1*	2,498	3,05E-06
GALNT5	1,952	0,000178909	*C3*	2,477	9,06E-18
ADAMTS7	1,951	2,95E-16	*TSPAN1*	2,477	9,23E-15
SCN3B	1,932	0,009251941	*LINC01060*	2,443	1,93E-07
PSG9	1,931	0,002669505	*VWCE*	2,418	1,99E-11
PRODH	1,930	0,006737845	*KIAA1324*	2,414	6,27E-15
C3	1,905	3,59E-10	*AC006262.5*	2,409	7,72E-09
CSF2	1,898	0,000324905	*DDX47*	2,401	4,12E-07
CTGF	1,896	3,12E-09	*CYP3A5*	2,390	6,99E-26
AC006262.5	1,881	2,28E-05	*RP11-529A4.4*	2,385	2,66E-07
SERPINB5	1,878	4,47E-07	*CCL7*	2,359	0,004373056
FILIP1	1,875	0,006881005	*CLIP4*	2,347	6,08E-05
SMIM10L2A	1,853	0,000549435	*IL2RG*	2,321	0,016201499
DRAM1	1,848	5,58E-24	*HIST1H2AC*	2,319	3,26E-12
TSPAN1	1,847	1,45E-07	*KITLG*	2,316	1,54E-12

GPD1	1,845	0,000219269	*KIAA1211L*	2,307	0,001193968
DKK1	1,833	2,09E-09	*KRT6A*	2,300	3,28E-09
ANK1	1,832	3,56E-12	*PLTP*	2,293	5,34E-08
IGFBP7	1,826	0,001083325	*VSIG1*	2,285	0,013868047
CST5	1,813	0,00863789	*G0S2*	2,284	0,010370752
PSG1	1,805	0,027965132	*RP5-1063M23.1*	2,262	3,55E-05
SAA2-SAA4	1,769	0,000178909	*FDXR*	2,262	2,43E-59
LINC01060	1,753	0,000499286	*RPS27L*	2,254	4,20E-73
LCTL	1,750	1,62E-05	*CD2*	2,250	4,58E-05
HIST1H2AC	1,744	6,78E-07	*CYFIP2*	2,211	2,05E-16
NEURL3	1,698	0,001264901	*MAEL*	2,203	0,006031082
AC128709.2	1,686	0,038900659	*HIST1H2BD*	2,202	1,09E-12
PADI1	1,680	6,10E-16	*NEURL3*	2,200	2,10E-06
SAA1	1,671	0,00672933	*BTG2*	2,195	5,19E-27
CYP3A5	1,666	1,52E-12	*PSG7*	2,186	0,007793439
SHC4	1,662	0,020027392	*CD82*	2,178	0,000182474
LINC01411	1,644	6,06E-05	*ESM1*	2,156	0,021813415
KIAA1324	1,642	5,77E-07	*FAM13C*	2,150	2,33E-08
CD22	1,637	0,002907025	*AOX1*	2,140	4,90E-05
KCNN4	1,626	0,017653998	*APOBEC3H*	2,122	5,23E-09
NT5DC4	1,625	0,02336931	*CPA4*	2,103	0,00019503
HIST1H3H	1,621	4,86E-08	*DRAM1*	2,096	3,87E-32
UNC13A	1,599	0,005656629	*LINC00475*	2,081	0,000325652
FAT1	1,590	3,77E-17	*GALNT5*	2,070	5,62E-06
C22orf23	1,569	6,03E-22	*ABAT*	2,038	0,002246295
bP-21264C1.2	1,538	0,005951899	*ANK1*	2,010	1,38E-14
LINC01573	1,494	0,007102901	*ARHGAP9*	1,999	0,022429875
ACHE	1,485	0,002446025	*SUGCT*	1,996	2,88E-13
RP11-1094H24.4	1,484	0,027872854	*KISS1*	1,991	0,006416524
PHLDA3	1,479	0,000494057	*LINC01405*	1,991	0,000105095
HES2	1,477	3,76E-06	*ABCA1*	1,988	0,000265432
CMBL	1,467	2,06E-19	*PLA2G4C*	1,981	5,06E-06
BAX	1,466	2,63E-39	*KANK3*	1,980	0,001476755
PIFO	1,462	0,009584382	*NABP1*	1,959	1,39E-13
AHNAK2	1,451	2,56E-09	*AC128709.2*	1,959	0,006696939

FAM13C	1,439	0,000921017	*FLJ31356*	1,956	0,034190532
RSPH1	1,437	0,018723084	*HAVCR2*	1,953	0,006254164
SESN1	1,437	6,30E-14	*ITIH5*	1,951	0,000447057
CD82	1,416	0,046480306	*RP11-87N24.3*	1,939	0,017482685
DYRK1B	1,413	4,84E-10	*HEPHL1*	1,928	2,71E-05
LINC00106	1,412	0,01574436	*EBI3*	1,922	0,001278342
HIST1H2BN	1,412	0,000338612	*SMIM10L2A*	1,917	0,000158719
ITGB2	1,406	0,016255473	*HIST1H3H*	1,915	8,17E-12
GJB3	1,406	0,00037985	*GJB3*	1,907	7,75E-08
ACER2	1,392	0,003797709	*LCTL*	1,905	5,33E-07
NOS3	1,392	0,017944974	*CLSTN2*	1,892	1,49E-11
PLTP	1,388	0,004750381	*TENM2*	1,890	0,021102172
ABCA1	1,384	0,034529521	*KRT81*	1,886	0,00011425
RP11-2C24.4	1,380	0,013733914	*PIFO*	1,883	0,000243815
RP5-1198O20.4	1,377	0,027813013	*EPHB1*	1,882	0,02650933
CYSRT1	1,356	0,000740621	*ACER2*	1,874	7,62E-06
PLB1	1,354	0,036342857	*MOB3B*	1,873	0,008223755
TSPEAR	1,346	0,030703538	*RP11-618G20.1*	1,857	0,027666077
RP3-395M20.8	1,336	0,001676397	*ZFHX2*	1,853	0,004118112
RP11-529A4.4	1,322	0,023168324	*CMBL*	1,847	4,32E-32
SCARF1	1,321	0,012421322	*CD24*	1,843	7,75E-08
TP53	1,320	7,14E-10	*ADRB2*	1,837	0,00477735
ANKRD24	1,318	0,00162803	*SLC30A10*	1,824	0,007074597
SUGCT	1,309	9,86E-06	*KRT3*	1,819	0,023531693
YPEL2	1,298	0,00017249	*TP53*	1,811	7,64E-19
AP006621.8	1,296	0,032934309	*TMEM229B*	1,787	9,71E-08
SLC5A3	1,284	1,28E-07	*BIRC3*	1,779	3,61E-08
RP11-231C14.4	1,279	0,024694829	*ADAMTS7*	1,773	1,05E-14
MYL5	1,277	0,00010232	*SLC6A12*	1,763	0,009249002
SMG1P6	1,275	0,029807656	*TMEM40*	1,748	0,001100389
DCUN1D3	1,252	7,03E-05	*ANKRD34A*	1,747	0,000285306
CLSTN2	1,249	3,73E-05	*CARNS1*	1,720	1,42E-13
MICALL1	1,245	5,79E-28	*CCL2*	1,698	2,23E-06

ASTN2	1,243	1,59E-12	*PIK3IP1*	1,695	0,002914097
LINC01405	1,241	0,040502938	*RP11-1094H24.4*	1,694	0,001935214
SLFN5	1,234	2,45E-07	*PLB1*	1,693	0,002198906
TMC4	1,229	0,041489715	*ANKRD2*	1,682	0,010212245
LOXL1	1,228	5,02E-06	*NT5DC4*	1,681	0,006141125
FUCA1	1,220	6,67E-14	*PADI1*	1,667	4,82E-16
CATSPERG	1,217	0,011386024	*FAT1*	1,660	4,44E-19
ORAI3	1,214	9,45E-06	*ACHE*	1,658	0,000161277
MXD4	1,197	1,72E-12	*KCNN4*	1,655	0,007944022
PLA2G4C	1,195	0,01840839	*HSPB8*	1,651	0,001509876
NTF4	1,195	0,004232547	*AHNAK2*	1,650	2,32E-12
PTPRU	1,187	1,05E-14	*SESN1*	1,650	6,61E-19
NABP1	1,184	4,57E-05	*TGFB2*	1,650	0,000808911
HEPHL1	1,177	0,034207909	*HIST1H2AI*	1,650	0,038570705
KRT6A	1,176	0,012389288	*CYSRT1*	1,641	8,67E-06
ABHD14A-ACY1	1,164	0,042955266	*RP11-407N17.3*	1,632	0,004088068
TMEM150A	1,159	5,11E-09	*HES2*	1,624	1,08E-07
PLK3	1,154	1,26E-15	*HIST1H4I*	1,602	6,30E-06
TIMP3	1,146	5,32E-08	*C22orf23*	1,601	5,70E-24
PRDM1	1,132	1,93E-05	*SCARF1*	1,598	0,000606932
RP5-1086K13.1	1,115	0,038580231	*CTD-3074O7.11*	1,598	0,004423843
FAM212B	1,113	4,56E-10	*AFF3*	1,596	0,013586991
GM2A	1,109	1,71E-06	*ALPK2*	1,595	0,018638161
DDB2	1,097	8,81E-13	*WNT9A*	1,587	3,74E-05
TIGAR	1,089	1,41E-08	*ARHGAP30*	1,586	0,033022005
LOXL4	1,088	1,44E-07	*TIGAR*	1,581	2,04E-18
RP11-513I15.6	1,087	0,0017101	*CDH13*	1,567	8,76E-43
GAS7	1,083	0,002830641	*NT5E*	1,563	0,009949786
APOE	1,080	8,33E-06	*TMC4*	1,560	0,002093191
TRPV4	1,078	0,000660387	*AP3B2*	1,558	0,014835808
SULF2	1,075	4,65E-06	*RP3-395M20.8*	1,557	3,28E-05
PLCXD2	1,073	0,001721963	*PRDM1*	1,554	3,37E-10
XDH	1,072	0,034047129	*GM2A*	1,543	1,19E-12

HIST2H2BE	1,072	5,49E-05	*FAM20C*	1,536	0,0374829 32
CDH13	1,071	1,66E-19	*TMEM150A*	1,530	3,21E-16
PRRG2	1,056	0,030148551	*PDCD4-AS1*	1,519	0,0169959 78
PORCN	1,047	3,24E-06	*ANXA8L1*	1,517	0,0264097 84
RLBP1	1,047	0,02633084	*CACNA2D3*	1,515	0,0229565 34
SYTL1	1,036	6,82E-12	*NOS3*	1,515	0,0040419 1
NEAT1	1,034	0,039643784	*SLFN5*	1,513	3,08E-11
C22orf29	1,028	0,033123544	*TTC29*	1,512	0,0176726 56
PARD6G	1,021	2,36E-06	*ASB2*	1,504	0,0060903 55
CCL2	1,016	0,02185244	*NTF4*	1,503	3,29E-05
ITGB4	1,015	9,66E-05	*AKAP12*	1,503	0,0486608 18
GADD45A	1,010	5,62E-10	*PHLDA3*	1,493	0,0002278 59
EME2	1,004	4,31E-06	*XDH*	1,491	0,0002279 3
RPL23AP82	1,003	9,40E-14	*EDN1*	1,491	6,29E-08
PGPEP1	1,003	7,53E-25	*SHC4*	1,490	0,0343853 63
IGDCC4	1,003	0,000856945	*ASTN2*	1,484	1,93E-18
HIST1H2AG	0,998	0,034696345	*URGCP-MRPS24*	1,484	0,0002017 84
PAPL	0,996	0,005689613	*FUCA1*	1,482	3,98E-21
SPTBN5	0,990	3,77E-07	*DOCK8*	1,473	3,22E-06
RHOD	0,990	1,04E-05	*PORCN*	1,471	2,19E-12
PTPRE	0,989	2,29E-06	*ZNF793*	1,468	0,0456030 87
SPNS2	0,986	0,000173236	*PAPL*	1,466	2,79E-06
LCAT	0,981	4,87E-05	*CD22*	1,450	0,0052260 89
CSMD2	0,980	0,011688246	*GADD45A*	1,444	7,07E-21
ZNF425	0,971	0,020145296	*SERPINE1*	1,438	0,0068979 8
BBC3	0,969	1,01E-07	*ITGB2*	1,434	0,0070291 52
NPAS2	0,955	0,024679561	*IL16*	1,432	0,0318452 11
SESN2	0,955	7,01E-06	*AC006369.2*	1,431	0,0343991 57
HLA-DQB1	0,952	0,010519835	*ZNF554*	1,429	7,67E-07
CD163L1	0,943	0,000219269	*CST4*	1,428	0,0485635 83
KCTD21	0,943	0,000635933	*GAMT*	1,423	0,0249243 34
DOCK8	0,942	0,011876705	*RP11-361L15.4*	1,418	0,0475652 34

SEMA3B	0,941	5,66E-05	*ITGB8*	1,413	4,80E-07
C15orf52	0,940	2,68E-18	*CD163L1*	1,412	7,80E-10
NLRP1	0,937	1,17E-06	*TINCR*	1,409	0,014519015
ITGA3	0,935	8,83E-24	*SQSTM1*	1,405	2,31E-15
C1QTNF1-AS1	0,934	0,000669094	*PHYHIP*	1,401	0,004658038
MXRA8	0,931	0,003010938	*DYRK1B*	1,388	2,88E-10
MYH15	0,923	0,002309071	*MICALL1*	1,388	1,11E-35
S100A2	0,922	0,000331526	*DRAXIN*	1,384	0,001060115
RGS16	0,921	4,22E-06	*ATP8A2*	1,381	0,033456782
FCGBP	0,915	0,002693233	*PPP1R1C*	1,381	0,022334439
CDC42EP3	0,912	9,82E-05	*MTUS2*	1,378	0,035692225
TRANK1	0,912	6,85E-06	*WNT7A*	1,374	0,001408891
HIST3H2A	0,908	0,000426478	*GATM*	1,366	0,021528892
PHPT1	0,908	6,36E-05	*HPGD*	1,361	1,11E-07
CTSB	0,905	2,48E-08	*CSMD2*	1,354	7,36E-05
CDC42BPG	0,904	0,000372505	*CNTNAP3*	1,351	0,027841712
S100A3	0,901	0,017027757	*AATBC*	1,341	0,00404191
ZNF469	0,900	0,033022532	*RP11-299G20.2*	1,341	0,023323469
SQSTM1	0,900	2,36E-06	*ZNF658*	1,340	0,000166036
KRT86	0,897	0,012355239	*DDX60L*	1,331	5,71E-06
AEN	0,896	1,36E-08	*MYL5*	1,330	1,35E-05
RAB3B	0,891	1,49E-05	*KRT23*	1,329	0,029940134
CCND1	0,884	0,00020243	*NPAS2*	1,323	0,000208337
DCP1B	0,879	3,29E-13	*MT2A*	1,316	3,68E-07
EPS8L2	0,876	7,86E-05	*PMAIP1*	1,316	6,72E-18
MT1X	0,876	0,031194271	*FLJ22447*	1,312	0,000577163
BBS12	0,871	0,00186278	*IRAK2*	1,311	0,021334614
SLC12A4	0,866	2,98E-07	*IL7R*	1,311	0,041217082
MT2A	0,866	0,003296809	*PTPRE*	1,310	3,08E-11
PADI3	0,863	0,000200922	*SOCS2*	1,306	1,28E-08
FAM210B	0,862	1,10E-06	*CNTNAP3B*	1,306	0,033444262
DEPTOR	0,862	0,036842853	*TM4SF19*	1,303	0,004402553
MMP14	0,860	2,21E-10	*BAX*	1,301	1,76E-31
EDN1	0,859	0,008486943	*FMN1*	1,293	2,00E-05

FBXO2	0,859	0,011308263	*LOXL1*	1,290	5,31E-07
PLXNB3	0,855	0,001940657	*CTSB*	1,284	7,99E-17
ASCC3	0,855	3,25E-06	*MT1E*	1,281	4,31E-08
SLC35F6	0,855	0,022272032	*HIST1H2BK*	1,277	0,0278182 14
KCTD11	0,854	2,67E-07	*TIMP3*	1,275	3,83E-10
ANKRA2	0,847	7,44E-07	*SLC52A1*	1,274	0,0019986 99
ERC2	0,847	0,025865121	*HIST1H2AG*	1,272	0,0013925 52
PLEKHG1	0,844	0,001358275	*AC009237.8*	1,269	0,0198761 77
TMEM86A	0,841	0,015123769	*CAV1*	1,266	9,45E-09
ELFN2_1	0,838	0,036342857	*C1QTNF1-AS1*	1,266	1,45E-07
TRIM29	0,835	0,022137858	*RSPH1*	1,264	0,0198761 77
ALOX12B	0,835	0,000398764	*GULP1*	1,259	2,74E-11
ZNF556	0,831	0,001817569	*NLRP1*	1,259	3,96E-12
LRP1	0,827	0,002869293	*GAS7*	1,254	0,0001788 22
CACNG8	0,814	0,001355353	*DDB2*	1,251	2,84E-17
TM7SF2	0,811	0,022140802	*TNFRSF9*	1,250	0,0072126 15
THSD4	0,810	4,62E-06	*PTPRU*	1,246	8,98E-17
ZNF554	0,809	0,024097063	*PLK3*	1,242	6,61E-19
FMN1	0,808	0,025010358	*SNAI2*	1,238	0,0002857 5
CENPBD1	0,806	0,046380413	*ZNF497*	1,231	0,0134302 04
CTD-2267D19.3	0,805	4,41E-05	*KRT86*	1,231	0,0001102 1
RP11-380J14.1	0,803	0,006949856	*SERPINA1*	1,229	1,59E-06
ANKRD1	0,787	0,000157212	*SLC44A5*	1,228	0,0003717 35
LAMA3	0,785	0,043400516	*LINC01573*	1,228	0,0119043 18
MIR22HG	0,784	0,003509934	*S100A2*	1,225	2,28E-07
SOCS2	0,780	0,004234742	*MYH15*	1,213	8,72E-06
PLXNB2	0,778	7,60E-11	*RNF144B*	1,213	0,0012107 67
XPC	0,772	6,82E-12	*FAM210B*	1,213	2,88E-13
CCND2	0,770	1,47E-05	*THNSL1*	1,213	0,0040880 68
DISP2	0,770	2,92E-06	*NLRP3*	1,208	0,0001613 44
CACNA2D4	0,768	0,013248598	*PTPRZ1*	1,207	0,0434332 6
SLC27A1	0,767	0,019432103	*TRANK1*	1,205	3,44E-10
HGSNAT	0,767	0,000295421	*SPTBN5*	1,203	3,00E-11
SLC7A7	0,765	0,033851608	*MXD4*	1,201	3,55E-13
IDUA	0,764	0,007755402	*HIST2H2BE*	1,200	1,26E-06

DDX60L	0,763	0,043400516	*RNF128*	1,196	0,0212101<br>62
ATF3	0,763	0,002725988	*CTD-3088G3.8*	1,195	0,0436594<br>79
KSR2	0,758	0,006897293	*SDCBP2*	1,192	0,0018982<br>89
CCNE1	0,750	4,87E-08	*APOE*	1,185	2,06E-07
LACC1	0,746	1,90E-05	*TM4SF1*	1,183	0,0002654<br>32
PIDD1	0,746	0,014527953	*PLAT*	1,182	0,0238388<br>07
RINL	0,741	0,002093119	*TRAF1*	1,180	2,55E-05
ARVCF	0,740	0,018145963	*GPD1*	1,180	0,0174892<br>45
BCL2L1	0,736	8,74E-09	*ABHD15*	1,179	0,0420144<br>36
UFSP1	0,736	0,001230776	*ALDH1A3*	1,177	0,0450175<br>47
GULP1	0,736	0,000614096	*YPEL2*	1,173	0,0004636<br>04
TEP1	0,734	1,70E-07	*CEP170P1*	1,171	0,0135869<br>91
KRT8	0,733	9,98E-05	*TSC22D1*	1,170	1,76E-16
PLEC	0,733	0,010594997	*FAS*	1,166	3,09E-15
SERPINA1	0,729	0,017729875	*SERPINE2*	1,165	5,56E-18
OSBPL3	0,725	1,65E-08	*RHOD*	1,159	4,69E-08
ZNF79	0,725	0,011207644	*KCNJ4*	1,158	0,0034715<br>52
TRNP1	0,724	9,41E-10	*RP11-513I15.6*	1,153	0,0003616<br>69
PLK2	0,721	1,31E-07	*ZNF20*	1,145	0,0226618<br>14
FEZ1	0,720	0,012485416	*SULF2*	1,141	4,46E-07
MT1E	0,720	0,009613991	*ORAI3*	1,135	2,11E-05
PLCD3	0,720	3,28E-12	*ANKRD24*	1,135	0,0041187<br>26
SLC48A1	0,720	0,001207203	*RPL23AP82*	1,133	5,12E-18
SUSD6	0,718	4,32E-07	*RAB3B*	1,132	4,71E-09
NINJ1	0,716	9,72E-05	*HGSNAT*	1,127	2,23E-09
TP53TG1	0,713	0,037710452	*SLC16A4*	1,124	0,0311892<br>58
RIN1	0,712	0,004521785	*PLAU*	1,117	1,36E-07
CALCOCO1	0,710	2,87E-06	*ZNF425*	1,111	0,0013242<br>38
SGK223	0,708	1,86E-06	*RLBP1*	1,103	0,0077644<br>96
FBXO22	0,703	6,30E-11	*RP11-46A10.5*	1,103	0,0481957<br>28
MTFR1L	0,701	6,23E-06	*PLEKHG1*	1,100	2,65E-06
C5orf66	0,691	0,006082268	*ITGB4*	1,097	9,54E-06
DOCK9	0,688	0,022768151	*SGK1*	1,096	0,0318452<br>11
ZNF446	0,687	0,010416389	*ASCC3*	1,095	2,83E-10

APLP1	0,686	0,005695669	*SLC26A4*	1,091	0,005366908
RNF19A	0,681	0,035674537	*ETS1*	1,090	3,63E-08
TNFRSF14	0,680	0,003492711	*ANKRA2*	1,088	1,90E-11
ACTN1	0,680	8,66E-10	*FEZ1*	1,087	2,06E-05
PEAR1	0,678	0,002003462	*KRT15*	1,085	0,004049652
ADAM19	0,675	9,51E-06	*PTAFR*	1,083	0,017606881
SHC1	0,673	1,33E-06	*TRIM29*	1,083	0,000606932
PSTPIP2	0,670	5,59E-06	*RP11-136O12.2*	1,081	0,007237088
TSC22D1	0,666	1,89E-05	*SLC26A4-AS1*	1,080	0,008376074
ZFP36L1	0,663	0,023849625	*PARD6G*	1,080	3,15E-08
MYEOV	0,660	0,025979604	*DCBLD2*	1,079	0,046306222
WNT5A	0,658	0,000423992	*PLK2*	1,079	4,86E-17
FAS	0,656	7,35E-05	*AC011747.6*	1,079	0,022463334
NADSYN1	0,653	0,012787949	*PLEK*	1,073	0,006664258
POLD4	0,651	0,007049374	*FAM134B*	1,072	0,011784098
PRR5	0,646	0,048354823	*CPEB2*	1,072	0,012447201
SLC20A1	0,644	3,74E-05	*PTP4A1*	1,070	1,11E-10
APOBEC3C	0,642	5,02E-06	*TRPM6*	1,070	0,023809373
PRKCZ	0,640	0,000376606	*MIR22HG*	1,068	4,81E-06
CST7	0,640	0,001351478	*PRAP1*	1,067	0,000298615
COL7A1	0,640	0,02799192	*GCK*	1,067	0,004926908
ADAM23	0,639	0,005601723	*GRIN2C*	1,065	0,02610832
PTK6	0,638	0,025351522	*TNFRSF11B*	1,063	0,007346146
ANXA4	0,635	5,32E-06	*TMEM255B*	1,063	0,014835808
PLXNC1	0,633	0,028038916	*ABLIM2*	1,061	0,006032026
STEAP3	0,633	0,008754552	*RP11-2C24.4*	1,061	0,036669463
RHOQ	0,631	0,012744464	*LOXL4*	1,060	1,60E-07
PTP4A1	0,627	0,00082935	*CCND1*	1,059	1,98E-06
MALAT1_1	0,626	0,000850633	*SRGAP3*	1,055	0,008446952
MR1	0,623	0,012000221	*TRIAP1*	1,051	4,72E-08
IP6K2	0,619	0,00011292	*PLXNB3*	1,046	3,12E-05
IRGQ	0,615	0,029815867	*CPM*	1,045	0,041021484

CLN8	0,611	0,000639627	*SYTL1*	1,045	8,62E-13
SARM1	0,610	0,021342611	*PLCXD2*	1,045	0,000606932
KRT5	0,609	6,88E-06	*RDH5*	1,034	0,019748481
BIK	0,606	0,00043297	*TP53TG1*	1,031	0,000188972
KLK6	0,605	0,023415146	*CACNA2D4*	1,029	0,00013766
FHL2	0,604	0,000475272	*DCP1B*	1,029	7,35E-19
DLG1	0,603	0,005765626	*TAGLN*	1,029	0,011578858
LAMB3	0,601	0,011370757	*ATP8B1*	1,027	0,042289038
TNFRSF10B	0,599	7,09E-11	*ADAM23*	1,022	2,20E-07
LAMC2	0,598	0,022064317	*FAM212B*	1,020	1,79E-09
TBC1D2	0,598	0,022421921	*IGDCC4*	1,016	0,000354396
ZNF219	0,595	0,01000707	*RINL*	1,016	1,78E-06
NOL6	0,594	0,022011645	*PUS3*	1,013	4,42E-07
SIDT2	0,594	0,008754552	*SRPX*	1,008	0,004225158
MYOF	0,593	2,77E-06	*PSTPIP2*	1,008	1,68E-13
GPX1	0,592	0,001708878	*DTX4*	1,002	0,008862083
LIF	0,590	0,001764487	*SESN2*	1,002	7,61E-07
MDFI	0,588	0,000401533	*CCNE1*	1,001	4,73E-15
DGKA	0,588	5,44E-07	*C15orf52*	1,001	2,66E-21
FAM102A	0,586	0,000182831	*BATF2*	0,996	0,043016868
ZNF470	0,585	0,000743609	*SLCO1B3*	0,996	0,048990421
PMEPA1	0,583	0,000850633	*PYURF*	0,991	4,38E-06
SLC38A7	0,582	0,001971708	*GAS6-AS2*	0,988	0,002748343
ARL13B	-0,581	0,015807701	*ATF3*	0,988	1,61E-05
CKLF	-0,582	6,76E-05	*SLC16A2*	0,987	4,18E-08
H2AFV	-0,583	1,66E-05	*DEPTOR*	0,984	0,006925246
TOPBP1	-0,583	7,08E-05	*SLC7A7*	0,984	0,001665848
DNMT1	-0,583	0,000882714	*RP11-15J10.1*	0,983	0,009558007
SMC1A	-0,583	4,54E-05	*C5orf66*	0,982	8,74E-06
CDCA7	-0,586	3,29E-06	*ARVCF*	0,982	0,000241341
PDX1	-0,586	0,035478419	*MALAT1_1*	0,979	5,41E-09
SVIP	-0,587	0,000445649	*LACC1*	0,977	1,61E-09
PIM1	-0,588	0,012674126	*BLOC1S2*	0,975	3,11E-09
RNF26	-0,589	1,76E-05	*NR2F1*	0,973	4,58E-08
NASP	-0,589	9,22E-07	*UBR5-AS1*	0,971	0,005151403

DCLRE1B	-0,591	0,000230955	*ANXA4*	0,969	5,54E-14
FBXL16	-0,591	0,043400516	*CST7*	0,968	5,06E-08
FZD1	-0,591	0,024434838	*PGPEP1*	0,962	2,10E-23
FANCM	-0,593	0,005883516	*DLG1*	0,961	3,38E-07
TCTN2	-0,594	0,011276892	*HHAT*	0,956	0,000325652
DBF4	-0,594	1,65E-05	*ANKRD1*	0,951	1,07E-06
RTN4RL2	-0,595	0,048075346	*PROS1*	0,949	0,007926192
PPIH	-0,596	0,000464435	*DLGAP1-AS2*	0,948	0,021785672
TREX2	-0,600	0,004042169	*LIMA1*	0,947	0,001518227
HAUS5	-0,601	0,000105643	*C4orf36*	0,946	1,56E-05
EXOSC8	-0,601	4,58E-06	*NEURL2*	0,942	0,026724013
CEP152	-0,602	0,004348111	*SEMA3B*	0,940	3,12E-05
ASNS	-0,602	0,00218666	*OSBPL3*	0,933	2,87E-14
FN3KRP	-0,603	2,08E-06	*LYPLAL1*	0,932	2,32E-07
WRAP53	-0,607	0,003155957	*SPRED3*	0,930	0,036256447
CENPL	-0,609	0,002767561	*CCNG1*	0,928	4,64E-10
LRRC20	-0,611	8,32E-05	*SAMD12*	0,927	0,012115056
MZT1	-0,612	2,80E-05	*MT1X*	0,926	0,011019225
NCK1-AS1	-0,612	0,016410201	*BBC3*	0,925	1,71E-07
CDC7	-0,613	0,003873351	*PIDD1*	0,925	0,000538773
POLD1	-0,613	5,54E-05	*FAM83H-AS1*	0,925	0,019932608
UNG	-0,614	3,22E-06	*CH17-360D5.2*	0,922	0,047878277
DCLRE1A	-0,615	9,51E-06	*ALOX12B*	0,918	2,38E-05
POLA1	-0,618	0,000149538	*IKBIP*	0,916	2,47E-17
TMPO	-0,618	3,29E-07	*PHPT1*	0,910	2,96E-05
CCDC18	-0,619	0,013354996	*TMBIM4_1*	0,905	0,000842653
WDR24	-0,623	0,015342171	*PADI3*	0,902	4,83E-05
HYLS1	-0,623	0,005374256	*ITGA3*	0,896	2,91E-22
DTYMK	-0,623	0,001377001	*CATSPERG*	0,895	0,046796417
TRIM36	-0,623	0,022179999	*CTD-2267D19.3*	0,894	1,06E-06
TMEM237	-0,627	8,33E-06	*RP11-421F16.3*	0,894	5,40E-11
SLC1A4	-0,627	0,00973185	*SLC16A6*	0,892	1,56E-06
PCNA	-0,628	1,92E-06	*LCAT*	0,889	0,000133924
B3GAT1	-0,631	0,001230776	*PABPC3*	0,887	0,015550326
UBL7-AS1	-0,631	0,040260532	*RGS16*	0,887	5,15E-06

CARHSP1	-0,632	0,000389885	*NACAP1*	0,887	0,002122135
GPD2	-0,633	2,32E-05	*MTRNR2L12*	0,887	0,037722861
ACSM3	-0,633	0,026651822	*ABCA3*	0,886	0,023533137
CPNE2	-0,633	0,000712796	*TNFAIP3*	0,882	0,025661595
SCLT1	-0,633	0,000332381	*CCND2*	0,881	1,95E-07
PPDPF	-0,636	0,00079687	*PBLD*	0,880	0,005054979
MEGF9	-0,639	0,020087007	*SDC4*	0,879	0,00061449
TFAP2A	-0,639	0,012367045	*LPCAT2*	0,879	2,83E-07
RPA3	-0,639	1,09E-05	*TRPV4*	0,878	0,003324685
GINS3	-0,640	0,002093119	*FBXO2*	0,877	0,004734152
INCENP	-0,640	1,66E-05	*TM7SF2*	0,877	0,005503886
UBR7	-0,643	9,27E-05	*MMP14*	0,876	2,63E-11
SLF1	-0,643	3,81E-07	*KCTD11*	0,876	5,76E-08
IRF7	-0,645	0,015409504	*SLC35F6*	0,874	0,007289271
FAM72A	-0,650	0,000767648	*MIR222HG*	0,872	0,008622546
TTPA	-0,653	0,024351758	*SP140L*	0,871	0,000958529
CEP57L1	-0,653	0,000110902	*AP000320.7*	0,870	0,001172173
PARP2	-0,655	2,02E-05	*PLCL2*	0,868	0,011259561
KPNA2	-0,656	4,95E-06	*WNT5A*	0,865	4,86E-07
NRM	-0,656	6,41E-06	*TRIM13*	0,865	0,00227056
LMO7	-0,658	0,022967544	*MRPS6*	0,862	9,52E-06
RP11-22B23.1	-0,661	0,000310442	*UNC5CL*	0,862	0,021361489
RAD21	-0,662	3,09E-05	*SERPINB8*	0,860	0,007387252
SPA17	-0,663	0,00170172	*ZNF561*	0,855	2,10E-08
STMN1	-0,665	8,29E-05	*IFRD1*	0,855	0,000872938
HIRIP3	-0,669	0,000210159	*LIG4*	0,854	0,004306649
YEATS4	-0,674	7,12E-06	*ITGA2*	0,854	1,46E-05
RTTN	-0,674	0,042811257	*GABARAPL1*	0,851	1,74E-10
DOCK11	-0,674	0,040564308	*ZFP36L1*	0,850	0,000816843
SASS6	-0,677	2,20E-05	*FBXO22*	0,846	1,64E-16
TONSL	-0,678	0,002373602	*LAMA3*	0,844	0,013433623
MCM8	-0,679	0,000260238	*TNFRSF14*	0,844	3,93E-05

FAM83D	-0,679	1,05E-07	*F8*	0,843	0,006945599
TIMELESS	-0,680	5,38E-05	*RORA*	0,842	0,008957324
PRADC1	-0,682	0,000245383	*HLA-DQB1*	0,839	0,015955324
RAD18	-0,683	0,000124524	*MTFR1L*	0,839	1,20E-08
BORA	-0,683	0,000200263	*TMEM117*	0,838	0,000573995
FOXA2	-0,684	0,041310187	*CHURC1*	0,834	2,65E-06
CHEK2	-0,689	8,82E-07	*ALOX5AP*	0,834	0,031189258
PHF19	-0,693	5,36E-05	*INAFM2*	0,828	6,17E-05
ECT2	-0,694	0,000740621	*APOBEC3C*	0,825	4,98E-10
BCL2L12	-0,696	0,006802911	*TRNP1*	0,823	3,47E-13
CENPE	-0,696	0,008754552	*GS1-124K5.4*	0,820	0,024385909
C2orf44	-0,697	0,030691486	*CCDC90B*	0,820	9,29E-08
SAP30	-0,698	6,92E-06	*AEN*	0,819	1,43E-07
DONSON	-0,698	0,005368066	*TCEAL1*	0,818	4,23E-06
CCSAP	-0,699	2,71E-07	*PLXNC1*	0,818	0,00103093
CCDC77	-0,701	1,29E-06	*DHRS12*	0,815	0,002177925
SKA2	-0,703	5,57E-06	*BIRC2*	0,815	1,57E-09
WHSC1	-0,703	2,19E-07	*GALNT10*	0,814	0,030612163
C16orf59	-0,706	0,006026328	*CD44*	0,814	7,53E-05
RAD9A	-0,707	2,81E-06	*EME2*	0,813	0,00014515
DDX11	-0,707	3,46E-09	*ADPRM*	0,813	0,000590895
POLE	-0,707	3,28E-05	*RP13-463N16.6*	0,812	0,000241341
FAAP24	-0,708	0,002231057	*AC084219.2*	0,811	0,003220438
HES4	-0,711	0,001419719	*BACH1*	0,810	0,011635117
CCNE2	-0,712	0,009584382	*RNF19A*	0,810	0,004011226
LIN54	-0,712	9,45E-06	*MR1*	0,809	0,000182653
MSH6	-0,712	3,76E-06	*WDR72*	0,809	0,000132139
BRCA2	-0,717	0,000991958	*CAPS*	0,806	0,005076424
SAPCD2	-0,717	4,26E-07	*EXT1*	0,806	8,99E-05
ACBD7	-0,718	0,000108261	*SLC12A4*	0,805	1,28E-06
KNSTRN	-0,719	1,41E-08	*CREBRF*	0,804	0,001687338
RAD51D	-0,721	0,022363606	*TMEM106B*	0,803	1,21E-09
HMGB3	-0,721	0,000102288	*ALS2CL*	0,802	0,001959104

FANCA	-0,723	2,46E-06	*PERP*	0,801	1,71E-08
ARL6IP6	-0,725	2,84E-06	*IDUA*	0,800	0,0018920 56
C4orf46	-0,725	3,03E-10	*CERS5*	0,798	3,12E-09
PXMP2	-0,726	6,21E-05	*LINC00941*	0,797	0,0010234 11
PARPBP	-0,726	3,99E-05	*ARRDC4*	0,796	0,0075570 49
MCM3	-0,728	3,29E-06	*TMC7*	0,789	0,0423524 11
GSTCD	-0,728	0,047380714	*LYRM5*	0,788	5,24E-10
ARHGAP19-SLIT1	-0,729	0,000301829	*SLC20A1*	0,788	8,40E-08
MMS22L	-0,729	0,001377001	*CYB5R2*	0,785	4,78E-05
NCAPH2	-0,734	0,000282201	*TLR3*	0,785	0,0310963 18
DNAJC9	-0,735	0,002920904	*ZNF30*	0,783	0,0003363 05
SMC2	-0,739	1,94E-05	*KLF6*	0,781	4,51E-07
CDK2	-0,740	6,12E-10	*FHL2*	0,780	8,65E-07
KDM4D	-0,740	0,024434838	*GBE1*	0,780	4,77E-06
HMGN2	-0,741	0,000687366	*FAM221A*	0,777	0,0022305 6
WDR62	-0,742	0,001628999	*MXRA8*	0,776	0,0102473 28
LIN9	-0,745	8,08E-09	*FCGBP*	0,775	0,0076323
NUP62	-0,746	0,015377135	*NOP14-AS1*	0,774	1,52E-06
MYBL1	-0,748	0,002890636	*FTX*	0,773	0,0009451 6
LIG1	-0,749	8,66E-08	*PIGF*	0,768	0,0006583 07
CHTF18	-0,750	2,54E-06	*BCL2L1*	0,766	7,67E-10
H2AFZ	-0,751	0,000558777	*IGFL4*	0,764	0,0448131 39
MPP2	-0,754	0,004950758	*DOCK9*	0,762	0,0046062 4
PROCA1	-0,754	0,035771077	*ZNF83*	0,760	3,66E-05
TMEM107	-0,754	1,05E-05	*IL32*	0,759	0,0483593 65
CDC25A	-0,755	5,64E-05	*NADSYN1*	0,755	0,0012430 69
OVOS2	-0,758	0,023612823	*MYEOV*	0,754	0,0039152 75
GGH	-0,758	1,17E-05	*TBC1D2*	0,753	0,0008736 7
CENPF	-0,758	0,000835349	*SDSL*	0,751	0,0019144 76
ATRNL1	-0,758	0,010267399	*EPS8L2*	0,750	0,0006490 08
GMNN	-0,759	2,56E-07	*KLK6*	0,750	0,0012345 54
DLEU2	-0,760	0,001440848	*SORBS2*	0,749	0,0143205 4

HMGB1	-0,765	2,99E-08	*GLS*	0,747	2,57E-05
C1QL1	-0,766	0,018738036	*PEAR1*	0,747	7,37E-05
CENPJ	-0,767	9,48E-07	*MBNL2*	0,746	9,37E-09
HAT1	-0,767	1,21E-07	*SLC30A1*	0,743	0,011550674
H1FX	-0,770	3,12E-05	*SRSF8*	0,740	6,86E-06
MSH5	-0,771	0,000340593	*MYOF*	0,740	6,48E-10
POLD3	-0,772	7,19E-07	*SLC22A15*	0,739	0,046857967
LRRCC1	-0,775	0,020216201	*CDC42EP3*	0,739	0,001635734
RFC4	-0,775	0,000134944	*ELFN2_1*	0,739	0,044405361
CENPP	-0,776	4,19E-07	*PAOX*	0,738	0,010956452
WEE1	-0,777	3,77E-07	*LAMB3*	0,737	0,000466931
SDHAF2	-0,779	0,003549174	*XPC*	0,734	2,31E-11
NEMP1	-0,779	2,70E-08	*ZNF561-AS1*	0,734	0,026277108
DDX12P	-0,780	3,28E-05	*SLC38A6*	0,732	2,35E-06
AUNIP	-0,782	0,000175826	*POLD4*	0,731	0,000550338
GINS4	-0,782	0,003181053	*CASS4*	0,731	0,047878277
MXD3	-0,785	2,23E-10	*ICA1*	0,731	0,011784098
HAUS8	-0,785	0,000242045	*AP1S2*	0,726	7,07E-08
CNTRL	-0,787	0,00069107	*ZNF284*	0,725	0,009300942
ENTPD2	-0,788	0,00994185	*TARSL2*	0,725	1,03E-08
CCNF	-0,790	3,28E-05	*ZNF792*	0,725	5,81E-06
ZNF724P	-0,790	0,012416555	*MRAS*	0,719	0,00989464
CHEK1	-0,792	2,90E-09	*MOSPD1*	0,719	3,24E-10
CABLES2	-0,795	0,000235275	*ZNF585A*	0,718	0,010553768
DBF4B	-0,800	2,46E-07	*DNTTIP1*	0,717	4,71E-08
RFC2	-0,800	0,000313882	*RIOK3*	0,717	8,67E-07
KIF20B	-0,800	0,000153805	*AMACR*	0,714	0,015589534
CCDC15	-0,800	0,008223661	*ZNF577*	0,713	0,001952945
UBALD2	-0,801	1,61E-10	*LPP*	0,713	0,029941943
ZWILCH	-0,802	1,36E-05	*RPL23AP7*	0,712	7,25E-05
NUP62CL	-0,802	9,89E-06	*SPATA7*	0,710	0,000770573
RHNO1	-0,803	0,002545655	*LAMC2*	0,710	0,001784492
ARL6IP1	-0,804	2,05E-11	*ZNF23*	0,710	7,24E-05
PKMYT1	-0,805	0,000151811	*OTUB2*	0,708	7,14E-06

RBL1	-0,805	6,23E-09	*RP4-717I23.3*	0,708	0,0054386 23
CENPW	-0,807	0,008088747	*IL1RL2*	0,707	0,0317876 02
TUBB4B	-0,807	1,13E-06	*ANKRD46*	0,707	8,62E-07
VRK1	-0,810	1,99E-07	*PCMTD1*	0,706	0,0008343 8
C17orf53	-0,811	8,62E-05	*CSNK1G1*	0,706	1,16E-12
ZNF519	-0,812	0,002166744	*BX322557.10*	0,704	0,0035005 35
MIS18A	-0,815	0,007974801	*SEC14L2*	0,703	3,75E-06
NCAPD3	-0,816	1,68E-11	*GRIP1*	0,702	0,0466399 01
FBLN1	-0,818	0,041310187	*SLC25A20*	0,702	0,0217817 59
CCDC150	-0,818	0,012305864	*CALCOCO1*	0,701	2,18E-06
MIR210HG	-0,818	0,023697935	*C5orf28*	0,698	0,0008013 44
SGOL2	-0,819	0,000177526	*SPATS2L*	0,698	1,93E-06
WDHD1	-0,819	2,51E-06	*ZNF235*	0,696	0,0199035 61
EFCAB11	-0,821	0,002473676	*C10orf11*	0,694	0,0004944 22
RRM1	-0,822	8,30E-07	*TM7SF3*	0,693	1,79E-16
ARHGAP11B	-0,823	1,74E-06	*IMMP2L*	0,692	2,68E-07
LMNB1	-0,823	1,73E-09	*TMCC1-AS1*	0,692	0,0077522 78
SLC30A3	-0,824	0,004376498	*KRT5*	0,691	9,44E-08
ZNF700	-0,825	0,033123544	*LIPA*	0,691	2,27E-06
TRIM59	-0,828	0,000725648	*SQRDL*	0,690	0,0022656 39
CORO1A	-0,829	0,028932252	*CYLD*	0,688	0,0023836 53
ZNF331	-0,831	0,000503448	*HMOX1*	0,688	0,0219691
GNG7	-0,832	0,001318662	*CTD-2366F13.1*	0,687	0,0055038 86
CEP128	-0,833	0,000946058	*MAP6*	0,687	0,0284309 59
SPDL1	-0,834	9,72E-05	*YPEL5*	0,687	0,0201129 27
LIPT2	-0,835	0,040564308	*CDC42BPG*	0,685	0,0072892 51
TAF5	-0,839	4,97E-07	*SNAP23*	0,681	7,89E-07
CTD-2207O23.3	-0,839	0,018723084	*EI24*	0,681	7,55E-05
CHAF1B	-0,841	8,89E-05	*TEX9*	0,679	0,0263182 79
DCK	-0,844	2,18E-08	*SSR3*	0,678	0,0005602 68
FGFR3	-0,847	0,003689073	*DAB2*	0,678	0,0466938 21
C1orf112	-0,847	0,000232408	*ELK3*	0,676	0,0018354 52

EDAR	-0,848	0,002373602	*DUXAP8_1*	0,676	1,93E-08
CENPM	-0,850	7,26E-07	*PTK6*	0,676	0,006953275
DNA2	-0,850	6,01E-05	*TNFRSF10B*	0,676	2,02E-14
NCAPD2	-0,851	8,08E-09	*NINJ1*	0,675	0,000155465
HR	-0,851	0,00117744	*DNAH17*	0,672	0,01225677
ARHGAP11A	-0,852	5,95E-09	*TAB3*	0,671	3,12E-09
GINS1	-0,853	3,12E-09	*BLCAP*	0,671	1,41E-08
MKI67	-0,855	8,62E-05	*UBE2H*	0,668	2,19E-12
PRR11	-0,855	1,22E-05	*CAV2*	0,667	0,000186513
CENPQ	-0,855	0,000124715	*ATG4A*	0,667	0,003214146
KIF18A	-0,856	2,33E-05	*CPEB4*	0,666	0,000606932
KIF23	-0,856	1,21E-08	*TMEM17*	0,666	0,034230488
PRIM1	-0,856	2,04E-07	*ZNF337*	0,666	3,25E-05
YPEL1	-0,859	0,005969591	*DGKA*	0,665	2,79E-09
KIAA0101	-0,860	1,58E-06	*ZNF195*	0,664	0,000717455
TCF19	-0,864	0,000197222	*ANXA2*	0,664	6,21E-06
MCM6	-0,864	2,30E-09	*SHC1*	0,664	1,04E-06
XRCC2	-0,867	5,34E-06	*MT-ATP8*	0,663	9,49E-13
CLSPN	-0,867	0,000365123	*SLC5A3*	0,660	0,013414239
UHRF1	-0,869	0,00177335	*ZNF252P*	0,659	0,000698072
MARVELD3	-0,870	0,024918508	*C11orf54*	0,659	0,025917539
TACC3	-0,872	8,03E-06	*RNF182*	0,658	0,000958529
POC1A	-0,876	4,48E-05	*NME7*	0,658	8,56E-06
ATP2C2	-0,879	0,001440848	*TMEM68*	0,658	0,001240502
DUT	-0,881	1,57E-09	*IMPACT*	0,655	2,48E-05
TUBA1B	-0,881	7,99E-10	*MFSD1*	0,650	9,07E-05
MCM4	-0,881	4,59E-08	*TRIB1*	0,650	5,78E-05
SKA3	-0,882	7,86E-05	*FAM172A*	0,650	0,006214533
FAM110A	-0,883	0,000108966	*AP1S3*	0,649	0,000768686
RP5-1120P11.1	-0,884	0,021176613	*TMEM50B*	0,648	0,004972455
FANCG	-0,886	2,08E-09	*ZNF461*	0,648	0,025488144
PRKAG2-AS1	-0,887	0,015967585	*RPL22L1*	0,646	0,000289082
MCM7	-0,887	1,98E-08	*C21orf91*	0,646	0,006342788

AC004381.6	-0,888	0,003493334	*GPATCH11*	0,645	0,0002285<br>04
NEMP2	-0,889	0,045229343	*ABL2*	0,645	9,78E-05
MTBP	-0,892	9,72E-05	*ZNF440*	0,644	0,0035980<br>11
MCM2	-0,893	5,79E-09	*ANK2*	0,643	0,0190811<br>83
C19orf57	-0,894	0,004323147	*GNPDA1*	0,641	2,96E-05
STIL	-0,897	1,50E-08	*NOL6*	0,640	0,0057888<br>13
RECQL4	-0,898	4,97E-07	*MEGF11*	0,637	0,0263001<br>29
DSN1	-0,901	4,07E-11	*TEP1*	0,637	4,23E-06
UBE2C	-0,902	1,91E-09	*ACTN1*	0,636	5,49E-09
RP5-991G20.1	-0,902	0,007874601	*MLPH*	0,635	0,0115141<br>95
TRIP13	-0,906	9,16E-10	*CROT*	0,634	0,0013206<br>67
CDKN3	-0,907	6,36E-09	*TSPAN31*	0,634	0,0001809<br>21
RP11-849H4.2	-0,907	0,018069111	*POLH*	0,634	8,34E-06
POLA2	-0,908	2,69E-12	*BTBD10*	0,633	0,0002533
HELLS	-0,909	2,45E-08	*ASAP1*	0,633	1,50E-05
PTTG1	-0,909	1,05E-10	*KRT8*	0,633	0,0007108<br>21
KIF4A	-0,912	2,92E-05	*ZNF225*	0,632	0,0159098<br>28
CENPN	-0,912	1,33E-06	*HSPA4L*	0,631	6,65E-07
CTSV	-0,914	8,83E-09	*PLCD3*	0,630	6,08E-10
RFC3	-0,914	1,24E-07	*CST2*	0,630	0,0289765<br>48
YBX2	-0,915	0,000345818	*PMEPA1*	0,629	0,0001270<br>3
RACGAP1	-0,917	2,23E-10	*BIK*	0,629	8,51E-05
ATAD5	-0,917	3,27E-06	*APP*	0,628	1,25E-05
RFC5	-0,917	2,93E-10	*ERVMER34-1*	0,627	0,0020801<br>73
FAM72B	-0,918	0,022064317	*ZNF446*	0,626	0,0114093<br>6
ZWINT	-0,920	1,86E-06	*ZNF420*	0,625	0,0086706<br>75
CH507-24F1.1	-0,922	0,049837351	*ZNF354A*	0,624	0,0007341<br>27
ID3	-0,923	0,031317712	*THSD4*	0,624	0,0004064<br>61
POLE2	-0,925	1,76E-05	*LAMTOR3*	0,622	0,0005828<br>07
MELK	-0,925	2,68E-07	*HMGA2*	0,622	0,0001508<br>85
BARD1	-0,926	0,000794962	*CHIC1*	0,621	0,0051184<br>79
CHAF1A	-0,926	3,40E-06	*ZNF613*	0,620	0,0329528<br>07
IL22RA1	-0,928	0,000279109	*SUSD6*	0,619	8,53E-06

UBE2S	-0,929	1,26E-07	*MMAA*	0,618	0,011122394
HAUS3	-0,929	0,048239638	*HEATR5B*	0,616	0,000586637
C21orf58	-0,930	4,96E-05	*KCNAB2*	0,616	0,00455439
NCAPG2	-0,930	7,99E-10	*TNFAIP1*	0,616	2,66E-05
TLE2	-0,931	0,048432003	*S100A11*	0,616	3,55E-05
FBXO5	-0,932	3,07E-05	*CD58*	0,615	0,000858143
KIF24	-0,933	0,000253332	*NTPCR*	0,615	8,19E-07
TMPO-AS1	-0,933	0,000766001	*MT-ND4L*	0,615	3,54E-05
KNTC1	-0,933	4,47E-05	*CLN8*	0,614	0,000284544
CENPO	-0,939	8,06E-09	*SLC38A7*	0,613	0,000382877
KIAA1524	-0,939	4,17E-06	*HIST3H2A*	0,613	0,021733881
TPX2	-0,940	1,30E-09	*SLC17A5*	0,613	1,64E-06
AURKA	-0,943	4,87E-10	*RASGRF1*	0,612	0,000872938
ZNF367	-0,947	1,33E-06	*INPP1*	0,611	8,67E-06
CIT	-0,949	3,29E-05	*TGFBI*	0,611	7,24E-06
CDC20	-0,951	1,42E-07	*C18orf32*	0,610	0,003145561
DNMT3B	-0,951	0,000237273	*ZNF674*	0,610	0,024987735
KIF22	-0,952	7,56E-10	*RHOBTB1*	0,609	0,033443019
GPR137C	-0,952	0,025566903	*TBX3*	0,608	0,005141854
SMC4	-0,952	1,33E-06	*LENG9*	0,607	0,003454041
CDCA2	-0,952	1,01E-07	*IP6K2*	0,605	9,66E-05
C14orf80	-0,953	8,62E-05	*GPRC5A*	0,605	0,04296355
BRCA1	-0,955	1,30E-08	*FOSL1*	0,603	0,010354709
CENPK	-0,956	1,29E-06	*PGBD2*	0,603	0,008973947
DSCC1	-0,958	1,05E-06	*DCAF10*	0,601	0,015462096
FAM111B	-0,959	0,000356973	*RRAGC*	0,600	0,000607315
ARHGEF16	-0,962	0,003014164	*RP11-545J16.1*	0,600	0,012692959
ASPM	-0,962	9,99E-07	*ZNF181*	0,598	3,68E-05
NXPH4	-0,962	5,24E-07	*MTMR10*	0,598	0,002016198
CDCA5	-0,962	3,37E-07	*LRP1*	0,597	0,032762095
ATAD2	-0,969	8,92E-08	*ZNF556*	0,596	0,027824112

DIAPH3	-0,969	1,36E-08	*BTG1*	0,595	0,0004912 25
FANCI	-0,974	2,84E-09	*CTBS*	0,595	0,0352557 73
RHEBL1	-0,976	4,76E-07	*CARNMT1*	0,594	5,08E-05
CDH1	-0,978	0,027872854	*FAM214A*	0,594	0,0006662 53
ORC6	-0,978	7,24E-08	*ZNF226*	0,593	0,0018424 5
CASC5	-0,978	2,46E-07	*C11orf1*	0,593	0,0152179 22
H2AFX	-0,981	2,07E-05	*RANBP10*	0,592	8,27E-07
RP11-762I7.5	-0,981	0,031191975	*BBS4*	0,591	0,0012827 36
MYBL2	-0,987	4,09E-09	*ZNF600*	0,591	0,0009412 56
KIF14	-0,988	1,46E-05	*ADAM19*	0,591	9,23E-05
PRKAR2B	-0,988	0,000279108	*SH3BGRL3*	0,591	0,0063134 48
CDKN2C	-0,988	7,17E-09	*EXOC4*	0,590	8,34E-06
CCNB1	-0,988	5,10E-12	*SGK223*	0,590	7,09E-05
POLQ	-0,993	6,21E-07	*S100A10*	0,590	0,0005199 02
ORC1	-0,995	7,34E-09	*ATP6V0E1*	0,590	0,0001948 07
DHFR	-0,996	3,27E-07	*DISP2*	0,589	0,0003085 69
RRM2	-0,997	1,56E-07	*PRKAB2*	0,589	0,0003266 13
ZGRF1	-1,001	2,22E-09	*ARL4C*	0,586	4,54E-05
MCM5	-1,001	3,96E-08	*FANK1*	0,586	0,0429828 56
MTFR2	-1,003	2,44E-09	*ENPP5*	0,586	0,0302011 75
CCNB2	-1,003	1,37E-11	*S100A6*	0,585	5,26E-05
BUB1B	-1,004	6,73E-10	*PIK3R2*	0,585	0,0216377 01
TROAP	-1,004	8,81E-13	*TAF13*	0,584	0,0012402 22
TRAIP	-1,005	1,86E-06	*KBTBD3*	0,584	0,0493980 7
CDC6	-1,005	6,16E-08	*LTB*	0,583	0,0276248 27
HMMR	-1,007	1,71E-08	*COL7A1*	0,583	0,0319136 34
NCAPG	-1,008	1,65E-07	*RIN2*	0,582	0,0155299 83
KIF11	-1,009	1,18E-09	*KRT19*	0,582	1,74E-06
MAD2L1	-1,011	4,84E-10	*GAA*	0,582	0,0495832 43
BRIP1	-1,011	2,36E-06	*RP11-958N24.1*	-0,581	0,0315231 46
MCM10	-1,013	3,90E-07	*SOGA3_1*	-0,581	0,0347894 64

ESPL1	-1,013	7,34E-09	*HNRNPUL2-BSCL2*	-0,582	0,000450606
TTK	-1,015	3,10E-06	*MEGF9*	-0,583	0,021940787
OIP5	-1,018	2,99E-09	*ATF5*	-0,584	0,035265896
KIF15	-1,020	5,85E-07	*RP11-347C12.1*	-0,584	0,020674542
PSMC3IP	-1,022	4,19E-07	*FANCE*	-0,584	0,001726722
GTSE1	-1,023	1,05E-08	*RP11-514P8.7*	-0,585	0,026845285
MASTL	-1,024	1,20E-09	*TSPAN33*	-0,586	0,031552339
CENPH	-1,027	3,28E-12	*FHOD1*	-0,587	0,002094599
KIF18B	-1,027	3,87E-09	*ADRBK1*	-0,587	1,88E-07
BIRC5	-1,029	1,35E-09	*P3H3*	-0,588	0,00400559
RP11-244M2.1	-1,030	0,034696345	*PTMS*	-0,588	7,25E-07
ARHGEF39	-1,030	9,81E-09	*PMM1*	-0,588	0,000434791
TOP2A	-1,032	8,92E-08	*GMNN*	-0,589	7,52E-05
ESCO2	-1,034	1,01E-08	*MEIS3*	-0,589	0,015799407
RNASEH2A	-1,039	6,82E-09	*ZNF714*	-0,590	0,000734172
DEPDC1	-1,039	1,36E-08	*RFX1*	-0,591	0,000143543
OAS3	-1,041	0,001884149	*ECT2*	-0,591	0,003518385
HJURP	-1,049	1,28E-07	*TUBGCP3*	-0,595	4,52E-08
NUSAP1	-1,050	1,81E-10	*DDX39A*	-0,595	9,79E-05
HAUS4	-1,051	2,79E-05	*FZR1*	-0,596	8,58E-06
RAD51	-1,054	7,38E-10	*RP11-1212A22.1*	-0,597	2,27E-05
SHCBP1	-1,055	1,44E-08	*CDCA4*	-0,597	2,60E-05
FANCB	-1,056	6,45E-07	*UQCC2*	-0,598	2,27E-06
DEPDC1B	-1,056	1,61E-10	*LIN9*	-0,598	3,72E-06
DTL	-1,056	4,52E-08	*RIMKLA*	-0,598	0,006820742
WDR76	-1,056	2,22E-06	*PSIP1*	-0,598	3,57E-09
PSRC1	-1,056	6,45E-10	*POLD3*	-0,599	0,000132531
UBE2T	-1,058	1,86E-12	*PIGS*	-0,599	2,00E-05
TP73	-1,058	7,30E-05	*DCLRE1B*	-0,603	7,53E-05
KIF20A	-1,060	3,24E-12	*IGSF9*	-0,603	0,006141125
ASF1B	-1,060	2,25E-06	*NFKBID*	-0,605	0,004803755
CDCA8	-1,062	1,73E-09	*PET100*	-0,606	0,005529367
APOBEC3B	-1,063	9,75E-06	*PAFAH1B3*	-0,607	2,88E-08

PLK4	-1,063	2,27E-13	*BUD13*	-0,609	3,74E-07
NCAPH	-1,063	1,48E-08	*ARHGEF19*	-0,609	0,001116721
GAS2L3	-1,065	9,92E-08	*CNTNAP1*	-0,611	0,025762987
ANLN	-1,067	1,05E-06	*NKX6-1*	-0,613	0,038123128
PRC1	-1,068	1,49E-10	*MED30*	-0,613	0,001269612
C2orf48	-1,070	0,010992055	*GPR153*	-0,613	0,00063635
E2F2	-1,070	7,36E-05	*CROCCP2_1*	-0,614	0,001634172
BUB1	-1,070	1,24E-10	*DZIP1L*	-0,614	0,005076424
EXO1	-1,070	5,39E-07	*MTFP1*	-0,615	3,74E-06
PLK1	-1,072	3,06E-10	*CCDC9*	-0,615	1,18E-05
RAD54B	-1,072	4,32E-07	*CCDC28B*	-0,616	0,01154732
CENPI	-1,078	2,70E-08	*THOC6*	-0,616	8,44E-09
CEP55	-1,079	1,12E-08	*FGFRL1*	-0,616	0,000172892
EID1	-1,079	0,000554088	*LIN54*	-0,618	0,000109863
TICRR	-1,080	2,58E-05	*HCP5*	-0,620	0,03770186
CDC45	-1,081	6,68E-10	*ALYREF*	-0,620	0,000950924
DERL3	-1,081	2,65E-05	*SERPINB9*	-0,621	0,000766708
DRP2	-1,082	0,038235764	*RAD21*	-0,622	6,36E-05
DLGAP5	-1,086	9,41E-10	*RANBP1*	-0,624	1,48E-06
LMCD1	-1,087	0,027970488	*LSM4*	-0,624	5,11E-05
CENPA	-1,088	1,78E-08	*FZD7*	-0,625	7,71E-05
CDK1	-1,089	4,85E-10	*CNFN*	-0,625	0,026927644
FAM64A	-1,089	2,59E-10	*DCXR*	-0,627	0,000582894
NDC80	-1,091	3,62E-08	*CROCCP2_2*	-0,627	0,004383209
MYB	-1,091	0,001455383	*TMPO*	-0,628	9,79E-08
SKA1	-1,094	2,71E-06	*RP11-362F19.1*	-0,628	0,003512867
AC002116.7	-1,095	0,003458474	*MAP3K6*	-0,628	0,008363715
AURKB	-1,095	9,88E-12	*METRN*	-0,629	0,030271027
SPC24	-1,096	4,97E-08	*WEE1*	-0,631	3,86E-05
FANCD2	-1,098	4,56E-10	*CILP2*	-0,631	0,031845211
GINS2	-1,106	5,61E-09	*CEP131*	-0,632	5,20E-05
CDCA3	-1,108	4,90E-14	*FAM72A*	-0,632	0,000601298

RIBC2	-1,112	0,004366858	*RP1-151F17.1*	-0,633	0,02587672
FAM105A	-1,112	0,004440729	*GALNT12*	-0,633	0,006559813
C18orf54	-1,113	8,26E-05	*Sep 04*	-0,634	0,000732472
SGOL1	-1,115	2,45E-07	*HSD17B6*	-0,634	0,024783066
CDT1	-1,116	1,61E-07	*CBR3*	-0,634	0,031189258
CENPU	-1,117	1,93E-09	*CCDC77*	-0,634	9,56E-06
CCNA2	-1,118	1,36E-08	*SLX4*	-0,634	0,000139111
E2F1	-1,120	3,76E-10	*CENPL*	-0,634	0,000816843
NRGN	-1,123	1,95E-09	*RFC3*	-0,637	0,000358737
MND1	-1,126	1,41E-08	*COL26A1*	-0,638	0,013437246
IQGAP3	-1,127	2,25E-06	*OVOL2*	-0,638	0,04743103
BLM	-1,129	9,36E-08	*RIPK4*	-0,640	0,002307831
SERTAD4	-1,134	0,005080172	*KPNA2*	-0,640	5,06E-06
PIF1	-1,135	4,37E-10	*TCTN2*	-0,641	0,002513739
FOXM1	-1,138	3,53E-10	*SMO*	-0,641	0,006940719
XRCC3	-1,147	1,11E-08	*C9orf142*	-0,641	0,000311277
LCK	-1,157	0,003796094	*NASP*	-0,641	3,10E-08
SPAG5	-1,159	3,35E-09	*CDKN2A*	-0,642	1,81E-06
MNS1	-1,164	0,012100336	*RP11-599B13.6*	-0,643	0,02909516
RAD51AP1	-1,165	9,11E-10	*ADORA1*	-0,643	0,029111106
HMGB2	-1,168	3,89E-14	*KLHL25*	-0,645	1,32E-06
RMI2	-1,174	2,05E-14	*KNSTRN*	-0,646	2,58E-07
KIF2C	-1,178	7,65E-11	*ARL6IP1*	-0,646	6,94E-08
ESAM	-1,180	0,033734909	*VWA1*	-0,648	0,001098391
KIFC1	-1,182	1,05E-10	*PRR34-AS1*	-0,648	0,032320863
FEN1	-1,187	6,35E-06	*FANCC*	-0,649	4,55E-05
TK1	-1,209	2,75E-11	*DOT1L*	-0,649	9,61E-06
SPC25	-1,209	2,81E-09	*TMEM52*	-0,650	0,005076424
NUF2	-1,219	8,08E-11	*ASNS*	-0,650	0,000382101
CKAP2L	-1,223	1,32E-13	*SP6*	-0,650	0,001984904
E2F8	-1,224	9,96E-09	*SKA2*	-0,651	2,04E-05
HPSE	-1,226	2,74E-08	*PER1*	-0,651	0,002269845

CARD8	-1,229	0,001193163	*NABP2*	-0,651	1,02E-11
CSH2	-1,235	4,52E-08	*IGFLR1*	-0,653	0,0204608 37
RDM1	-1,241	5,11E-09	*PMEL*	-0,654	0,0088694 23
CKS1B	-1,242	1,03E-10	*IMPA2*	-0,655	3,28E-05
NMU	-1,242	4,72E-13	*CNTROB*	-0,656	3,04E-10
NEK2	-1,244	8,40E-12	*SP4*	-0,656	0,0003690 83
NEIL3	-1,259	1,09E-09	*FGFR4*	-0,658	0,0008919 78
STMND1	-1,264	0,015092016	*NT5DC2*	-0,659	0,0002853 06
ARHGAP33	-1,268	2,31E-07	*PHIP*	-0,660	0,0001093 85
PBK	-1,268	1,03E-12	*FKBP5*	-0,660	2,70E-08
RAD54L	-1,273	6,82E-12	*C16orf93*	-0,661	0,0045007 98
ZNF492	-1,273	0,022064317	*ACD*	-0,662	4,40E-06
FAM72D	-1,285	7,98E-07	*LRRC45*	-0,663	0,0008955 71
CLGN	-1,297	0,029234406	*RASSF2*	-0,663	0,0158464 21
GPR19	-1,316	0,000599146	*RASIP1*	-0,664	0,0365064 59
C2CD4C	-1,318	0,030246446	*PHLPP1*	-0,664	0,0093831 15
CDC25C	-1,324	6,70E-14	*PPIH*	-0,664	3,38E-05
AC005076.5	-1,325	0,039117899	*MMS22L*	-0,664	0,0026111 26
SUV39H1	-1,346	0,015806828	*PBX1*	-0,664	0,0111298 02
DDIAS	-1,373	0,000994535	*AC007277.3*	-0,665	0,0006838 92
SERTAD4-AS1	-1,373	0,044577328	*SEMA6C*	-0,665	0,0489533 32
EME1	-1,384	6,50E-09	*CDC25A*	-0,667	0,0002977 59
CH507-145C22.1	-1,384	1,46E-05	*SYNGR1*	-0,668	0,0328056 85
RTKN2	-1,478	4,29E-07	*HAT1*	-0,669	3,49E-06
C5orf34	-1,480	9,90E-09	*CLASRP*	-0,671	1,12E-10
SLPI	-1,494	0,000338532	*TCF7*	-0,672	2,79E-09
TCAM1P	-1,505	0,011994168	*C11orf84*	-0,673	9,17E-10
KIAA0391	-1,549	6,75E-07	*LRP4*	-0,673	0,0009231 47
ZNF695	-1,594	0,006765353	*PXMP4*	-0,673	0,0007401 79
RASD1	-1,632	0,027200236	*ANAPC11*	-0,674	1,07E-05
FXYD6	-1,669	0,000116367	*OTX1*	-0,676	0,0484429 12
DMBX1	-1,678	0,036842853	*FBXL16*	-0,679	0,0067500 86

ZNF736	-1,679	0,00994185	*FAM46B*	-0,679	0,0002719<br>12
NXPH3	-1,693	0,038580231	*SCLT1*	-0,679	5,71E-05
AP000251.3	-1,784	0,006160276	*THRA*	-0,681	1,06E-06
SP8	-1,785	0,010642966	*GGH*	-0,681	6,63E-05
CCDC121	-1,832	0,017485145	*ZNF503*	-0,682	0,0009585<br>29
IRF8	-1,848	0,004521785	*RP11-77P6.2*	-0,684	0,0237292<br>87
CNTNAP2	-1,866	0,000239909	*TMEM54*	-0,685	0,0072584<br>53
LHX9	-1,903	0,01840839	*C2orf68*	-0,687	0,0203814<br>3
DMC1	-1,949	0,033022532	*RGS3*	-0,687	0,0011285<br>5
GLI1	-2,098	0,013624157	*FBF1_1*	-0,688	0,0014398<br>04
C2orf78	-2,710	9,38E-05	*GOLGA2P7*	-0,688	0,0004472<br>69
			BAHCC1	-0,688	0,0121090<br>19
			ACBD7	-0,688	0,0001050<br>95
			KIF1A	-0,689	0,0155813<br>59
			RAB3IL1	-0,689	0,0383166<br>25
			KCNK5	-0,689	1,55E-09
			AC006486.9	-0,691	0,0108677<br>67
			CKB	-0,693	7,23E-11
			LINC01132	-0,694	0,0361023<br>89
			DCK	-0,695	4,76E-06
			SIVA1	-0,695	0,0001033<br>29
			GINS1	-0,696	1,39E-06
			ASCL2	-0,696	0,0102126<br>53
			CEP152	-0,696	0,0002975<br>35
			ARID5A	-0,696	2,24E-05
			DHRS13	-0,697	0,0042972<br>07
			NFATC1	-0,697	0,0093942<br>83
			RHNO1	-0,698	0,0061499<br>13
			H2AFV	-0,699	4,65E-08
			MSH6	-0,701	2,98E-06
			RP11-77K12.1	-0,702	0,0418294<br>82
			TRIM15	-0,703	0,0253215<br>65

FAM111B	-0,704	0,0100160<br>68
RAB26	-0,704	0,0093418<br>53
SIGIRR	-0,705	1,64E-05
RTN4R	-0,705	0,0026298<br>43
HSPA2	-0,706	0,0017844<br>92
PALM	-0,707	0,0091891<br>39
SMC1A	-0,708	1,44E-07
SMC2	-0,709	2,67E-05
SNHG19	-0,712	8,65E-07
OLFM1	-0,712	0,0051496<br>99
CALM3	-0,712	8,05E-06
AC253572.1	-0,714	0,0396480<br>48
FUT1	-0,715	0,0023225<br>09
DNAJC9	-0,715	0,0022045<br>98
ARL6IP6	-0,716	2,05E-06
PRX	-0,718	0,0039493<br>48
IDH2	-0,719	9,66E-06
DDTL	-0,722	0,0001513<br>36
CHST15	-0,722	2,94E-06
SLC1A4	-0,722	0,0008966<br>64
CBX2	-0,722	1,46E-05
MTBP	-0,722	0,0017355<br>98
CEP85	-0,723	5,77E-07
SSBP3	-0,724	5,07E-08
SH2B2	-0,725	0,0008729<br>38
LMO2	-0,725	8,98E-17
FAAP24	-0,725	0,0006729<br>75
MYO19	-0,725	2,55E-13
PER2	-0,727	0,0362046<br>57
GRTP1	-0,728	0,0496138<br>43
TUBG1	-0,728	3,22E-07
SUV420H2	-0,728	0,0012542<br>41
DPF1	-0,728	0,0053217<br>4

ASAP3	-0,731	0,0466876<br>42
CCSAP	-0,732	2,57E-08
RASD2	-0,733	0,0052499<br>85
RAET1E-<br>AS1_1	-0,734	0,0205352<br>28
B4GALNT1	-0,734	0,0019960<br>41
RP13-672B3.2	-0,735	0,0027994<br>22
EID1	-0,736	0,0223957<br>53
NKD2	-0,737	0,0180927<br>7
RHOV	-0,737	1,57E-05
ODF2	-0,738	1,85E-09
SASS6	-0,740	1,40E-06
CTD-<br>3092A11.1	-0,740	0,0198629<br>46
BRICD5	-0,740	0,0138520<br>34
PARPBP	-0,741	1,30E-05
ATP8B3	-0,741	3,20E-06
SLC25A10	-0,741	4,10E-11
CCDC15	-0,742	0,0090621<br>51
KIAA0101	-0,743	3,11E-05
PXN-AS1	-0,750	0,0155895<br>34
RP11-<br>315D16.2	-0,751	0,0049114<br>42
RAD54B	-0,751	0,0006052<br>3
PAK4	-0,752	2,06E-09
CDH24	-0,753	1,61E-05
PGP	-0,754	5,99E-06
MNX1	-0,754	7,14E-06
C1orf112	-0,754	0,0008499<br>36
HMGB3	-0,754	2,28E-05
PARP2	-0,758	1,95E-07
SP9	-0,758	0,0001043<br>55
KREMEN1	-0,759	0,0006363<br>5
JDP2	-0,759	0,0033366<br>9
CHEK2	-0,761	1,60E-08
CENPK	-0,761	0,0001329<br>11

AUNIP	-0,763	0,0001291 05
METRNL	-0,764	0,0003170 56
MYBL1	-0,764	0,0012293 2
TFAP2A	-0,766	0,0005372 82
HCN2	-0,766	0,0028270 6
TRIM59	-0,766	0,0012956 75
GLI2	-0,767	6,21E-05
CRABP2	-0,767	4,12E-06
CENPP	-0,768	2,83E-07
EFNA3	-0,771	4,52E-08
LINC00868	-0,772	0,0047083 42
DNA2	-0,773	0,0002051 25
CCHCR1	-0,774	2,94E-09
INAFM1	-0,776	0,0008736 7
NMB	-0,778	0,0019948 55
DOCK11	-0,779	0,0068362 47
MCM6	-0,779	5,11E-08
MCM3	-0,779	2,40E-07
CBR3-AS1	-0,781	0,0249958 66
RRM1	-0,781	1,85E-06
SOCS3	-0,782	0,0001259 67
EMC9	-0,782	2,22E-06
PRIM1	-0,782	1,45E-06
ANKRD9	-0,783	1,88E-06
RTTN	-0,784	0,0068362 37
WDHD1	-0,784	4,12E-06
RFC5	-0,786	4,66E-08
PASK	-0,786	1,72E-08
LMTK3	-0,788	0,0001356 19
POLR2J3	-0,788	0,0018627 8
RP11-386G11.10	-0,788	8,79E-05
LRRC75A	-0,790	0,0009698 78
FAM222A	-0,791	0,0007069 36
TNNT1	-0,791	7,06E-09

COX17	-0,793	3,44E-10
DLX4	-0,793	0,00029947
SH3BP1	-0,794	1,07E-06
PIM1	-0,795	9,92E-05
WDR24	-0,796	0,000249089
TMEM56	-0,797	0,001835452
RADIL	-0,797	0,014054194
B3GAT1	-0,800	6,61E-06
CYP27A1	-0,802	0,00987118
BCL2L2	-0,803	0,020003408
SLC29A4	-0,803	0,048660818
TTPA	-0,803	0,001443869
CSPG5	-0,803	0,0287238
REEP4	-0,804	6,91E-17
OVOS2	-0,807	0,007482888
LRRC20	-0,807	1,74E-08
PPP1R3G	-0,808	0,044750191
HMGB1	-0,810	1,71E-09
MIS18A	-0,810	0,004522327
NOS2	-0,810	0,003215987
ADAMTS15	-0,810	4,67E-05
PRADC1	-0,811	9,36E-07
DNMT1	-0,812	3,17E-07
CDK2	-0,812	2,02E-12
MCM8	-0,813	2,69E-06
GPC2	-0,813	0,034952696
HELLS	-0,813	4,97E-07
POLE2	-0,813	0,000121507
SH3RF3	-0,814	0,000166036
CENPN	-0,815	1,23E-05
NLGN2	-0,816	7,15E-07
LMO7	-0,816	0,001137187
GPRIN1	-0,816	9,29E-10
SNTA1	-0,817	0,006745342
MTFR2	-0,818	1,02E-06

BOLA2B	-0,818	0,0039072<br>23
ATRNL1	-0,818	0,0022484<br>66
PLEKHA4	-0,819	0,0001325<br>31
RAD9A	-0,819	9,34E-09
FAM83D	-0,820	1,96E-11
NEMP1	-0,821	1,75E-09
SV2A	-0,821	7,36E-05
H2AFZ	-0,821	6,24E-05
RFC4	-0,822	2,27E-05
MTL5	-0,822	0,0046807<br>62
EEF1A2	-0,825	0,0030648<br>25
POU6F1	-0,826	0,0332677<br>06
TREX2	-0,827	7,24E-06
SPDL1	-0,827	6,69E-05
KCNQ2	-0,827	0,0239678<br>38
HAUS5	-0,827	6,38E-09
SALL2	-0,827	0,0140854<br>39
MAD2L1	-0,829	3,33E-07
FANCB	-0,829	0,0001111<br>23
RCCD1	-0,830	4,57E-13
ZNF367	-0,831	1,99E-05
GRK6	-0,832	9,68E-15
LIN7B	-0,833	0,0442800<br>23
AMH	-0,836	0,0025295<br>78
PLEKHH3	-0,836	3,08E-06
FN3KRP	-0,838	1,33E-12
RFC2	-0,838	6,98E-05
WDR90	-0,839	1,10E-05
RNF157	-0,841	0,0208742<br>57
CENPJ	-0,841	2,22E-08
ATP2C2	-0,841	0,0009585<br>29
PRKAR2B	-0,842	0,0021341<br>43
TMEM107	-0,843	2,81E-07
KIF23	-0,845	9,28E-09
H1F0	-0,845	1,04E-06
CTD-<br>2033A16.3	-0,848	0,0034194<br>84

SBK1	-0,849	0,002239924
DTYMK	-0,849	1,41E-06
FBXO5	-0,850	0,000110689
CHAF1B	-0,850	3,83E-05
AKAP2	-0,850	0,027457513
NSUN7	-0,851	0,029630056
STRIP2	-0,853	0,003452472
HYLS1	-0,854	1,49E-05
WASH4P	-0,857	0,042772342
SGOL2	-0,857	4,04E-05
FAM171A2	-0,857	0,003228018
HES4	-0,858	1,65E-05
BCAM	-0,858	2,67E-06
ABHD8	-0,859	3,16E-06
TNFRSF13C	-0,859	0,03500484
TAF5	-0,860	9,81E-08
ZNF519	-0,863	0,000530981
MEX3D	-0,863	7,36E-07
FOXL1	-0,864	7,12E-05
CPNE2	-0,864	2,40E-07
TCOF1	-0,866	1,83E-09
BRCA2	-0,866	1,61E-05
CEP128	-0,866	0,000272792
GOLGA6L7P	-0,868	0,010482445
MAD2L2	-0,869	6,78E-12
UQCC3	-0,869	2,10E-08
GNAO1	-0,869	2,51E-07
AUTS2	-0,870	0,010608284
UBL7-AS1	-0,872	0,000583203
ATAD5	-0,873	5,98E-06
HR	-0,875	0,000400505
ARHGEF26	-0,877	0,029920932
RNF26	-0,877	4,36E-12
RP5-1120P11.1	-0,879	0,009949786
MELK	-0,879	6,78E-07

SLC17A7	-0,880	0,0304777<br>55
GLTSCR1	-0,880	0,0165369<br>7
WDR76	-0,881	7,45E-05
RBL1	-0,881	6,53E-11
LHX1	-0,882	0,0006434<br>56
TSPAN15	-0,883	0,0007174<br>55
CCDC85C	-0,884	4,58E-07
IGFBP5	-0,885	0,0490860<br>22
FZD1	-0,885	5,45E-05
SLC2A4RG	-0,887	1,91E-06
WNT7B	-0,887	0,0428023<br>05
KIF20B	-0,889	9,16E-06
PKN3	-0,889	4,42E-08
CCDC18	-0,891	3,35E-05
NRARP	-0,892	1,91E-08
DSN1	-0,895	1,49E-11
KIAA1524	-0,896	7,60E-06
PHGDH	-0,897	3,54E-16
TRIM9	-0,898	3,15E-05
WRAP53	-0,899	7,15E-07
DCAF15	-0,900	3,13E-09
HOXC6	-0,900	0,0454228<br>2
NEMP2	-0,900	0,0212495<br>69
MDC1	-0,900	2,19E-12
STIL	-0,900	6,99E-09
PTTG1	-0,902	5,07E-11
GPR162	-0,904	0,0357416<br>59
LINC00899	-0,905	0,0060320<br>26
DPYSL5	-0,905	0,0045262<br>28
BRIP1	-0,908	1,77E-05
DEPDC1B	-0,908	2,96E-08
NUDT1	-0,908	2,09E-12
ARHGAP19-<br>SLIT1	-0,908	1,06E-06
PDX1	-0,909	7,03E-05
APBB1	-0,910	0,0091919<br>98
STAG3	-0,910	0,0451338<br>85
ZCCHC12	-0,910	0,0376233<br>99

UBE2T	-0,911	8,80E-10
RAP1GAP	-0,911	0,0163224<br>37
E2F8	-0,911	2,36E-05
GAS1	-0,913	0,0208818<br>32
WHSC1	-0,914	1,32E-12
MASTL	-0,916	3,56E-08
ATAD2	-0,917	2,79E-07
CCDC136	-0,917	0,0252142<br>6
MCM4	-0,918	5,09E-09
CH507-<br>145C22.1	-0,920	0,0056120<br>36
NRM	-0,920	7,15E-12
CAMK4	-0,920	0,0071529<br>5
BMF	-0,923	0,0068092<br>37
POLA2	-0,924	2,23E-13
NCAPD3	-0,926	2,51E-15
AKNA	-0,926	5,32E-08
RCN3	-0,926	0,0021618<br>73
ESCO2	-0,926	1,97E-07
CDKN3	-0,926	1,31E-09
TERT	-0,927	0,0016049<br>17
ORC6	-0,928	1,66E-07
USP18	-0,928	0,0024311<br>57
DUT	-0,929	5,66E-11
DHFR	-0,929	1,38E-06
NPTX2	-0,935	1,59E-13
SAP30	-0,935	1,35E-10
ZGRF1	-0,936	1,90E-08
BARD1	-0,936	0,0003670<br>67
ATP1A3	-0,936	0,0010124<br>96
ARRB2	-0,937	3,11E-09
ID3	-0,937	0,0136125<br>18
TIMELESS	-0,937	1,82E-09
THEMIS2	-0,937	7,62E-05
CENPU	-0,937	4,08E-07
KANK2	-0,939	6,00E-11
LMNB2	-0,940	2,90E-14
PHF19	-0,940	2,89E-09
TTK	-0,941	1,15E-05
POLD1	-0,942	1,31E-11

ZNF724P	-0,942	0,0008168 43
CKLF-CMTM1	-0,942	0,0340423 79
KNTC1	-0,943	1,95E-05
DSCC1	-0,943	8,29E-07
IFI27L1	-0,943	7,62E-06
CLDN3	-0,943	0,0120949 18
HAUS4	-0,944	6,44E-05
PARM1	-0,947	0,0400432 66
FANCI	-0,949	3,43E-09
CARHSP1	-0,950	3,43E-09
UNC119	-0,951	0,0031923 12
IRF7	-0,951	2,23E-05
RACGAP1	-0,952	1,18E-11
POLE	-0,955	1,55E-09
MAP1A	-0,957	0,0351180 62
RRM2	-0,960	2,64E-07
DTL	-0,960	4,79E-07
SKA3	-0,962	6,05E-06
XRCC2	-0,962	1,47E-07
LIG1	-0,963	3,95E-13
ZWINT	-0,964	2,49E-07
RHEBL1	-0,964	1,48E-07
TLE2	-0,966	0,0267746 16
SAPCD1	-0,968	0,0352861 02
KIF18A	-0,969	5,42E-07
EPOR	-0,969	5,59E-06
UBALD2	-0,975	3,85E-16
C19orf68	-0,976	7,84E-05
PXMP2	-0,977	4,71E-09
LMCD1	-0,977	0,0307121 64
UBE2C	-0,978	1,90E-11
NCAPH2	-0,981	1,29E-07
C17orf53	-0,981	3,14E-07
CDK1	-0,982	1,38E-08
FBLN5	-0,983	0,0047093 22
CTD-2535L24.2	-0,983	0,0253579 09
PLEKHG4	-0,986	0,0073402 59
AURKA	-0,987	2,10E-11
LMNB1	-0,987	5,72E-14

VASH2	-0,988	0,0027483<br>43
SPATA3-AS1	-0,990	0,0377171<br>65
SUN2	-0,990	8,05E-14
SEMA6B	-0,990	0,0028453<br>46
KISS1R	-0,990	0,0069252<br>46
EXO1	-0,991	2,45E-06
FOXO6	-0,991	0,0007282<br>81
KIF11	-0,992	1,03E-09
OLMALINC	-0,993	0,0007929<br>76
HAUS8	-0,993	4,99E-07
ZP1	-0,999	0,0256408<br>82
KMT2E-AS1	-1,000	0,0068362<br>37
CDC6	-1,001	3,62E-08
TCF19	-1,003	3,85E-06
DDX11	-1,004	8,83E-19
CNTRL	-1,005	2,86E-06
MSH5	-1,006	3,40E-07
BRCA1	-1,007	7,76E-10
TMEM132E	-1,008	0,0001620<br>11
ACSBG1	-1,008	0,0037787<br>42
FANCA	-1,009	2,19E-12
TRIP13	-1,010	2,02E-12
TUBB4B	-1,010	1,44E-10
DIAPH3	-1,011	1,27E-09
SELM	-1,011	0,0268754<br>5
ARHGAP11B	-1,014	6,03E-10
PDZD4	-1,014	0,0258064<br>69
RP11-44M6.7	-1,015	0,0427708<br>53
LINC00634	-1,015	0,0137771<br>36
MIR210HG	-1,016	0,0010577<br>6
ASRGL1	-1,018	4,64E-05
NCK1-AS1	-1,018	3,05E-06
HMGN2	-1,019	2,83E-07
RP11-<br>*154J22.1*	-1,019	0,0381027<br>01
ARTN	-1,020	0,0017361<br>99

GINS4	-1,022	1,63E-05
PPDPF	-1,026	1,26E-09
RP11-445F12.1	-1,027	1,98E-07
BUB1B	-1,027	9,17E-11
CLDN23	-1,027	0,029940134
RFX2	-1,031	0,00460624
CLSPN	-1,031	5,13E-06
PMF1-BGLAP	-1,032	0,000109863
RP11-798M19.6	-1,034	0,003809184
CTSV	-1,038	1,53E-11
POLQ	-1,039	7,85E-08
HOXA6	-1,040	0,036572539
STARD8	-1,040	0,029985884
ATP2A3	-1,041	0,006313448
SYT7	-1,043	3,11E-05
KIF24	-1,044	1,45E-05
BCL2L12	-1,046	2,62E-06
NCAPG	-1,049	2,27E-08
CDCA2	-1,049	1,33E-09
SMC4	-1,051	3,04E-08
CENPF	-1,053	3,02E-07
CENPM	-1,057	8,31E-11
CENPH	-1,058	1,90E-13
MDK	-1,060	0,000737963
TUBA1B	-1,063	1,17E-14
ARHGAP11A	-1,064	3,57E-14
THAP10	-1,065	0,038195475
TPX2	-1,071	8,59E-13
RIBC2	-1,073	0,003098005
RAD51	-1,073	8,97E-11
MCM2	-1,077	2,69E-13
INCENP	-1,077	2,41E-15
SPA17	-1,077	5,90E-09
TONSL	-1,078	3,56E-08
UHRF1	-1,079	2,14E-05
TMEM145	-1,080	0,012523338
PLK4	-1,082	2,60E-14
CCNB1	-1,087	5,12E-15

NUP62	-1,087	3,55E-05
GNG7	-1,088	3,84E-06
CTD-2574D22.2	-1,088	0,017588953
SOWAHD	-1,089	0,010867767
SHCBP1	-1,089	2,05E-09
FOXD2	-1,092	0,006231614
FOXA2	-1,098	9,13E-05
YPEL4	-1,098	0,011371269
NDC80	-1,099	1,39E-08
CENPO	-1,100	2,05E-12
LZTS1	-1,109	0,006544687
HIRIP3	-1,113	7,49E-12
APOBEC3B	-1,114	1,56E-06
DEPDC1	-1,114	4,11E-10
BUB1	-1,115	5,14E-12
PPFIA4	-1,116	0,000257178
SNAI1	-1,116	8,47E-05
TMEM200B	-1,118	0,000259146
KIF26A	-1,118	0,000450606
GAS2L3	-1,119	7,93E-09
MYB	-1,119	0,000435966
FANCG	-1,119	2,41E-15
C18orf54	-1,120	4,95E-05
NEIL3	-1,121	4,29E-08
NCAPG2	-1,122	1,23E-14
BZRAP1	-1,122	0,000188955
ANLN	-1,123	1,13E-07
C16orf59	-1,124	2,83E-07
HES6	-1,125	4,97E-07
RAD51AP1	-1,126	1,63E-09
RP11-540D14.8	-1,128	0,013021689
BMP8B	-1,128	2,46E-05
CENPI	-1,128	2,24E-09
SGOL1	-1,131	7,77E-08
PKMYT1	-1,132	6,22E-09
CCDC150	-1,133	5,38E-05
HPSE	-1,134	1,80E-08
POC1A	-1,135	1,50E-08

SLC2A4	-1,136	0,0001815 28
CCDC163P	-1,140	0,0008686 29
IER5L	-1,143	3,40E-11
MCM7	-1,145	3,57E-14
BLM	-1,145	2,88E-08
RP11-480I12.5	-1,146	0,0134302 04
CEP55	-1,146	4,35E-10
CHAF1A	-1,147	1,30E-09
CDC45	-1,148	1,38E-11
MCM10	-1,150	2,02E-09
HCG27	-1,151	0,0380296 54
CDKN2D	-1,151	0,0002350 43
ESPNP	-1,153	0,0022033 8
RP11-22B23.1	-1,154	1,14E-12
HJURP	-1,157	1,62E-09
CDH23	-1,160	0,0035045 27
ASF1B	-1,160	7,27E-08
ORC1	-1,161	2,19E-12
FGFR3	-1,163	6,55E-06
HMMR	-1,165	1,49E-11
CCNB2	-1,166	4,10E-16
KIF4A	-1,168	1,14E-08
NUSAP1	-1,170	1,99E-13
CASC5	-1,171	1,12E-10
FANCD2	-1,173	5,87E-12
CACNA1I	-1,174	0,0141024 58
CDCA5	-1,176	6,23E-11
ZNF492	-1,177	0,0287824 06
RP11-302M6.4	-1,178	0,0066880 72
RP5-991G20.1	-1,179	6,10E-05
C5orf34	-1,181	4,29E-06
MND1	-1,182	1,49E-09
CENPE	-1,182	1,72E-07
ZNF331	-1,183	3,81E-08
ESAM	-1,183	0,0118460 82
FAM72B	-1,184	0,0006445 77
EDAR	-1,185	1,92E-06
CCNF	-1,187	1,25E-11

NOD2	-1,189	0,0025685<br>98
ENTPD8	-1,195	2,27E-06
NCAPD2	-1,197	1,39E-17
CHTF18	-1,201	3,50E-16
KIF15	-1,201	9,34E-10
DLL4	-1,203	0,0470218<br>38
TOP2A	-1,204	8,47E-11
TIGD3	-1,207	0,0356217<br>21
MCM5	-1,210	4,63E-12
PRRX2	-1,212	0,0035557<br>61
CORO1A	-1,212	0,0001361<br>02
GINS2	-1,214	3,66E-11
CARD8	-1,215	0,0006649<br>69
CCNA2	-1,215	2,12E-10
ENTPD2	-1,215	2,06E-06
CENPW	-1,221	3,80E-06
ZBTB12	-1,223	0,0048484<br>93
SHISA8	-1,223	0,0216526<br>43
TMPO-AS1	-1,223	3,40E-07
SKA1	-1,225	4,51E-08
FA2H	-1,226	0,0333639<br>33
CDCA8	-1,232	3,95E-13
DUSP9	-1,237	0,0004453<br>06
DLGAP5	-1,239	5,12E-13
ALDOC	-1,240	1,70E-08
BHLHA15	-1,240	0,0114093<br>6
MXD3	-1,246	1,38E-26
MLXIPL	-1,249	0,0011371<br>87
RMI2	-1,251	4,86E-17
FAM110A	-1,252	1,62E-09
C19orf57	-1,252	6,76E-06
TACC3	-1,252	5,93E-12
ASPM	-1,254	1,70E-11
SAPCD2	-1,256	1,58E-21
EME1	-1,257	1,43E-07
CKS1B	-1,262	1,38E-11
CABLES2	-1,263	1,96E-10
DBF4B	-1,266	1,93E-18
NUF2	-1,268	3,43E-12

HMGB2	-1,268	3,37E-17
ARHGEF39	-1,268	2,03E-13
CDC20	-1,269	1,33E-13
PROCA1	-1,270	1,15E-05
WDR62	-1,270	6,06E-10
BIRC5	-1,273	5,27E-15
SOX18	-1,274	0,0412490 39
MAFA	-1,274	0,0158832 62
SGK494	-1,280	0,0291562 24
OIP5	-1,286	5,27E-15
PSMC3IP	-1,287	1,81E-11
AC037459.4	-1,293	0,0324934 15
PRC1	-1,295	5,49E-16
PRR11	-1,298	8,37E-13
H2AFX	-1,299	1,62E-09
KIF22	-1,301	1,05E-18
MYBL2	-1,302	4,24E-16
NKX2-1	-1,304	9,05E-05
SPC25	-1,304	3,99E-11
ZBED1	-1,305	0,0221105 41
TRAIP	-1,306	5,44E-11
AC002116.7	-1,306	0,0001342 38
CDC25C	-1,307	5,01E-14
NCAPH	-1,308	3,95E-13
TLX1	-1,315	2,61E-05
RTN4RL2	-1,328	2,32E-08
H1FX	-1,329	2,96E-15
DDX12P	-1,329	5,75E-15
CDKN1C	-1,332	0,0004604 51
MKI67	-1,333	1,96E-11
DRP2	-1,334	0,0033036 49
VAMP1	-1,336	1,34E-09
AP001347.6	-1,336	0,0263182 79
MNS1	-1,340	0,0015198 6
FEN1	-1,344	8,89E-08
KIF14	-1,345	2,68E-10
AURKB	-1,350	2,74E-18
MPP2	-1,351	7,27E-09
CCL24	-1,352	0,0014276 96
CENPA	-1,357	1,90E-13

CIT	-1,357	1,35E-10
VASN	-1,358	9,72E-10
RNASEH2A	-1,358	2,05E-15
GPR19	-1,359	0,0002005<br>33
KIAA0319	-1,361	0,0035219<br>67
RGMA	-1,363	0,0034499<br>74
PTPRH	-1,363	6,78E-05
TP73	-1,364	5,14E-08
KIF20A	-1,372	7,07E-21
ESPL1	-1,376	1,59E-16
C14orf80	-1,376	5,59E-10
RECQL4	-1,378	1,75E-16
FAM72D	-1,384	3,21E-08
PBK	-1,392	7,59E-16
KIF21B	-1,392	0,0045139<br>43
YPEL1	-1,394	4,07E-07
E2F1	-1,395	3,31E-16
RP11-57A19.2	-1,398	0,0041558<br>56
COL6A2	-1,398	0,0008505<br>15
GTSE1	-1,399	1,98E-16
RP11-<br>*958N24.2*	-1,404	0,0084849<br>35
RP11-360L9.4	-1,408	0,0071347<br>24
PLK1	-1,412	3,65E-18
DNMT3B	-1,413	1,61E-09
DDIAS	-1,414	0,0003109<br>57
C21orf58	-1,414	1,77E-11
CKAP2L	-1,416	1,11E-18
C2orf48	-1,418	8,93E-05
TK1	-1,421	4,10E-16
MUC3A	-1,423	4,90E-05
C1QL1	-1,427	3,23E-07
CDCA3	-1,432	5,12E-24
NEK2	-1,437	3,54E-16
MFNG	-1,437	0,0192216<br>98
XRCC3	-1,442	4,94E-14
LRP4-AS1	-1,444	0,0386037<br>76
ITGA2B	-1,447	0,0067567<br>73
FAM132B	-1,451	9,66E-05
FBLN1	-1,457	8,66E-06

KIF2C	-1,461	3,94E-17
AC000068.5	-1,462	0,034384564
CDT1	-1,466	4,02E-13
SP8	-1,469	0,018130759
UBE2S	-1,470	5,36E-19
NEURL1B	-1,470	0,000687384
TICRR	-1,471	9,22E-10
IQGAP3	-1,474	5,79E-11
SLC30A3	-1,477	1,91E-09
HOXA-AS3	-1,485	0,000489246
YBX2	-1,493	6,88E-11
PSRC1	-1,497	3,92E-20
FAM64A	-1,501	6,88E-20
COL1A1	-1,504	2,66E-05
CD6	-1,505	0,024948736
KIFC1	-1,515	4,47E-18
NEURL1	-1,516	0,037834085
ARHGEF16	-1,517	4,68E-08
PIF1	-1,521	8,17E-19
E2F2	-1,524	9,22E-10
SOWAHA	-1,525	0,019139124
SPC24	-1,528	1,14E-15
ZNF821	-1,529	0,032558114
DERL3	-1,532	2,39E-10
SGSM1	-1,536	0,033070478
SERTAD4-AS1	-1,542	0,004526228
CH507-24F1.1	-1,547	4,45E-05
PTGER4	-1,547	0,017412291
PACSIN1	-1,549	0,014984214
FOXM1	-1,549	3,75E-19
KIF18B	-1,549	7,07E-21
AC004381.6	-1,550	3,21E-09
CACNA1G	-1,555	0,017692901
HMX3	-1,560	0,009416161
RAD54L	-1,563	2,02E-18
RAB3D	-1,567	0,010494421
NRTN	-1,567	3,78E-05

CACNA1H	-1,570	0,000402379
LIPE	-1,573	0,000369058
RDM1	-1,576	2,54E-14
NXPH4	-1,581	8,73E-19
TROAP	-1,582	1,36E-32
OAS3	-1,582	9,57E-08
SERTAD4	-1,583	3,85E-06
CSH2	-1,586	6,36E-14
ZNF511	-1,591	1,06E-08
CDH1	-1,592	1,11E-05
PTCHD2	-1,598	0,033070478
C2CD4C	-1,605	0,003039514
ATOH8	-1,609	0,011225874
CCDC74B	-1,609	0,001248554
EEF1E1P1	-1,613	0,04856559
COX6B2	-1,615	0,039690815
RNASE4	-1,623	0,010353826
RTKN2	-1,625	7,95E-09
CDKN2C	-1,631	6,69E-24
NMU	-1,631	5,44E-22
SPAG5	-1,631	2,27E-18
RP11-598P20.5	-1,634	0,040341122
RP11-544I20.2	-1,635	0,027875542
IL22RA1	-1,641	1,60E-12
MAPK8IP1	-1,650	1,51E-05
SUV39H1	-1,671	0,000623954
STMND1	-1,684	0,000188191
ERCC6L	-1,689	0,038198713
CABP1	-1,691	0,009488596
PCBP3	-1,697	0,017444075
CCDC121	-1,705	0,013703391
NRGN	-1,710	6,68E-22
TCAM1P	-1,721	0,001868583
INHBE	-1,734	0,011689797

CNTNAP2	-1,752	0,000893473
ARHGAP33	-1,809	1,02E-14
PALM3	-1,821	0,000746671
RP11-244M2.1	-1,841	6,36E-06
APOBEC3B-AS1	-1,851	0,035340587
HPCA	-1,882	0,022030824
KLHL32	-1,897	0,038123128
FXYD6	-1,907	9,05E-06
SLPI	-1,922	3,97E-06
LHX9	-1,935	0,002284995
FAM57B	-1,940	0,001615526
PTGDR2	-1,975	0,018752085
LCK	-1,976	7,13E-08
AP000251.3	-1,981	0,001086139
IRF8	-2,010	0,00108182
NXPH3	-2,016	0,004356701
GLI1	-2,052	0,009738975
KIF4B	-2,087	0,000680193
ARHGAP19	-2,095	4,06E-05
RP11-160O5.1	-2,117	6,21E-05
CCM2L	-2,177	0,008814173
RP11-195F19.30	-2,208	0,001021141
ZNF695	-2,223	1,71E-05
DMC1	-2,262	0,003324685
HPCAL4	-2,341	0,001311989
C2orf78	-2,842	3,27E-05
DMBX1	-3,310	2,49E-06

Table S5: **List of differentially regulated mRNAs**

gene symbol	log_2 fold change (+DOX vs –DOX) (*LINC01021* wt)	padj	log_2 fold change (+DOX vs –DOX) (*LINC01021* KO)	padj
NRGN	-1.123	1.95E-09	-1.710	6.68E-22
MAPK8IP1	-0.353	0.652157466	-1.650	1.51E-05
IL22RA1	-0.928	0.000279109	-1.641	1.60E-12
CDKN2C	-0.988	7.17E-09	-1.631	6.69E-24
TROAP	-1.004	8.81E-13	-1.582	1.36E-32
NXPH4	-0.962	5.24E-07	-1.581	8.73E-19
HOXA-AS3	-0.328	0.71225114	-1.485	0.000489246
SLC30A3	-0.824	0.004376498	-1.477	1.91E-09
PTPRH	-0.155	0.846732498	-1.363	6.78E-05
VASN	-0.124	0.826005056	-1.358	9.72E-10
VAMP1	-0.549	0.057494561	-1.336	1.34E-09
DDX12P	-0.780	3.28E-05	-1.329	5.75E-15
RTN4RL2	-0.595	0.048075346	-1.328	2.32E-08
DBF4B	-0.800	2.46E-07	-1.266	1.93E-18
CABLES2	-0.795	0.000235275	-1.263	1.96E-10
EME1	-1.384	6.50E-09	-1.257	1.43E-07
SAPCD2	-0.717	4.26E-07	-1.256	1.58E-21
MXD3	-0.785	2.23E-10	-1.246	1.38E-26
ALDOC	-0.250	0.563089131	-1.240	1.70E-08
CHTF18	-0.750	2.54E-06	-1.201	3.50E-16
ENTPD8	-0.437	0.261446985	-1.195	2.27E-06
RP11-22B23.1	-0.661	0.000310442	-1.154	1.14E-12
ESPNP	-0.187	0.835603109	-1.153	0.00220338
IER5L	-0.544	0.009645146	-1.143	3.40E-11
BMP8B	-0.433	0.312355726	-1.128	2.46E-05
INCENP	-0.640	1.66E-05	-1.077	2.41E-15
RP11-445F12.1	-0.107	0.842183945	-1.027	1.98E-07
SUN2	-0.568	0.000116367	-0.990	8.05E-14
CTD-2535L24.2	0.163	0.870534266	-0.983	0.025357909
C19orf68	-0.393	0.309495167	-0.976	7.84E-05
UNC119	0.354	0.536887179	-0.951	0.003192312
LMNB2	-0.518	0.000182831	-0.940	2.90E-14
KANK2	-0.385	0.037662817	-0.939	6.00E-11
THEMIS2	-0.276	0.526509943	-0.937	7.62E-05
AKNA	-0.437	0.049156954	-0.926	5.32E-08
NUDT1	-0.476	0.001502967	-0.908	2.09E-12
GPR162	-0.060	0.957671811	-0.904	0.035741659
PHGDH	-0.544	4.75E-06	-0.897	3.54E-16
PKN3	-0.450	0.025979604	-0.889	4.42E-08
CCDC85C	-0.290	0.30393861	-0.884	4.58E-07
GNAO1	-0.263	0.329903932	-0.869	2.51E-07
UQCC3	-0.364	0.083399991	-0.869	2.10E-08
ABHD8	-0.104	0.827812144	-0.859	3.16E-06

BCAM	-0.345	0.199391451	-0.858	2.67E-06
CTD-2033A16.3	0.098	0.895455629	-0.848	0.003419484
GRK6	-0.427	0.000466142	-0.832	9.68E-15
RCCD1	-0.492	0.000109999	-0.830	4.57E-13
MTFR2	-1.003	2.44E-09	-0.818	1.02E-06
NLGN2	-0.332	0.155875687	-0.816	7.15E-07
SH3RF3	-0.246	0.540583963	-0.814	0.000166036
REEP4	-0.534	2.54E-07	-0.804	6.91E-17
COX17	-0.466	0.001250141	-0.793	3.44E-10
TNNT1	-0.390	0.020254112	-0.791	7.06E-09
LRRC75A	-0.238	0.620314963	-0.790	0.000969878
POLR2J3	-0.138	0.819384481	-0.788	0.00186278
EMC9	-0.379	0.089376875	-0.782	2.22E-06
CBR3-AS1	-0.135	0.878761578	-0.781	0.024995866
CCHCR1	-0.419	0.006937078	-0.774	2.94E-09
EFNA3	-0.405	0.020254087	-0.771	4.52E-08
SP9	-0.330	0.27454922	-0.758	0.000104355
MNX1	-0.243	0.398226537	-0.754	7.14E-06
PAK4	-0.384	0.011569447	-0.752	2.06E-09
ATP8B3	-0.209	0.456281245	-0.741	3.20E-06
ODF2	-0.388	0.008172864	-0.738	1.85E-09
RHOV	-0.481	0.02595027	-0.737	1.57E-05
RP13-672B3.2	-0.337	0.412445203	-0.735	0.002799422
LMO2	-0.371	0.00017249	-0.725	8.98E-17
SSBP3	-0.406	0.011261912	-0.724	5.07E-08
AC253572.1	0.185	0.807879209	-0.714	0.039648048
SNHG19	0.001	0.998715579	-0.712	8.65E-07
RP11-77K12.1	0.301	0.642905052	-0.702	0.041829482
ARID5A	-0.287	0.252416219	-0.696	2.24E-05
CKB	-0.131	0.527560506	-0.693	7.23E-11
KCNK5	-0.307	0.036342857	-0.689	1.55E-09
THRA	-0.193	0.431830384	-0.681	1.06E-06
C11orf84	-0.347	0.009407828	-0.673	9.17E-10
TCF7	-0.322	0.022467784	-0.672	2.79E-09
CLASRP	-0.202	0.209973032	-0.671	1.12E-10
PBX1	-0.137	0.818553849	-0.664	0.011129802
C16orf93	-0.251	0.547019929	-0.661	0.004500798
NABP2	-0.440	2.56E-05	-0.651	1.02E-11
PER1	-0.018	0.97507512	-0.651	0.002269845
SP6	-0.143	0.757623355	-0.650	0.001984904
DOT1L	-0.183	0.488778294	-0.649	9.61E-06
PRR34-AS1	0.028	0.972329967	-0.648	0.032320863
KLHL25	-0.391	0.018603408	-0.645	1.32E-06
SLX4	-0.243	0.366111198	-0.634	0.000139111
CROCCP2_2	-0.090	0.871857391	-0.627	0.004383209
DCXR	-0.166	0.650682761	-0.627	0.000582894
THOC6	-0.299	0.026103248	-0.616	8.44E-09

CCDC9	-0.290	0.143480245	-0.615	1.18E-05
CROCCP2_1	-0.096	0.840631677	-0.614	0.001634172
PAFAH1B3	-0.258	0.080614241	-0.607	2.88E-08
PIGS	-0.135	0.64128938	-0.599	2.00E-05
LIN9	-0.745	8.08E-09	-0.598	3.72E-06
RP11-1212A22.1	0.155	0.567706606	-0.597	2.27E-05
PTMS	-0.217	0.22586937	-0.588	7.25E-07
TAF13	0.145	0.707418412	0.584	0.001240222
BTG1	0.138	0.705672548	0.595	0.000491225
ZNF181	0.209	0.385590141	0.598	3.68E-05
ZNF674	0.037	0.96021239	0.610	0.024987735
CD58	0.156	0.682862145	0.615	0.000858143
MMAA	0.167	0.747015397	0.618	0.011122394
ZNF613	-0.163	0.799651535	0.620	0.032952807
HMGA2	0.211	0.458416988	0.622	0.000150885
CROT	0.294	0.345734312	0.634	0.001320667
ZNF440	0.129	0.794362823	0.644	0.003598011
AP1S3	0.220	0.540353654	0.649	0.000768686
IMPACT	0.266	0.258138941	0.655	2.48E-05
RNF182	0.014	0.98169573	0.658	0.000958529
MT-ATP8	0.302	0.007020342	0.663	9.49E-13
CAV2	0.267	0.354005712	0.667	0.000186513
TAB3	0.435	0.000655661	0.671	3.12E-09
TEX9	-0.074	0.92391339	0.679	0.026318279
SNAP23	0.356	0.040260532	0.681	7.89E-07
SQRDL	0.333	0.363612867	0.690	0.002265639
LIPA	0.358	0.057008837	0.691	2.27E-06
TM7SF3	0.503	1.65E-08	0.693	1.79E-16
CSNK1G1	0.488	4.95E-06	0.706	1.16E-12
ZNF23	-0.052	0.918218251	0.710	7.24E-05
RIOK3	0.371	0.044905154	0.717	8.67E-07
ZNF585A	0.122	0.852521572	0.718	0.010553768
MOSPD1	0.249	0.124373317	0.719	3.24E-10
AP1S2	0.143	0.603112661	0.726	7.07E-08
SLC38A6	0.318	0.159121466	0.732	2.35E-06
SDSL	0.258	0.565886421	0.751	0.001914476
FAM221A	0.263	0.587125137	0.777	0.00223056
KLF6	0.237	0.360593608	0.781	4.51E-07
ZNF30	0.302	0.413075006	0.783	0.000336305
CYB5R2	0.300	0.356036007	0.785	4.78E-05
LYRM5	0.258	0.165232834	0.788	5.24E-10
CERS5	0.575	9.55E-05	0.798	3.12E-09
PERP	0.317	0.104864035	0.801	1.71E-08
TMEM106B	0.433	0.005539122	0.803	1.21E-09
WDR72	0.222	0.586276687	0.809	0.000132139
RP13-463N16.6	0.227	0.594426637	0.812	0.000241341
BIRC2	0.283	0.146215652	0.815	1.57E-09

TCEAL1	0.328	0.21917555	0.818	4.23E-06
TRNP1	0.724	9.41E-10	0.823	3.47E-13
CHURC1	0.295	0.294205602	0.834	2.65E-06
RORA	0.145	0.848020221	0.842	0.008957324
GABARAPL1	0.554	0.000172356	0.851	1.74E-10
SP140L	0.071	0.92103905	0.871	0.000958529
LPCAT2	0.426	0.052342075	0.879	2.83E-07
MTRNR2L12	-0.104	0.928776469	0.887	0.037722861
NACAP1	-0.067	0.932040523	0.887	0.002122135
PABPC3	0.133	0.878380584	0.887	0.015550326
RP11-421F16.3	0.512	0.00115133	0.894	5.40E-11
TMBIM4_1	0.160	0.800695116	0.905	0.000842653
IKBIP	0.495	3.96E-05	0.916	2.47E-17
LYPLAL1	0.348	0.191449472	0.932	2.32E-07
NEURL2	0.104	0.927629755	0.942	0.026724013
ANXA4	0.635	5.32E-06	0.969	5.54E-14
NR2F1	0.468	0.042811257	0.973	4.58E-08
BLOC1S2	0.567	0.003124342	0.975	3.11E-09
SLC16A2	0.470	0.042873486	0.987	4.18E-08
C15orf52	0.940	2.68E-18	1.001	2.66E-21
CCNE1	0.750	4.87E-08	1.001	4.73E-15
PUS3	0.483	0.066797861	1.013	4.42E-07
ADAM23	0.639	0.005601723	1.022	2.20E-07
TRIAP1	0.448	0.082443016	1.051	4.72E-08
PTP4A1	0.627	0.00082935	1.070	1.11E-10
CPEB2	0.389	0.656286738	1.072	0.012447201
PLK2	0.721	1.31E-07	1.079	4.86E-17
ETS1	0.518	0.039351136	1.090	3.63E-08
PLAU	0.556	0.043400516	1.117	1.36E-07
HGSNAT	0.767	0.000295421	1.127	2.23E-09
SERPINE2	0.489	0.002156135	1.165	5.56E-18
FAS	0.656	7.35E-05	1.166	3.09E-15
TSC22D1	0.666	1.89E-05	1.170	1.76E-16
CEP170P1	-0.238	0.829412856	1.171	0.013586991
SDCBP2	0.068	0.951154282	1.192	0.001898289
SPTBN5	0.990	3.77E-07	1.203	3.00E-11
PTPRZ1	0.647	0.544608989	1.207	0.04343326
NLRP3	0.501	0.330872844	1.208	0.000161344
THNSL1	0.041	0.972584153	1.213	0.004088068
NLRP1	0.937	1.17E-06	1.259	3.96E-12
GULP1	0.736	0.000614096	1.259	2.74E-11
CAV1	0.276	0.511492721	1.266	9.45E-09
CTSB	0.905	2.48E-08	1.284	7.99E-17
SOCS2	0.780	0.004234742	1.306	1.28E-08
PMAIP1	0.492	0.009720426	1.316	6.72E-18
DDX60L	0.763	0.043400516	1.331	5.71E-06
HPGD	0.625	0.062240712	1.361	1.11E-07

CD163L1	0.943	0.000219269	1.412	7.80E-10
ITGB8	0.609	0.110259301	1.413	4.80E-07
AC006369.2	-0.382	0.791799704	1.431	0.034399157
GADD45A	1.010	5.62E-10	1.444	7.07E-21
PORCN	1.047	3.24E-06	1.471	2.19E-12
URGCP-MRPS24	-0.414	0.579675525	1.484	0.000201784
EDN1	0.859	0.008486943	1.491	6.29E-08
CDH13	1.071	1.66E-19	1.567	8.76E-43
TIGAR	1.089	1.41E-08	1.581	2.04E-18
AFF3	-0.239	0.881760856	1.596	0.013586991
TGFB2	0.394	0.690702535	1.650	0.000808911
SLC6A12	2.266	6.36E-05	1.763	0.009249002
BIRC3	0.684	0.132418039	1.779	3.61E-08
ADRB2	-0.175	0.913945793	1.837	0.00477735
GJB3	1.406	0.00037985	1.907	7.75E-08
DRAM1	1.848	5.58E-24	2.096	3.87E-32
RPS27L	1.958	1.35E-54	2.254	4.20E-73
FDXR	2.032	4.11E-47	2.262	2.43E-59
PLTP	1.388	0.004750381	2.293	5.34E-08
RP11-529A4.4	1.322	0.023168324	2.385	2.66E-07
DDX47	0.576	0.476541631	2.401	4.12E-07
TSPAN1	1.847	1.45E-07	2.477	9.23E-15
CTGF	1.896	3.12E-09	2.624	9.13E-19
AK5	1.116	0.058113727	2.756	8.64E-10
FLG-AS1	2.342	6.23E-26	2.989	4.14E-43
DKK1	1.833	2.09E-09	3.120	1.42E-27
DQX1	2.077	0.00039358	3.267	6.29E-11
KRTAP3-1	2.090	2.25E-08	3.286	7.40E-22
KRTAP2-3	3.584	0.000853193	5.306	2.52E-08

Table S6: List of differentially regulated transcript isoforms

Transcript name	Transcript ID	$\log_2$ fold change (+DOX vs –DOX) (*LINC01021* wt)	padj	$\log_2$ fold change (+DOX vs –DOX) (*LINC01021* KO)	padj
MSH6_16	ENST00000614496	-0,43339	0,70928	-4,29743	0,00000
YWHAQ_5	ENST00000446619	1,08790	0,26409	-2,53626	0,00047
RDX_22	ENST00000530131	1,20652	0,19273	-2,22225	0,00429
SPTBN2_2	ENST00000533211	1,88280	0,00177	-1,36160	0,05264
OSBPL8_5	ENST00000393250	1,39029	0,03064	-1,62705	0,01077
SRCAP_3	ENST00000262518	0,01524	0,99225	-2,86245	0,00000
MBTPS1_11	ENST00000569770	1,01225	0,32175	-1,78789	0,03189
FARSA_10	ENST00000592662	0,68174	0,20555	-2,08728	0,00000
MYO18A_1	ENST00000533112	0,48524	0,71196	-2,28361	0,00134
SPINT1_10	ENST00000563656	1,16047	0,06074	-1,60403	0,00515
RPS24_16	ENST00000482069	0,58736	0,42275	-2,11183	0,00001
JPT2_10	ENST00000561516	1,27369	0,10954	-1,42269	0,07010
BMP7_9	ENST00000450594	0,24220	0,85618	-2,43356	0,00003
ATP5MG_7	ENST00000529770	0,71131	0,39863	-1,94784	0,00088
SH3GL1_5	ENST00000417295	0,68274	0,38352	-1,82375	0,00097
FABP5P7_1		0,99026	0,18899	-1,49408	0,02426
RPN2_7	ENST00000462163	0,66130	0,39606	-1,75319	0,00117
PQBP1_12	ENST00000376563	1,43828	0,01331	-0,96360	0,17345
NIFK_4	ENST00000498570	1,71267	0,00028	-0,54817	0,43734
PTPA_24	ENST00000355007	1,24548	0,01044	-0,99918	0,07476
CLK2_4	ENST00000368361	0,52637	0,51239	-1,70737	0,00131
FOSL2_3	ENST00000436647	0,40891	0,67488	-1,81061	0,00067
ARHGAP12_7	ENST00000375245	1,19682	0,01168	-1,01653	0,05731
CTDSP2_2	ENST00000548823	0,90445	0,02439	-1,29594	0,00044
ACVR1B_7	ENST00000542485	1,05744	0,05884	-1,13292	0,04357
UFC1_5	ENST00000483191	0,63789	0,28805	-1,54477	0,00033
ARHGAP5_3	ENST00000396582	0,70881	0,35296	-1,46923	0,01128
ARHGAP5_7	ENST00000556611	0,91774	0,15730	-1,24415	0,03933
ZFAND3_1	ENST00000373391	1,84486	0,00003	-0,30740	0,69636
ACTG1_4	ENST00000570382	0,65688	0,26755	-1,48092	0,00120
DHCR7_11	ENST00000531364	0,15689	0,90438	-1,97017	0,00019
RPL29_2	ENST00000475248	0,50072	0,23716	-1,62439	0,00000
P4HB_9	ENST00000574914	1,39449	0,00549	-0,70603	0,29004
PSMB10_3	ENST00000570985	0,95354	0,13449	-1,12259	0,07116
HDGF_10	ENST00000469145	0,50879	0,52184	-1,52612	0,00352
WDR6_11	ENST00000610967	1,35577	0,00138	-0,64601	0,24265
RPS27A_5	ENST00000402285	0,30422	0,63702	-1,69290	0,00000
ENO2_9	ENST00000541477	0,88080	0,17635	-1,10657	0,06971

PML_21	ENST00000435786	1,05721	0,01112	-0,92709	0,04475
TMEM94_23	ENST00000579208	0,59785	0,28709	-1,36782	0,00150
RACK1_24	ENST00000506312	0,65842	0,01560	-1,27711	0,00000
FAM72A_3	ENST00000367128	-0,00869	0,99308	-1,93365	0,00000
CLK1_15	ENST00000409403	0,31066	0,67222	-1,58210	0,00010
LUC7L2_4	ENST00000435096	0,33614	0,70830	-1,55072	0,00143
PLEKHA8_8	ENST00000449726	1,57364	0,00002	-0,31043	0,63450
ANAPC5_17	ENST00000541887	0,86462	0,00291	-0,99932	0,00105
LBH_5	ENST00000406087	1,39733	0,00386	-0,45761	0,52879
ERI3_2	ENST00000452396	0,73232	0,17515	-1,09412	0,02169
CRABP2_3	ENST00000368221	0,20309	0,77924	-1,60762	0,00000
DLG3_7	ENST00000489733	1,31118	0,00214	-0,48885	0,40523
UQCR11_1	ENST00000593264	-0,38205	0,61964	-2,17052	0,00000
DDX3X_17	ENST00000642597	0,47598	0,43157	-1,31202	0,00136
MAVS_2	ENST00000416600	0,66316	0,28255	-1,10301	0,03136
RNF167_16	ENST00000575111	0,77995	0,06445	-0,98376	0,01279
APPL1_9	ENST00000482800	-0,06848	0,95396	-1,78433	0,00005
FLNA_18	ENST00000369850	2,22888	0,00000	0,52852	0,28844
POLR2F_10	ENST00000651769	0,16841	0,80725	-1,52508	0,00000
CFL1_9	ENST00000527344	0,06752	0,94779	-1,59135	0,00001
ERBB3_15	ENST00000415288	0,36994	0,61408	-1,27519	0,00423
CDK2_3	ENST00000354056	-0,12536	0,87195	-1,76169	0,00000
CTNNB1_8	ENST00000645982	1,26313	0,00001	-0,34892	0,44824
OAZ1_1	ENST00000581150	0,14433	0,76038	-1,46254	0,00000
RPL36_7	ENST00000579649	-0,25709	0,74052	-1,85128	0,00000
FBXO28_2	ENST00000424254	1,44173	0,00002	-0,15117	0,82113
ATP2B1_6	ENST00000428670	0,86434	0,00007	-0,72769	0,00352
SC5D_7	ENST00000527762	-0,00759	0,99522	-1,57367	0,00005
RPL37A_9	ENST00000456586	0,46569	0,30341	-1,08482	0,00154
STOML2_5	ENST00000452248	0,57719	0,10730	-0,96042	0,00255
TMEM120A_14	ENST00000439537	1,73209	0,00000	0,19774	0,73101
INO80_1	ENST00000558357	0,58054	0,10469	-0,94887	0,00289
ATP5F1E_2	ENST00000395663	0,08971	0,90445	-1,43439	0,00000
USF2_9	ENST00000379134	0,87870	0,00847	-0,64021	0,10353
SLC2A1_1	ENST00000415851	0,65710	0,07408	-0,84680	0,01731
SEC23A_8	ENST00000545328	0,82644	0,01936	-0,66493	0,09627
PARD3_5	ENST00000374790	0,65018	0,04626	-0,83808	0,00806
TM9SF1_3	ENST00000524835	0,50481	0,29645	-0,94321	0,01494
ACTN4_2	ENST00000424234	0,68666	0,01633	-0,74645	0,01260
FAM91A1_2	ENST00000521166	0,82151	0,03308	-0,58789	0,19234
DUT_2	ENST00000558472	0,51756	0,04330	-0,85079	0,00023
HECTD1_17	ENST00000399332	0,78173	0,00000	-0,56355	0,00479
GOLGB1_3	ENST00000393667	1,07582	0,00000	-0,25335	0,46741
GMPPA_10	ENST00000373908	0,26391	0,50645	-1,04625	0,00002
CIC_8	ENST00000160740	-0,60417	0,04724	-1,90635	0,00000
POLR2F_13	ENST00000492213	0,11114	0,88129	-1,14571	0,00026
EEF1A1_8	ENST00000488500	1,05196	0,00081	-0,20076	0,72181

PSME3_10	ENST00000589469	0,82316	0,00116	-0,41782	0,21188
BSG_6	ENST00000573216	0,19509	0,74603	-1,02950	0,00111
SRP54_14	ENST00000555557	0,50950	0,06863	-0,71194	0,00722
RPL8_6	ENST00000533397	0,24992	0,58581	-0,96515	0,00046
LYN_4	ENST00000519728	1,04157	0,00039	-0,16172	0,77348
KIF2A_4	ENST00000401507	-0,00798	0,99125	-1,20393	0,00000
ADNP_3	ENST00000396032	-0,24264	0,54606	-1,43610	0,00000
NHP2_4	ENST00000314397	0,47393	0,03858	-0,71321	0,00066
GORASP1_11	ENST00000452389	0,29578	0,50442	-0,88762	0,00239
RN7SL1_1		0,34908	0,19482	-0,83236	0,00009
AC022966.1_24	ENST00000312010	1,02809	0,00000	-0,15312	0,70214
NAB1_6	ENST00000409581	0,50763	0,09382	-0,59078	0,04459
CLINT1_4	ENST00000523908	0,47236	0,07004	-0,62034	0,01297
UBB_1	ENST00000535788	0,18931	0,61374	-0,88120	0,00004
BCYRN1_1		0,28933	0,50304	-0,77457	0,00819
RERE_17	ENST00000400908	0,54418	0,04843	-0,49800	0,09577
LEF1_2	ENST00000438313	0,31851	0,40232	-0,71983	0,01080
NSUN2_5	ENST00000506139	0,29042	0,40570	-0,71449	0,00419
SARS1_3	ENST00000477544	0,53878	0,06357	-0,46569	0,13890
ZNF646_2	ENST00000300850	0,55563	0,02092	-0,44006	0,11083
HSF2_2	ENST00000368455	0,32128	0,29770	-0,67410	0,00553
ABR_8	ENST00000572585	0,54098	0,00984	-0,44448	0,06406
AAMP_2	ENST00000248450	0,21829	0,57957	-0,75272	0,00203
OSBPL9_25	ENST00000531828	0,48365	0,05420	-0,48579	0,06598
RN7SL2_1		0,24078	0,34976	-0,71966	0,00006
STK38L_4	ENST00000536093	0,34458	0,25809	-0,57437	0,02175
MAN2A2_5	ENST00000560451	0,73294	0,00000	-0,18020	0,35733
ACTG1_1	ENST00000644774	0,09793	0,85073	-0,80903	0,00061
MBTD1_3	ENST00000586178	0,62136	0,00249	-0,26203	0,37212
ACTR3_8	ENST00000443297	-0,02511	0,96233	-0,90000	0,00000
PRRC2A_6	ENST00000376033	0,23545	0,43536	-0,61337	0,00360
HNRNPL_3	ENST00000221419	-0,16531	0,64083	-0,98406	0,00000
RPS9_10	ENST00000626547	0,29405	0,22550	-0,50811	0,01269
RPS2_2	ENST00000533161	0,31062	0,22741	-0,49135	0,02666
POLR2L_1	ENST00000534030	0,12496	0,76558	-0,67578	0,00168
SNHG19_1	ENST00000563192	0,02846	0,95321	-0,77117	0,00001
CNTROB_7	ENST00000380262	-0,17306	0,55576	-0,96260	0,00000
EIF4G1_26	ENST00000346169	0,00632	0,99133	-0,77067	0,00002
TKT_12	ENST00000450814	0,27729	0,12949	-0,49631	0,00182
ARID1A_10	ENST00000324856	0,14921	0,63494	-0,60579	0,00100
RPL27A_2	ENST00000526562	0,18543	0,51323	-0,56933	0,00220
KHSRP_9	ENST00000619396	0,14814	0,65747	-0,57129	0,00358
STIP1_7	ENST00000538945	0,06540	0,85497	-0,64338	0,00004
SLK_4	ENST00000369755	0,06047	0,88426	-0,63795	0,00022
MIEF1_13	ENST00000433117	0,31824	0,11953	-0,37809	0,05756
NPM1_10	ENST00000523339	0,02934	0,95054	-0,66351	0,00011
LUC7L3_21	ENST00000504065	0,33332	0,05028	-0,32623	0,06995

BCOR_7	ENST00000378444	-0,27778	0,20261	-0,92919	0,00000
DAZAP1_9	ENST00000592453	-0,36828	0,01749	-0,94285	0,00000
KDM5C_6	ENST00000404049	0,04083	0,90432	-0,53270	0,00008
ATN1_3	ENST00000396684	0,06070	0,83534	-0,49512	0,00020
COX5A_4	ENST00000564811	0,16559	0,29770	-0,38948	0,00105
ACTN4_8	ENST00000588618	0,36556	0,00024	-0,17049	0,21806
PLXNB2_5	ENST00000359337	1,08729	0,00000	0,59102	0,00000
PTMS_5	ENST00000309083	-0,14940	0,40542	-0,61979	0,00000
U2AF2_1	ENST00000450554	0,02737	0,91514	-0,39010	0,00010
LRPPRC_1	ENST00000260665	0,22385	0,01590	0,56756	0,00000
RPL4_10	ENST00000564439	0,00204	0,99522	0,46097	0,00007
SERP1_6	ENST00000239944	-0,11200	0,47798	0,37792	0,00022
CCNC_16	ENST00000520429	-0,07943	0,71694	0,41683	0,00036
TEAD1_1	ENST00000526600	-0,30235	0,00741	0,20927	0,12847
DDR1_46	ENST00000452441	0,16654	0,36749	0,67929	0,00000
PLS3_1	ENST00000539310	-0,07964	0,77160	0,44662	0,00141
MTATP8P1_1		0,18255	0,32418	0,74439	0,00000
TXNDC9_5	ENST00000264255	-0,13023	0,61740	0,45176	0,00440
CD109_3	ENST00000287097	0,02860	0,94110	0,61932	0,00002
B2M_8	ENST00000544417	-0,35194	0,00762	0,24058	0,13565
NAB1_7	ENST00000337386	-0,30009	0,03940	0,34642	0,01919
BIRC2_8	ENST00000227758	0,25291	0,10667	0,90218	0,00000
H3-3B_1	ENST00000593254	-0,24154	0,23839	0,44743	0,00803
CRYZ_7	ENST00000340866	-0,13027	0,69455	0,56888	0,00233
IMPDH1_17	ENST00000348127	-0,47647	0,02171	0,27710	0,27776
RAB11A_3	ENST00000564910	-0,17299	0,56141	0,59531	0,00131
SUMO1_2	ENST00000392244	-0,59388	0,00007	0,17547	0,46372
AHR_4	ENST00000242057	-0,07002	0,89014	0,74565	0,00038
NR2F2_5	ENST00000421109	0,09221	0,85026	0,91949	0,00002
SNX10_8	ENST00000338523	-0,52964	0,01329	0,31211	0,25376
NPEPPS_22	ENST00000530514	-0,18278	0,54840	0,66348	0,00055
NOLC1_8	ENST00000603946	-0,39245	0,00097	0,45779	0,00031
LETMD1_31	ENST00000262055	-0,04130	0,94423	0,82613	0,00013
RHOC_19	ENST00000468093	0,22889	0,48616	1,09700	0,00000
HUWE1_2	ENST00000612484	-0,34750	0,19163	0,54751	0,01919
RNMT_9	ENST00000383314	-0,38816	0,16461	0,51236	0,04820
RTL10_3	ENST00000407472	-0,13610	0,71887	0,77087	0,00009
NOL7_2	ENST00000474485	-0,46284	0,03674	0,44748	0,06177
ATP5F1B_4	ENST00000552959	-0,11219	0,79886	0,80712	0,00014
C1orf43_10	ENST00000350592	-0,21866	0,46179	0,70140	0,00033
INTS13_7	ENST00000538155	-0,42437	0,12172	0,49877	0,06444
FASTKD2_4	ENST00000236980	-0,22142	0,50367	0,70580	0,00119
SPATA13_12	ENST00000382095	-0,30059	0,03472	0,63853	0,00000
RASSF8_6	ENST00000541490	-0,42377	0,09806	0,51989	0,03964
CHMP4A_4	ENST00000530996	-0,14821	0,73921	0,80649	0,00059
MPDU1_21	ENST00000396501	-0,36123	0,22894	0,59811	0,02013
MTPAP_5	ENST00000488290	-0,23076	0,56961	0,73504	0,00464

ITM2B_9	ENST00000648898	-0,07599	0,89186	0,89743	0,00007
NPC2_6	ENST00000555619	-0,17177	0,65219	0,81155	0,00016
DLG1_20	ENST00000659716	1,08990	0,00000	2,09255	0,00000
SEPTIN8_6	ENST00000378706	-0,56241	0,05668	0,45565	0,17887
IVNS1ABP_1	ENST00000367498	-0,57522	0,03550	0,45026	0,15039
NUP153_1	ENST00000262077	-0,36513	0,29761	0,67175	0,01849
GOSR2_8	ENST00000640051	-0,10440	0,84921	0,93711	0,00013
LGALS8_7	ENST00000323938	-0,24577	0,49571	0,79707	0,00076
RACK1_3	ENST00000511473	-0,26496	0,30772	0,80186	0,00002
SLC6A6_8	ENST00000622186	-0,11425	0,76509	0,97466	0,00000
CLDN12_13	ENST00000394605	-0,52775	0,03823	0,58673	0,02546
MSH6_7	ENST00000445503	-0,41023	0,02389	0,70606	0,00003
FHOD3_5	ENST00000592930	-0,41812	0,12164	0,71385	0,00179
LEPROTL1_2	ENST00000321250	-0,71244	0,00887	0,43712	0,19340
MBNL1_4	ENST00000463374	-0,66739	0,00440	0,49196	0,08050
SAMD4A_12	ENST00000554335	-0,63323	0,00523	0,54450	0,03524
AC006064.6_1	ENST00000646322	-0,03527	0,95228	1,14690	0,00000
DLD_14	ENST00000415325	-0,31818	0,22741	0,88466	0,00001
DEK_1	ENST00000651992	-0,80713	0,00516	0,42049	0,27873
WDR77_1	ENST00000449340	-0,16489	0,78204	1,07397	0,00027
RNF13_11	ENST00000361785	-0,04216	0,96207	1,20202	0,00016
EPCAM_3	ENST00000490733	-0,78836	0,00523	0,46998	0,19290
OFD1_1	ENST00000380550	-0,18064	0,78675	1,08207	0,00138
ALAS1_3	ENST00000310271	-0,31622	0,55163	0,95224	0,00634
RAN_8	ENST00000541630	-0,52858	0,00065	0,75103	0,00000
ALDH3A2_15	ENST00000176643	-0,77807	0,02306	0,51198	0,22294
SDC1_3	ENST00000381150	-0,36787	0,47136	0,93573	0,00805
MYO5A_13	ENST00000356338	-0,43937	0,32175	0,87830	0,00977
ZNF302_10	ENST00000505365	-0,22519	0,61001	1,10141	0,00001
ENOPH1_3	ENST00000509635	-0,33378	0,47825	1,03938	0,00090
DNAJC10_7	ENST00000491074	0,25297	0,57256	1,63180	0,00000
TMBIM4_5	ENST00000633367	-0,39197	0,33467	1,00029	0,00058
HSF2_3	ENST00000452194	-0,40624	0,44454	0,98606	0,00927
FMR1_18	ENST00000440235	-0,95340	0,00750	0,44053	0,37171
MYO1B_8	ENST00000392316	-0,31757	0,53955	1,08300	0,00097
SMARCAD1_1	ENST00000354268	-0,54624	0,17965	0,85792	0,01775
MAPK11_3	ENST00000330651	-0,35047	0,49730	1,06868	0,00157
NEK1_6	ENST00000510533	-1,11335	0,00180	0,31001	0,59750
USP9X_4	ENST00000324545	-0,43251	0,32226	1,00057	0,00245
PSMC5_18	ENST00000375812	-0,87704	0,00006	0,56477	0,03170
ZCCHC14_3	ENST00000268616	-0,08973	0,92833	1,36575	0,00045
RRBP1_6	ENST00000360807	-0,77240	0,01465	0,69420	0,04829
ZFP90_17	ENST00000398253	-0,36717	0,53636	1,10015	0,00488
INTS6_17	ENST00000497989	-0,53575	0,18701	0,93654	0,00720
SON_19	ENST00000436227	-0,93071	0,00025	0,55082	0,09347
METTL26_2	ENST00000301686	-0,57487	0,05482	0,91090	0,00080
AMZ2_2	ENST00000392720	-0,21437	0,75128	1,28718	0,00020

ATP6AP2_20	ENST00000447485	0,21998	0,69803	1,73302	0,00000
URI1_12	ENST00000360605	-0,07617	0,87048	1,44144	0,00000
EDRF1_6	ENST00000356792	-0,74698	0,05130	0,78599	0,04940
MYL12B_5	ENST00000400175	-1,19873	0,00088	0,33431	0,56751
STX16_12	ENST00000371132	-0,46133	0,25873	1,07179	0,00067
C5orf24_1	ENST00000504727	-0,40868	0,54154	1,16265	0,00891
NXN_4	ENST00000571684	-0,26363	0,73670	1,32962	0,00167
NAT1_5	ENST00000520546	-0,88523	0,04667	0,71830	0,15262
RPL37_4	ENST00000509877	-0,29019	0,67067	1,32090	0,00060
HDAC8_23	ENST00000373573	-0,76907	0,03613	0,85276	0,02606
HSPA9_10	ENST00000297185	-0,77447	0,13062	0,84951	0,09627
ELMSAN1_10	ENST00000476562	-0,43535	0,10054	1,19235	0,00000
NUMB_9	ENST00000359560	-0,11141	0,90238	1,54823	0,00002
ETS1_3	ENST00000392668	-0,21024	0,66332	1,45321	0,00000
SORBS1_14	ENST00000649911	-0,64962	0,12581	1,04701	0,00574
ERCC6L2_12	ENST00000320486	-0,63339	0,28854	1,06901	0,03243
NAPRT_10	ENST00000426292	-1,05843	0,01365	0,67318	0,20416
WDYHV1_8	ENST00000518125	-1,06378	0,01599	0,70105	0,19370
MSL3_8	ENST00000649684	-0,84536	0,12049	0,96629	0,07318
MARCHF8_6	ENST00000453424	0,60098	0,24215	2,45723	0,00000
MAN2A2_6	ENST00000558161	-0,69950	0,11548	1,17119	0,00318
POLR2B_13	ENST00000639658	-0,12361	0,92212	1,75553	0,00044
NXPE3_6	ENST00000273347	-0,52787	0,37440	1,35394	0,00180
PUM2_1	ENST00000319801	-0,79375	0,10858	1,09000	0,01813
NFAT5_3	ENST00000393742	-0,17764	0,85169	1,70859	0,00005
MADD_2	ENST00000402799	-0,80975	0,16170	1,08401	0,04383
RPS20_6	ENST00000519606	-0,18565	0,83667	1,71270	0,00002
PCID2_6	ENST00000375479	-1,82139	0,00005	0,14943	0,87323
LYN_2	ENST00000520220	-1,29138	0,00003	0,69709	0,08843
PTPRA_5	ENST00000356147	-1,34651	0,00732	0,67048	0,32462
ZNF706_1	ENST00000517844	-0,51256	0,54840	1,51028	0,00776
TUT4_5	ENST00000371544	-1,49377	0,00257	0,55346	0,44874
TMEM33_8	ENST00000513702	-0,41173	0,43996	1,75573	0,00000
MAGT1_2	ENST00000358075	0,24121	0,82570	2,41732	0,00000
PHB_5	ENST00000512041	-0,16177	0,88296	2,01896	0,00001
RBBP4_17	ENST00000458695	-1,08056	0,00211	1,10616	0,00436
GOLGA7_6	ENST00000520817	-0,71631	0,32834	1,51273	0,00747
RPL37A_10	ENST00000359681	-0,19954	0,84653	2,04093	0,00001
PLEKHM2_2	ENST00000642363	-1,32908	0,02599	0,94929	0,17982
CLINT1_3	ENST00000530742	-1,04286	0,00986	1,23720	0,00321
PTK7_5	ENST00000481273	-0,71699	0,07371	1,57178	0,00000
DBNL_12	ENST00000468694	-0,81164	0,14124	1,49396	0,00158
GCH1_7	ENST00000491895	-0,68214	0,27733	1,66528	0,00054
ADNP_4	ENST00000349014	0,79073	0,12580	3,13977	0,00000
TPM4_20	ENST00000588032	-0,88157	0,06378	1,47314	0,00071
DIPK2A_1	ENST00000441925	-0,25188	0,83582	2,18337	0,00016
GOLGA8B_1	ENST00000342314	-1,31896	0,00999	1,13944	0,05000

CTDNEP1_10	ENST00000572043	-0,05609	0,94489	2,41490	0,00000
KDM3B_1	ENST00000542866	-0,49369	0,64523	2,01066	0,00150
GUF1_3	ENST00000506793	-0,45694	0,50447	2,05777	0,00000
CSDE1_5	ENST00000261443	-0,61778	0,53470	1,93106	0,00277
ARHGAP12_2	ENST00000344936	-0,27958	0,82635	2,33897	0,00009
COPA_17	ENST00000648501	-2,10004	0,00000	0,55585	0,31645
STOML2_2	ENST00000619795	-1,05081	0,07781	1,81767	0,00067
CEP41_16	ENST00000223208	-1,68368	0,00000	1,18751	0,00347
CEMIP2_6	ENST00000377066	-0,83626	0,17072	2,32016	0,00000
RPL22_7	ENST00000497965	-2,18231	0,00025	1,19984	0,12466
ITSN2_11	ENST00000622089	-0,97826	0,17373	2,74489	0,00000
PQBP1_18	ENST00000472742	-1,87884	0,03610	2,07228	0,02495
NSRP1_8	ENST00000612959	-1,84801	0,00168	2,32271	0,00016
